# A genome-wide association study to elucidate the genetic basis of the cottonseed protein content in Upland cotton (*Gossypium hirsutum* L.)

**DOI:** 10.1270/jsbbs.24092

**Published:** 2026-01-15

**Authors:** Nijiang Ai, Haihong Zhao, Guoli Feng, Lei Du, Bo Li, Hongliang Liu, Zhihong Zheng, Ningshan Wang, Xiaoyun Tang, Haiqiang Gao, Kewei Ning, Chengqi Li

**Affiliations:** 1 Shihezi Academy of Agricultural Sciences, Shihezi, Xinjiang, 832000, China; 2 Life Science College, Yuncheng University, Yuncheng, Shanxi, 044000, China

**Keywords:** cottonseed protein content (CPC), quantitative trait nucleotide (QTN), QTN-by-environment interaction (QEI), QTN-by-QTN interaction (QQI), candidate gene

## Abstract

Cottonseeds are rich in proteins with high nutritional value. In this study, a genome-wide association study involving six methods was performed to dissect the genetic architecture of the cottonseed protein content (CPC) in Upland cotton (*Gossypium hirsutum* L.). The CPC exhibited typical characteristics of a quantitative trait. The six methods revealed 1, 4, 4, 31, 10, and 30 CPC-related quantitative trait nucleotides (QTNs), among which 17 were detected by at least two methods. Notably, TM40095 on A12 and TM59869 on D06 were detected by three methods, thus being considered stable QTNs. Five QTN-by-environment interactions (QEIs) and 29 QTN-by-QTN interactions (QQIs) were detected. The regions flanking the SNPs of two stable QTNs, five QEIs, and four significant QQIs included 49, 174, and 269 candidate genes, respectively. A functional enrichment analysis indicated that 12 and 48 non-redundant genes related to the two stable QTNs and five QEIs, respectively, were associated with significantly enriched functions. Moreover, eight protein (gene)–protein (gene) interactions were predicted. According to RNA-seq expression data, *GH_D06G1049* (related to QTNs) and *GH_A06G1663*, *GH_D13G0601*, and *GH_A05G0236* (related to QEIs) were preferentially expressed in multiple ovule tissues, suggesting that they may contribute to seed protein synthesis and accumulation. These findings provide new clues regarding the genetic basis of CPC and may accelerate the molecular breeding of cotton with ideal protein content.

## Introduction

Cottonseeds, which are the seeds of cotton plants, account for approximately 60% of the weight of seed cotton and are separated from seed cotton during processing. Cottonseeds contain a high level of both oil and protein, representing a crucial source for both edible oil and protein suitable for human consumption, as well as animal feed (Ji *et al.* 1985). The cottonseed cake and meal obtained after the extraction of oil typically comprise 30%–50% protein, indicative of their value as a protein resource (Gao *et al.* 1984). The quality of cottonseed protein is similar to that of bean protein, but its nutritional value is much higher than that of cereal protein; in terms of the amino acid contents, the essential amino acids (except for methionine) meet or exceed the standards recommended by the Food and Agriculture Organization of the United Nations and the World Health Organization (Zhao 2002). The cottonseed protein content (CPC), which is 3-times greater than the rice, corn, and wheat protein contents, is rich in various amino acids (especially lysine) required by humans (Liu *et al.* 2013). Therefore, fully exploiting seed protein resources can increase the economic value of cotton by-products, with financial benefits to cotton farmers, while also increasing the efficiency of cotton production.

The cottonseed protein content is a typical quantitative trait that is difficult to improve via traditional breeding methods. The use of molecular markers closely linked or significantly associated with quantitative trait loci (QTLs) for marker-assisted selection (MAS) can substantially increase breeding efficiency. Two principal methods for QTL identification, namely linkage mapping and association mapping, have been extensively applied to detect QTLs important cotton phenotypes, including yield and its components (Li *et al.* 2008, Wang *et al.* 2019), fibre quality (Li *et al.* 2018a, Shen *et al.* 2005), early maturity (Li *et al.* 2013a, 2018b), disease resistance (Mei *et al.* 2014, Zhao *et al.* 2014), plant architecture (Li *et al.* 2016, Song and Zhang 2009), and salt resistance (Saeed *et al.* 2014). Nevertheless, there are relatively few reports on the genetic mechanism underlying the CPC. On the basis of linkage mapping, Song and Zhang (2007) identified the QTL for CPC on chromosome 23 using a BC_1_S_1_ population derived from Hai7124 and TM-1. Yu *et al.* (2012) detected 22 QTLs for CPC on 12 chromosomes in an Upland cotton (*Gossypium hirsutum* L.) × *Gossypium barbadense* backcross inbred line population. Liu *et al.* (2013) generated 196 Upland cotton recombinant inbred lines (RILs) and detected four QTLs for CPC on chromosomes 4, 6, and 21. Liu *et al.* (2015) updated a high-density Upland cotton genetic map using RILs and identified 13 QTLs for CPC on 13 chromosomes. Wang *et al.* (2019) developed a RIL population from a cross between Upland cotton Yumian 1 and M11 and then mapped 12 QTLs for CPC on eight chromosomes. To date, there has been limited research conducted to identify QTL-associated markers for CPC in cotton via association mapping. Badigannavar and Myers (2015) detected five marker-trait associations for CPC based on amplified fragment length polymorphism markers. Du *et al.* (2018), Yuan *et al.* (2018) and Hu *et al.* (2022) identified 16, 17 and 27 significant single nucleotide polymorphisms (SNPs) associated with CPC through genome-wide association studies (GWASs), respectively.

High-throughput genotyping platforms have been widely used for GWAS-based research that has resulted in the identification of quantitative trait nucleotides (QTNs). However, in most previous GWAS-based investigations, only the allelic substitution effects were estimated, which are functions of additive and dominance effects and allelic frequency in random-mating populations. In these studies, the three genotypes (aa, Aa, AA) are typically coded as (0, 1, 2), (0, a, 2, 0 < a < 2), (–1, 0, 1), and (0, 0.5, 1) (Cho *et al.* 2010, Lippert *et al.* 2011, Segura *et al.* 2012, Wang *et al.* 2016, Wen *et al.* 2018, Zhou *et al.* 2013). These different coding strategies lead to different outcomes (Li *et al.* 2022). Furthermore, except for the multi-locus mixed model described by Segura *et al.* (2012), GWAS methods do not allow for the unbiased estimation of the additive and dominance effects of QTNs. Although several approaches and models have been used to detect QTN-by-environment interactions (QEIs) (Casale *et al.* 2017, Huang and Hu 2015, Korte *et al.* 2012, Moore *et al.* 2019, Robinson *et al.* 2017), the methods that were used are still imperfect for estimating additive and dominance effects or the effects of additive-by-environment and dominance-by-environment interactions. Epistasis is important for the genetic dissection of quantitative traits. Zhang *et al.* (2015a) and Wang *et al.* (2020) revealed QTN-by-QTN interactions (QQIs), but the polygenic backgrounds were either not detected or were relatively simple. Moreover, their methods are unfeasible because of the excessive number of markers as reported by Ning *et al.* (2018). Recently, Li *et al.* (2022) combined a three-variance component model with a multilocus random SNP-effect linear model, creating a novel methodological framework known as the 3VmrMLM, which stands for compressed and unified three-variance component mixed linear model. This framework and its associated methods (modules) can be used to detect all types of loci, with the effects of the identified loci estimated via GWAS without any biases. To date, 3VmrMLM has been widely applied for genetic analyses of various crops, including diploid rice (Zhao *et al.* 2023) and maize (Wen *et al.* 2023), allotetraploid *Brassica napus* (Han *et al.* 2022) and Upland cotton (Han *et al.* 2023), and hexaploid wheat (Wei *et al.* 2022).

In this study, to comprehensively dissect the genetic architecture of the CPC, several GWAS methods were used to detect QTNs, while 3VmrMLM methods were used to detect QEIs and QQIs in an association mapping panel comprising 152 Upland cotton accessions. In addition, the functions of the relevant candidate genes were predicted. The results may be useful for clarifying the complex genetic architecture of the CPC and promoting the molecular breeding of cotton with protein-rich seeds.

## Materials and Methods

### Experimental materials and field design

An assembly of 152 Upland cotton backbone cultivars and breeding lines, either cultivated within or imported to China, was chosen for the establishment of an association mapping panel. Of the accessions, 59 were from the Yellow River cotton-growing region, 25 were from the Yangtze River cotton-growing region, 38 were from northwestern China, 21 were from northern China, and 9 were from abroad (Supplemental Table 1). All accessions are theoretically homozygous and stable after multi-generation successive selfing. All these accessions were grown in northwestern China (Shihezi city, Xinjiang province) in 2019 (2019SHZ) and 2020 (2020SHZ) and in the Yellow River cotton-growing region (Yuncheng city, Shanxi province) in 2020 (2020YC). A randomized complete block design was used, with single-row plots and two repetitions. Due to differences in ecological environments, the sowing time in the Shihezi area is at the end of April, with a row length of 5 meters, each row containing 38 to 40 individual plants, and a row spacing of 0.45 meters; in the Yuncheng area, the sowing time is in mid-April, with a row length of 5 meters, each row containing 14 to 16 individual plants, and a row spacing of 1.0 meter. Standard local agricultural practices were applied throughout all tasks.

### Trait phenotyping and statistical analysis

During the peak boll-opening stage of cotton in each environment, 20 fully opened bolls in each row were harvested manually and ginned. The seeds were air-dried under natural conditions. Approximately 2 g cottonseed kernels per sample were crushed after removing the seed shell. The CPC was measured using the Foss Kjeltec^TM^ 8400 system (Sweden) according to Kjeldahl’s method with two technical replicates. The data were analyzed using SPSS17.0 software. The analysis of variance (ANOVA) and the estimation of broad-sense heritability (*H*^2^) for the trait were carried out using the AOV function of the QTL IciMapping software (version 3.2) (Wang *et al.* 2012).

### SNP genotyping

For each accession, genomic DNA was extracted from the tender leaves of randomly selected individual plants in each row, using the DNAsecure Plant kit produced by Tiangen Biotech (Beijing, China). The CottonSNP80K array containing 77,774 SNPs (Cai *et al.* 2017), which was developed on the basis of the TM-1 sequence (Zhang *et al.* 2015b) and the results of the resequencing of 100 Upland cotton cultivars (Fang *et al.* 2017), was used to genotype the 152 accessions. The analyses of image files were conducted with the GenomeStudio Genotyping Module (v1.9.4; Illumina). A previously established approach was employed for the genotyping of SNPs (Cai *et al.* 2017).

### Population structure and linkage disequilibrium analyses

Only SNPs with a minor allele frequency ≥0.05 and integrity ≥50% were used for analyzing the population structure and linkage disequilibrium (LD). The population structure was assessed using the ADMIXTURE software (Alexander *et al.* 2009). Pair-wise LD values for markers were calculated as the squared correlation coefficient (*R*^2^) of the alleles using the GAPIT program (Lipka *et al.* 2012).

### Genome-wide association study

Regarding the GWAS of CPC, the following six methods were used to detect QTNs: GEMMA-LM (Zhou and Stephens 2012, https://github.com/genetics-statistics/GEMMA), FaST-LMM (Lippert *et al.* 2011, https://github.com/fastlmm/FaST-LMM), EMMAX (Sul and Eskin 2013, https://csg.sph.umich.edu//kang/emmax/download/index.html), and three methods (modules) namely 3VmrMLM-Single_env, 3VmrMLM-Multi_env, and 3VmrMLM-Epistasis of 3VmrMLM (Li *et al.* 2022). Additionally, 3VmrMLM-Multi_env and 3VmrMLM-Epistasis were also used to detect QEIs and QQIs, respectively. Among six methods, the first three belong to the single-locus model, while the last three belong to the multi-locus model. All SNPs with a minor allele frequency ≥0.05 and integrity ≥50% were used for detecting QTNs and QEIs. Given the substantial computational resources required for QQI detection, the PLINK tool (Purcell *et al.* 2007) (https://www.cog-genomics.org/plink/2.0/) was first employed to filter all polymorphic markers (The command scripts can be found in Supplemental Data 1), and the remaining SNPs after filtering were then used for QQI detection. The identification of significant associations between markers and trait was achieved by utilizing an adjusted *P* value calculated as 1/*n* following the Bonferroni correction (Cai *et al.* 2017, Sun *et al.* 2017), where *n* represents the overall number of SNPs; the other parameters were set to default. For 3VmrMLM-Single_env, 3VmrMLM-Multi_env, and 3VmrMLM-Epistasis, all of the SNPs used to detect QTNs and QEIs and all of the marker pairs used to detect QQIs were first scanned individually to determine their *P* values, which were used to select significantly associated QTNs, QEIs, and QQIs (*P* ≤ 0.01). Next, all of the selected effects were incorporated into a multi-locus model to estimate the effects using an empirical Bayes method (Xu 2010). The non-zero effects were further identified by conducting likelihood ratio tests for true QTNs, QEIs, and QQIs. Significant QTNs, QEIs, and QQIs were determined after a Bonferroni correction with a *P* value of 0.05/*n* (*n* represents the number of SNPs), whereas suggested QTNs, QEIs, and QQIs were determined according to a logarithm of odds (LOD) ≥3.0 (Wang *et al.* 2016, Wen *et al.* 2018). Additionally, when estimating genetic effects using the 3VmrMLM method, the additive effects of the genotypes AA, Aa, and aa were coded as (1, 0, –1), and the dominance effects were coded as (0, 1, 0). Consequently, the coding values for the four types of interaction effects, additive-by-additive, additive-by-dominance, dominance-by-additive, and dominance-by-dominance, were derived by multiplying the corresponding additive and dominance effect coding values of the two loci. The specific steps for using the three methods of the 3VmrMLM, namely 3VmrMLM-Single_env, 3VmrMLM-Multi_env, and 3VmrMLM-Epistasis, to detect QTNs, QEIs, and QQIs can be found in the article by Li *et al.* (2022).

### Identification of candidate genes related to QTNs, and QTN-by-environment and QTN-by-QTN interactions

Because the CottonSNP80K array used in this study was developed using the Upland cotton TM-1 sequence (Zhang *et al.* 2015b), the TM-1 genome was used to mine the genes within the LD decay distance on either side of the detected SNPs of QTNs, QEIs, and QQIs. However, to exploit the latest information regarding the *G. hirsutum* genome (Hu *et al.* 2019), we converted the mined genes (beginning with *Gh*) to genes in the new genome (beginning with *GH*) (http://cotton.zju.edu.cn/download.html). To further identify potential candidate genes, all of the genes related to QTNs, QEIs, and QQIs were functionally annotated based on the Gene Ontology (GO), the Kyoto Encyclopedia of Genes and Genomes (KEGG), and The Arabidopsis Information Resource (TAIR) databases. For the genes related to QTNs and QEIs, a functional enrichment analysis was performed using OmicStudio tools (Lyu *et al.* 2023, https://www.omicstudio.cn/tool). Fisher’s exact test (*P* < 0.05) was used to determine the significance of the enrichment of GO terms and KEGG pathways. Genes annotated with significantly enriched GO terms or KEGG pathways were considered to be candidate genes. Because QQI essentially reflects the interaction between genes (genetics) and the interaction between proteins (molecular biology), for the QQI-related genes, their Swiss-Prot IDs were retrieved and then protein–protein interactions were predicted using STRING (https://cn.string-db.org/), with the Arabidopsis protein database used as the protein library.

## Results

### Phenotypic data analysis

**Fig. 1.**
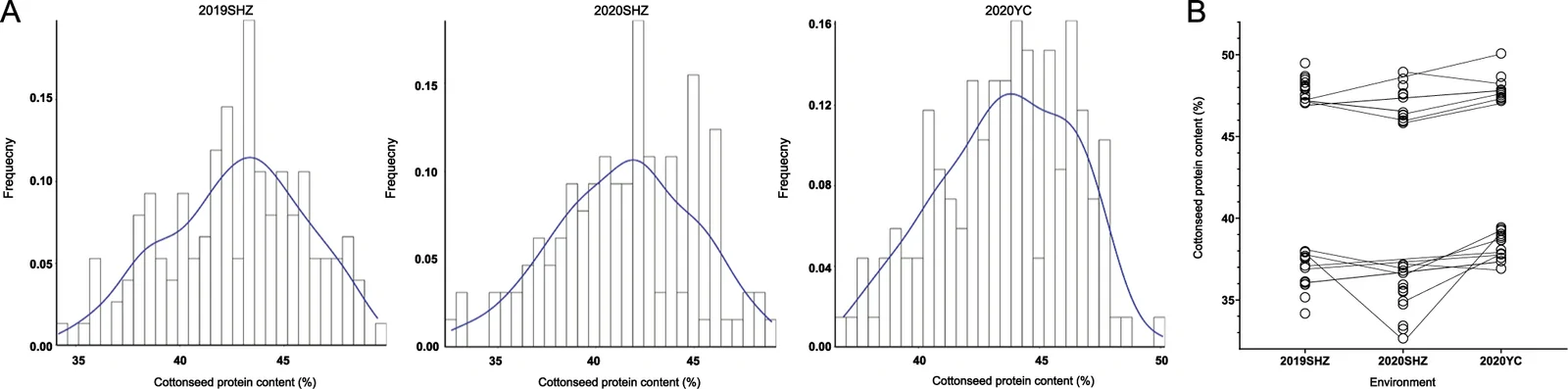
Frequency of cottonseed protein content (CPC) in three environments (A) and connect-the-dots diagram of accessions with the top 10% (upper panel) and bottom 10% (lower panel) CPC across three environments (B).

The phenotypes of 152 Upland cotton accessions were analyzed (Table 1, Fig. 1A). There was extensive continuous variation in the CPC in the three environments, with the largest coefficient of variation in 2020SHZ (8.53%), followed by 2019SHZ (7.79%) and 2020YC (6.45%). The variations caused by the genotype (G), environment (E), and genotype-by-environment interaction (G × E) were highly significant (*P* < 0.001); the average heritability of the CPC in the three environments was 75.57% (Table 1). The results of the correlation analysis show that the correlation coefficients of the CPC among the three environments all reached the highly significant level (Supplemental Table 2). Additionally, several materials were found to be repeated in two or three environments among both the top 10% and bottom 10% in terms of CPC (Fig. 1B). These indicated that although the trait is influenced by the environment, there are stable QTLs underlying its genetic control. Accordingly, the CPC exhibited the typical characteristics of quantitative traits, thereby necessitating additional genetic analyses.

### SNP distribution, population structure, and linkage disequilibrium

**Fig. 2.**
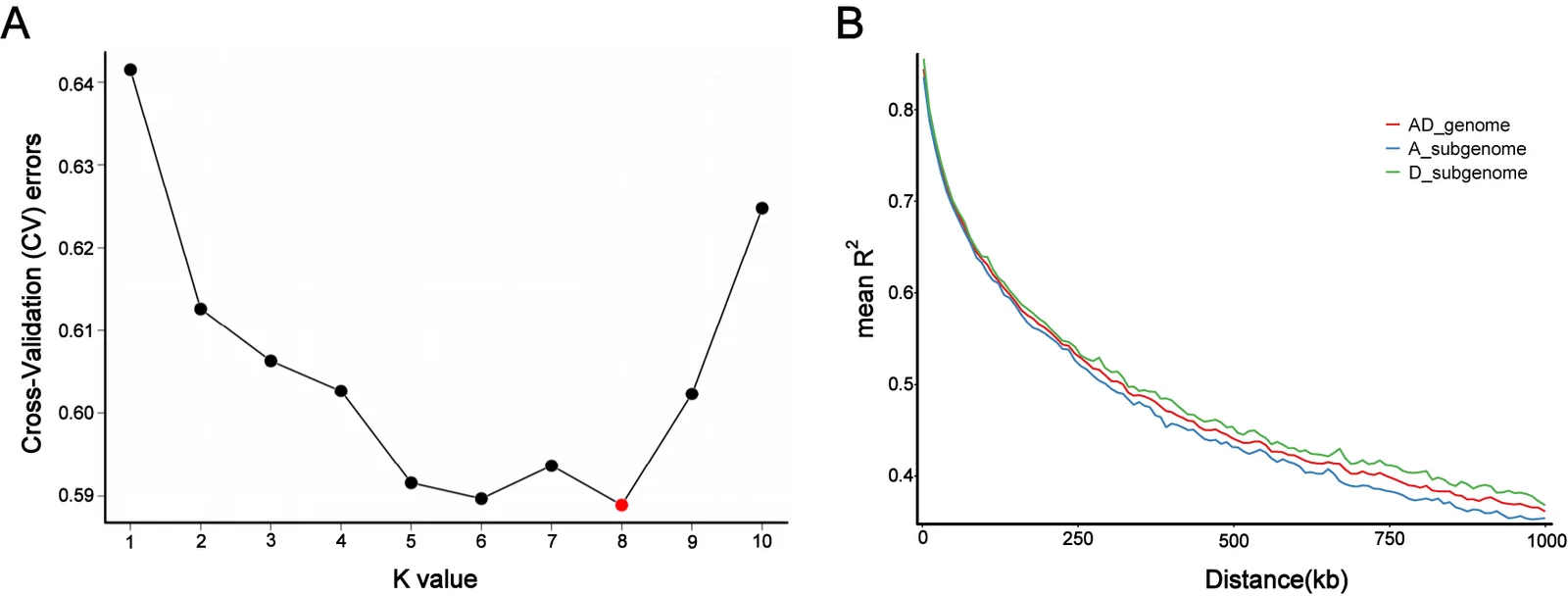
Population structure (A) and linkage disequilibrium decay (B) of Upland cotton accessions.

The genotypes of all accessions were examined using the CottonSNP80K array. Of the 77,774 SNPs in the array, 49,533 were detected as high-quality SNPs with a minor allele frequency ≥0.05 and integrity ≥50% in the population. These SNPs were not evenly distributed across the *G. hirsutum* genome, with 25,820 and 23,830 SNPs in the A and D subgenomes, respectively (Supplemental Table 3). The population structure estimation showed that the lowest cross-validation error was observed when *K* = 8 (Fig. 2A), which was thus determined to be the optimum *K* value (*K* is the number of subpopulations set when estimating population structure). Hence, the accessions were separated into eight subpopulations. The LD analysis indicated the average LD decay distance in the AD genome of our population was approximately 550 kb (*R*^2^ = 0.42 at half the maximum value), which was longer and shorter than the corresponding distances in the A and D subgenomes, respectively (Fig. 2B).

### QTN detection for cottonseed protein content

**Fig. 3.**
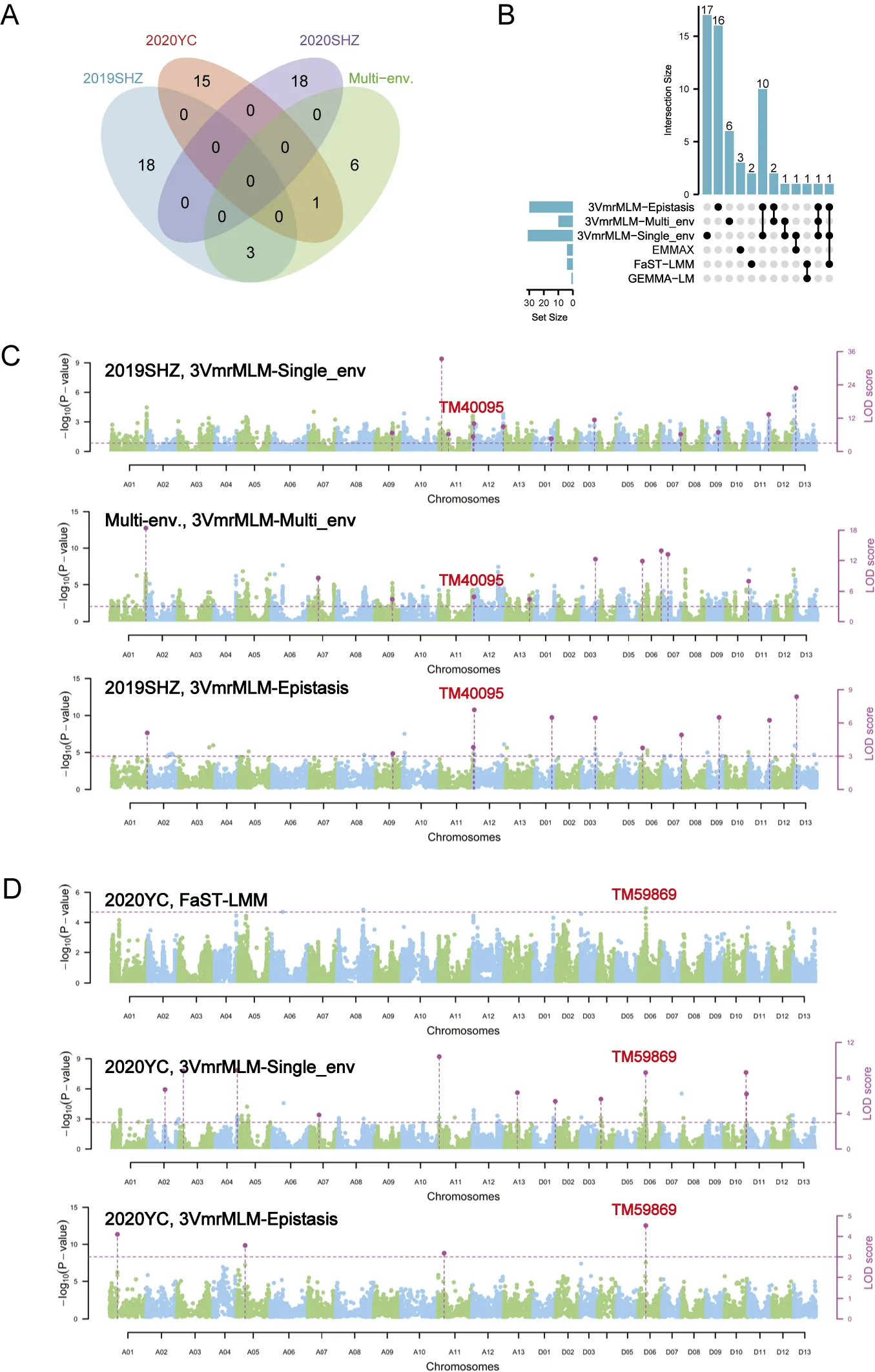
(A) Venn diagram of common QTNs among the four environments (datasets). (B) UpSet plot of common QTNs among the six methods. (C) Manhattan plot for the QTN TM40095 detected by three methods. (D) Manhattan plot for the QTN TM59869 detected by three methods. In figures (C) and (D), for the result of FaST-LMM, the y-axis shows the –log_10_*P* values obtained from a genome-wide scan of all markers, with the threshold line set at –log_10_*P* = 4.69 (*P* = 1/49533); for the results of 3VmrMLM-Single_env, 3VmrMLM-Multi_env, and 3VmrMLM-Epistasis, the left y-axis shows the –log_10_*P* values obtained from the genome-wide scan of all markers in the first step of 3VmrMLM, while the right y-axis shows the LOD values of the QTNs obtained through the likelihood ratio test in the second step of 3VmrMLM, with the threshold line set at LOD = 3.0.

Of the six methods used for detecting QTNs (GEMMA-LM, FaST-LMM, EMMAX, 3VmrMLM-Single_env, 3VmrMLM-Multi_env, and 3VmrMLM-Epistasis), the first five were based on the data for 49,533 SNPs (detailed SNP data are not presented), whereas the sixth was based on the data for 10,104 SNPs after the filtering using PLINK (detailed SNP data are not presented). Ultimately, GEMMA-LM, FaST-LMM, EMMAX, 3VmrMLM-Single_env, 3VmrMLM-Multi_env, and 3VmrMLM-Epistasis detected 1, 4, 4, 31, 10, and 30 QTNs for CPC, respectively, in the datasets of the three environments and multi-environment. These QTNs were located on 24 chromosomes (the exceptions were A10 and D12), with phenotypic variation explained (PVE) values of 0.29%–14.59% (Supplemental Table 4). Moreover, 4 QTNs were detected in two datasets (environments) simultaneously (Fig. 3A), and 15 QTNs were detected by two methods simultaneously and 2 QTNs were detected by three methods simultaneously (Fig. 3B).

To identify stable QTNs applicable for breeding, the QTNs detected by three methods simultaneously were analyzed in more detail. The QTN TM40095 on A12 was simultaneously detected by 3VmrMLM-Single_env, 3VmrMLM-Multi_env, and 3VmrMLM-Epistasis, with additive effects of 0.53–0.98, dominance effects of –0.22–0.29, and PVE values of 2.15%–7.13% (Table 2, Fig. 3C). The QTN TM59869 on D06 was simultaneously detected by FaST-LMM, 3VmrMLM-Single_env, and 3VmrMLM-Epistasis, with additive effects of 0.77–0.86, but no dominance effect, and PVE values of 6.84%–9.76% (Table 2, Fig. 3D). Both QTNs were identified as significant QTNs by two of the three methods. The analysis of phenotypic differences between different alleles showed that the elite alleles for TM40095 were A in all three methods, and for TM59869 were C in all three methods. Both elite alleles could increase CPC (Table 2).

### QTN-by-environment interaction detection for cottonseed protein content

The multi-environment dataset was analyzed using 3VmrMLM-Multi_env to detect QEIs. Five QEIs (one significant and four suggested) related to CPC were detected (Table 3, Supplemental Fig. 1). The corresponding loci were on chromosomes A01, A05, A06, and D13, with a PVE of 1.61%–3.13%. The five QEIs had A × E (additive-by-environment interaction) effects, but no D × E (dominance-by-environment interaction) effects. To simplify the table, the column for D × E was not shown in Table 3. For example, the loci TM273 and TM9997 had additive-by-environment interaction effects of –0.44–0.83 and –0.64–0.66, respectively. There was some inconsistency in the direction of the additive-by-environment interaction effects of the five QEIs across environments. Considered together, these results reflect the complexity of the interaction between a quantitative trait-based genotype and the environment.

### QTN-by-QTN interaction detection for cottonseed protein content

Using 3VmrMLM-Epistasis and the 10,104 SNPs remaining after the filtering by PLINK tool, 29 QQIs associated with CPC (4 significant and 25 suggested) with additive-by-additive, additive-by-dominance, dominance-by-additive, and/or dominance-by-dominance effects were detected across three environments (Table 4, Supplemental Fig. 2). For example, 11 QQIs for CPC were detected in 2019SHZ. The interaction between TM20 on chromosome A01 and TM14246 on chromosome A06 had an additive-by-additive effect of –0.32, whereas the interaction between TM10542 on chromosome A05 and TM59085 on chromosome D06 had an additive-by-additive effect of –0.03 and an additive-by-dominance effect of 0.57. The interaction between TM10547 on chromosome A05 and TM41538 on chromosome A12 had an additive-by-additive effect of –0.14 and a dominance-by-additive effect of –0.63, while the interaction between TM13610 on chromosome A06 and TM53119 on chromosome D02 had an additive-by-additive effect of –0.17, an additive-by-dominance effect of –0.72, a dominance-by-additive effect of 0.20, and a dominance-by-dominance effect of 0.21. The 29 QQIs involved almost all Upland cotton chromosomes, with a PVE of 0.01%–13.07%.

### Identification of candidate genes related to cottonseed protein content

Considering the LD decay distance of the population used in this study, the 550-kb regions flanking the detected SNPs of QTNs, QEIs, and QQIs were screened for possible candidate genes. The identified candidate genes (beginning with *Gh*) were converted to genes in the new genome (beginning with *GH*). A total of 49 candidate genes were identified flanking the SNPs of the above-mentioned two stable QTNs. Additionally, 174 candidate genes were detected flanking the SNPs of five QEIs, whereas 269 candidate genes were detected flanking the SNPs of four significant QQIs. Using the GO, KEGG, and TAIR databases, the candidate genes related to the above-mentioned loci were functionally annotated (Supplemental Table 5).

The significantly enriched GO terms and KEGG pathways of the genes linked to QTNs and QEIs were determined. Because the QTN or QEI loci were associated with the same trait, the candidate genes were expected to be annotated with the same GO terms or KEGG pathways. Therefore, significantly enriched (*P* < 0.05) GO terms and KEGG pathways for genes in at least two loci are listed in Supplemental Table 6. The 49 candidate genes related to the two stable QTNs included 10 non-redundant genes annotated with four significantly enriched GO terms (e.g., vacuolar membrane, transmembrane receptor protein serine/threonine kinase activity, cell surface receptor signaling pathway, and oxidation-reduction process) and two non-redundant genes annotated with one significantly enriched KEGG pathway (tryptophan metabolism). The 174 candidate genes related to five QEIs included 44 non-redundant genes annotated with 15 significantly enriched GO terms (e.g., cilium, telomere maintenance, and protein-arginine omega-N asymmetric methyltransferase activity) and four non-redundant genes annotated with one significantly enriched KEGG pathway (aminoacyl-tRNA biosynthesis).

**Fig. 4.**
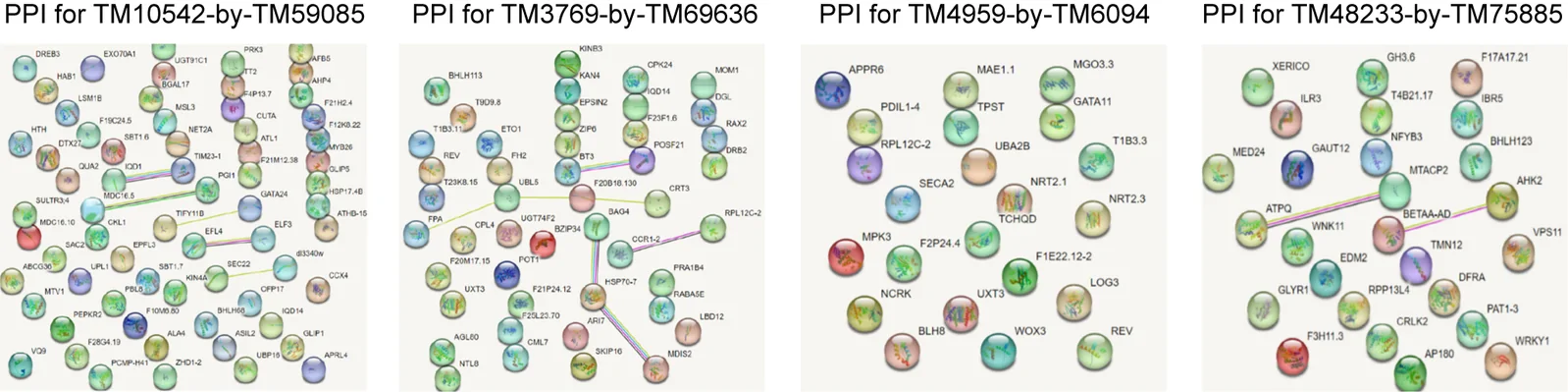
Protein–protein interaction prediction for four significant QTN-by-QTN interactions (QQIs) related to the cottonseed protein content.

Interactions between the proteins encoded by the 269 candidate genes related to four significant QQIs were predicted. The following eight protein–protein interactions involving three QQIs and eight gene pairs were detected: MDC16.5 (*GH_A05G1385*)–PGI1 (*GH_D06G0320*), TIFY11B (*GH_A05G1405*)–GATA24 (*GH_D06G0301*), and SEC22 (*GH_A05G1402*)–dl3340w (*GH_D06G0329*) for the TM10542-by-TM59085 QQI; FPA (*GH_A02G0124*)–UBL5 (*GH_D08G2269*), F20B18.130 (*GH_A02G0128*)–UBL5 (*GH_D08G2269*), HSP70-7 (*GH_A02G0076*)–BAG4 (*GH_D08G2298*), and HSP70-7 (*GH_A02G0076*)–MDIS2 (*GH_D08G2303*) for the TM3769-by-TM69636 QQI; and ATPQ (*GH_D11G1591*)–MTACP2 (*GH_D01G0704*/*GH_D01G0705*) for the TM48233-by-TM75885 QQI (Fig. 4). Specific details regarding these interactions, including full protein names, are listed in Supplemental Table 7. Notably, some protein–protein interactions (e.g., IQD1–TIM23-1 and EFL4–ELF3 for the TM10542-by-TM59085 QQI) were not between two SNPs of the same QQI. These interactions were not considered.

## Discussion

Cottonseeds are a source of nutritionally valuable proteins. In cotton, many marker–trait associations have been identified through various association analyses. However, these traits were mainly yield and its components (Wang *et al.* 2019), fibre quality (Li *et al.* 2018a), early maturity (Li *et al.* 2018b), disease resistance (Zhao *et al.* 2014), plant architecture (Li *et al.* 2016), and salt resistance (Saeed *et al.* 2014). There are few reports regarding association analysis of CPC (Badigannavar and Myers 2015, Du *et al.* 2018, Hu *et al.* 2022, Yuan *et al.* 2018). Therefore, we used multiple GWAS methods to dissect the genetic architecture of this important trait in depth.

A suitable panel for an association analysis should include as much genetic variation as can be reliably analyzed in common environments (Flint-Garcia *et al.* 2005). It is especially important to select as many genetically diverse samples as possible. In this study, 152 Upland cotton backbone cultivars and breeding lines from various cotton-growing areas in China and elsewhere were analyzed. Although the number of materials used in this study is not very rich, these accessions were derived from multiple pedigrees, including Deltapine, Stoneville, Foster, and Uganda. The phenotypic evaluation revealed considerable phenotypic differences and the relatively high heritability of the CPC. Additionally, 49,533 high-quality SNPs were obtained (i.e., 63.69% of the 77,774 SNPs in the CottonSNP80K array), indicative of substantial genetic diversity. Hence, the association panel was appropriate for the GWAS of the target trait. Moreover, in order to improve the detection efficacy of associated loci, multiple GWAS methods were used simultaneously to obtain reliable results.

In a complete mixed model framework, there should be 5, 10, and 15 variance components for the detection of QTNs, QEIs, and QQIs, respectively, if the SNP effects are treated as random effects (Li *et al.* 2022). However, because 3VmrMLM has only three variance components for the detection of QTNs, QEIs, and QQIs, it represents a compressed variance component mixed model applicable for a GWAS. In particular, all the effects that need to be estimated are consolidated into a singular vector associated with effects, the polygenic influences are unified into a single polygenic representation (Li *et al.* 2022). Most of the existing GWAS methods have been used to detect and estimate the substitution effects of alleles. Segura *et al.* (2012) used a mixed model to detect and estimate the additive effects, controlling for the polygenic background. However, their model did not explicitly include dominance effects or additive-by-dominance interactions, focusing primarily on additive genetic variance and residuals. In a recent study conducted by Wang *et al.* (2020) to detect QQIs, although the additive, dominance, additive-by-additive, additive-by-dominance, and dominance-by-dominance effects were estimated, the method was too simple to effectively control the polygenic background. In addition, compared with 3VmrMLM, there were more variance components. Unlike previous GWAS methods, 3VmrMLM can correctly detect all types of loci and estimate their effects with almost no biases (Li *et al.* 2022). In two recent studies on Upland cotton, 3VmrMLM was used to detect 171 QTNs and 42 QQIs for three fibre quality traits (Han *et al.* 2023) as well as 288 QTNs, 14 QEIs, and 21 QQIs for three early-maturity traits (Li *et al.* 2024). These results reflect the applicability of the 3VmrMLM framework for analyzing allotetraploids. In the current study, significantly more QTNs were detected by the 3VmrMLM methods (3VmrMLM-Single_env, 3VmrMLM-Multi_env, and 3VmrMLM-Epistasis) than by the other three methods (Supplemental Table 4). In addition, five QEIs with a PVE of 1.61%–3.13% were detected by 3VmrMLM-Multi_env, whereas 29 QQIs with a PVE of 0.11%–13.07% were detected by 3VmrMLM-Epistasis. More importantly, many small-effect loci (PVE <1%) for CPC were detected, with 3VmrMLM-Epistasis detecting nearly half of the small-effect QQI loci. These QEIs and QQIs partly address the problem of missing heritability. Although many suggested loci were detected by 3VmrMLM, they were complementary to significant loci. Overall, the 3VmrMLM framework and its methods are suitable for the GWAS-based detection of various types of loci.

Obtaining stable QTLs/QTNs for target traits is a prerequisite for MAS. Loci detected simultaneously in multiple populations, environments, or using different methods tend to be more stable. (Jia *et al.* 2011, Li *et al.* 2018b, Su *et al.* 2010). In the present study, six GWAS methods were used to detect QTNs for CPC. The 17 QTNs detected by at least two methods were highly stable. Notably, TM40095 on A12 and TM59869 on D06, which were detected by three methods, explained 2.15%–7.13% and 6.84%–9.76% of the phenotypic variation, respectively. These two QTNs are relatively stable and likely suitable for the MAS of CPC. We also compared the detected QTNs with marker loci related to CPC identified in previous studies according to their physical locations in the TM-1 genome (Zhang *et al.* 2015b). On the basis of the average LD decay distance in our population, the loci that were within the same 550-kb region were considered to be the same. Thus, three of these QTNs overlapped previously reported marker loci (Supplemental Table 8). Specifically, TM11103 corresponded to BNL3992 on A05 (Yu *et al.* 2012), TM63795 corresponded to BNL2734 on D07 (Yu *et al.* 2012), and TM71935 corresponded to BNL1672/BNL3031 on D09 (Song and Zhang 2007). It should be mentioned that the two stable QTNs detected in this study were not detected in previous studies, possibly because of differences in germplasm populations. To summarize, the above-mentioned five CPC-related QTNs detected simultaneously using multiple methods and/or in different populations with different genetic backgrounds may be stable and useful for MAS. The remaining QTNs did not correspond to any reported loci, suggestive of their novelty. Therefore, it is essential to conduct further validations across various populations, environment, and/or mapping methods.

Bioinformatics methods (e.g., functional annotation, enrichment analysis, and protein–protein interaction analysis) are widely used to mine for candidate genes underlying target traits. In this study, 492 candidate genes related to the stable QTNs, QEIs, and significant QQIs were functionally annotated using GO, KEGG, and TAIR databases (Supplemental Table 5). Subsequent analyses revealed four GO terms and one KEGG pathway significantly enriched among 10 and two non-redundant genes related to stable QTNs, respectively, as well as 15 GO terms and one KEGG pathway significantly enriched among 44 and four non-redundant genes related to QEIs, respectively (Supplemental Table 6). Moreover, eight protein–protein interactions involving three QQIs and eight gene pairs were predicted using STRING (Supplemental Table 7). These findings may be useful for further validating potential candidate genes related to the CPC. We conducted a literature search to clarify the biological functions and associated metabolic pathways of the above-mentioned candidate genes and their homologs, but none of the genes were directly related to protein synthesis or accumulation. Hence, we obtained Upland cotton TM-1 RNA-seq data for 75 genes (12 for QTNs and 48 for QEIs based on functional enrichment as well as 15 for QQIs based on protein interactions) for further analyses (http://cotton.zju.edu.cn/10.rnasearch.html) (Supplemental Table 9). The data were derived from 18 cotton samples at different developmental stages, including vegetative growth and reproductive growth. Four of the 75 genes, including *GH_D06G1049* related to QTNs and *GH_A06G1663*, *GH_D13G0601*, and *GH_A05G0236* related to QEIs, were preferentially expressed in at least four of the six ovule tissues in all 18 samples (top four) (Supplemental Table 9). In particular, *GH_D06G1049* was more highly expressed in all six ovule tissues than in the other tissues. Considering seeds develop from the ovule in the ovary, these four genes may influence seed protein synthesis and accumulation. Because protein anabolism is a complex physiological and biochemical process, the potential contributions of all of the above-mentioned genes to the CPC will need to be verified experimentally (e.g., CRISPR/Cas9-mediated gene knockout, gene overexpression, multi-environment phenotypic analysis, two-dimensional gel electrophoresis, and yeast two-hybrid assay).

With the continuous improvement of QTL mapping information, various attempts have been made to use molecular markers associated with QTLs to improve breeding efficiency, such as marker-assisted selection (Li *et al.* 2013b), genomic selection (GS) (Meuwissen *et al.* 2001), and molecular design (Wang *et al.* 2007). However, in general, the number of loci controlling quantitative traits is large and their effects are small, making it difficult to directly apply them in breeding practice (Heffner *et al.* 2009); GS can significantly elevate genetic gains per unit of time within crop-breeding programs, but to increase the efficiency of GS, it is necessary to consider all relevant factors when developing genomic prediction methodologies to improve prediction accuracy (Alemu *et al.* 2024). Molecular design has been increasingly applied in crop genetic improvement in recent years (Li *et al.* 2010). By integrating different technologies, various factors in breeding programs can be effectively simulated, screened, and optimized to enhance breeding efficiency. Additionally, the best strategies for selecting parents and the offspring have been proposed to facilitate the transition from traditional “experience-based breeding” to “genome selection breeding” (Peleman and Voort 2003). Currently, a breeding platform based on genomic selection for rice has been developed and applied (Qiu *et al.* 2018, Xu *et al.* 2021). Although there are fewer established functional markers/genes in the cotton genome compared to the rice genome, GS in cotton does not rely on the knowledge of functional markers or genes. Instead, it relies on genome-wide DNA markers. The CottonSNP80K array provides a valuable tool for this purpose. However, the main bottleneck in implementing GS in cotton breeding is the collection of phenotype data from a large number of germplasms. In contrast, molecular design breeding can be performed by using the limited number of functional markers and the associated genetic information. Recently, Zhang *et al.* (2022) developed a genetic breeding simulation and prediction platform named Blib, whose application module ISB can simulate the breeding processes or methods for pure-line varieties, hybrid varieties, and clonal varieties (Li *et al.* 2025). In this study, various types of loci and their effects on CPC were determined via GWAS. Further work will utilize the elite alleles of stable QTNs to identify several high-protein content parental germplasms. Subsequently, based on the various types of loci and their effects identified in this study, three input files will be constructed using the ISB module: “genetic model” (including environment, traits, chromosomes and loci, marker or gene loci, alleles, genetic effects, interaction networks, etc.), parents of the starting breeding population (including allele frequencies at each locus or the allelic composition of each parent at each locus), and simulated breeding processes or methods (including the number of replications in the simulated experiment, the number of breeding processes, the number of breeding cycles, the number of hybrid combinations and mating types, etc.). Through breeding simulation, breakthroughs in cotton molecular design breeding will be achieved, and valuable references will also be provided for related research in other crops.

## Author Contribution Statement

NA wrote the manuscript. HZ, GF, LD, BL, and HL performed field experiments and trait phenotyping analyses. ZZ, NW, and XT performed SNP genotyping experiments. HG and KN analyzed data. CL conceived and designed experiments.

## Supplementary Material

Supplemental Data

Supplemental Figures

Supplemental Tables

## Figures and Tables

**Table 1.** Statistical analysis of the cottonseed protein content

Env.	Range (%)	Mean (%) ± SD	CV (%)	G (F-value)	E (F-value)	G × E (F-value)	*H*^2^ (%)
2019SHZ	34.17–49.49	42.68 ± 3.33	7.79	146.57***	1207.41***	47.05***	75.57
2020SHZ	32.65–48.95	41.46 ± 3.54	8.53				
2020YC	36.91–50.08	43.61 ± 2.81	6.45				

G: genotype; E: environment; G × E: genotype-by-environment interaction; ***: *P* < 0.001; *H*^2^ (%): broad-sense heritability.

**Table 2.** Stable quantitative trait nucleotides (QTNs) for the cottonseed protein content detected using three GWAS methods

SNP	Chr.	Posi. (bp)	Method	Env.	LOD	A	D	PVE (%)	–log_10_*P*	Sig.	Elite allele
TM40095	A12	2063413	3VmrMLM-Single_env	2019SHZ	10.05	0.98	−0.16	7.13	10.05	SIG	A
			3VmrMLM-Multi_env	Multi-env.	4.92	0.53	0.29	2.15	4.92	SUG	A
			3VmrMLM-Epistasis	2019SHZ	7.18	0.72	−0.22	4.26	7.18	SIG	A
TM59869	D06	20608253	FaST-LMM	2020YC				9.76	4.94	SIG	C
			3VmrMLM-Single_env	2020YC	8.60	0.77		6.84	9.50	SIG	C
			3VmrMLM-Epistasis	2020YC	4.53	0.86		8.37	5.30	SUG	C

LOD: logarithm of the odds; A: additive effect; D: dominance effect; PVE (%): phenotypic variation explained; Sig.: significance level; SIG: significant; SUG: suggested.

**Table 3.** QTN-by-environment interactions (QEIs) for the cottonseed protein content detected by 3VmrMLM-Multi_env

SNP	Chr.	Posi. (bp)	LOD	A × E1	A × E2	A × E3	PVE (%)	–log_10_*P*	Sig.
TM273	A01	5351569	6.20	−0.39	−0.44	0.83	3.13	6.20	SIG
TM9997	A05	1637303	5.11	0.66	−0.02	−0.64	2.58	5.11	SUG
TM13308	A05	87093725	5.49	−0.65	−0.01	0.66	2.58	5.49	SUG
TM17169	A06	88949157	4.26	−0.68	0.28	0.40	2.11	4.26	SUG
TM80413	D13	7704380	3.41	−0.55	0.09	0.46	1.61	3.41	SUG

A × E1, A × E2, and A × E3 are the additive effects specific to 2019SHZ, 2020SHZ, and 2020YC, respectively; PVE (%): phenotypic variation explained; Sig.: significance level; SIG: significant; SUG: suggested.

**Table 4.** QTN-by-QTN interactions (QQIs) for the cottonseed protein content detected by 3VmrMLM-Epistasis

Env.	SNP (1)	Chr. (1)	Posi. (1) (bp)	SNP (2)	Chr. (2)	Posi. (2) (bp)	LOD	A × A	A × D	D × A	D × D	PVE (%)	–log_10_*P*	Sig.
2019SHZ	TM20	A01	875363	TM14246	A06	21962156	4.35	−0.32				0.69	5.12	SUG
	TM9071	A04	10592604	TM50134	D01	59324179	4.44	−0.08	0.10	0.60	0.19	2.16	3.39	SUG
	TM9987	A05	1560769	TM55315	D03	44926173	5.53	−0.43				1.39	6.35	SUG
	TM10542	A05	11394965	TM59085	D06	2960372	12.08	−0.03	0.57			2.80	12.08	SIG
	TM10547	A05	11449039	TM41538	A12	46861543	3.35	−0.14		−0.63		2.70	3.37	SUG
	TM13610	A06	1293713	TM53119	D02	63047297	7.13	−0.17	−0.72	0.20	0.21	0.79	5.89	SUG
	TM14554	A06	34572850	TM72474	D09	41893754	4.58	0.22	−0.38			2.06	4.58	SUG
	TM19243	A07	14863330	TM56550	D04	48603853	4.61	−0.66				3.84	5.39	SUG
	TM29385	A08	90255032	TM37858	A11	20859202	6.09	−0.25	0.03			0.51	6.09	SUG
	TM29993	A09	1604468	TM55634	D04	4704700	4.89	−0.26	0.47			2.60	4.89	SUG
	TM41795	A12	57144032	TM54999	D03	38354118	3.20	−0.07	−0.17	0.04	−0.09	0.06	2.28	SUG
2020SHZ	TM3769	A02	1044249	TM69636	D08	60622174	8.65	−0.05				0.01	9.55	SIG
	TM4717	A02	41385385	TM75140	D10	61324931	5.30	−0.14	0.02			0.13	5.30	SUG
	TM4959	A02	55806027	TM6094	A03	4553074	9.27	−0.10		0.36		1.25	9.27	SIG
	TM5065	A02	67093779	TM19093	A07	13432870	5.59	0.28	0.40			0.85	5.59	SUG
	TM11361	A05	28752771	TM66562	D07	49697810	4.22	−0.13	−0.64			2.92	4.22	SUG
	TM13708	A06	3255855	TM36340	A10	91215767	7.77	−0.32		−0.1		0.52	7.77	SUG
	TM32801	A09	53134429	TM57244	D05	12043687	7.2	0.13	0.26	0.29	0.16	0.47	5.95	SUG
	TM47121	A13	68590429	TM66821	D08	264184	4.53	−0.07	0.29			0.80	4.53	SUG
	TM48233	D01	7587675	TM75885	D11	15484974	12.51	0.01	0.37			0.84	12.51	SIG
	TM52455	D02	55561566	TM80606	D13	18800035	8.62	0.60	0.30	0.25	0.24	1.47	7.30	SUG
	TM57407	D05	18249724	TM64004	D07	17224312	6.18	0.43	0.09			0.92	6.18	SUG
2020YC	TM935	A01	18871381	TM29469	A08	92545327	4.82	−0.40	−0.03	−0.03	0.32	2.23	3.74	SUG
	TM938	A01	18956573	TM41305	A12	36355025	4.28	−0.58		0.78		13.07	4.28	SUG
	TM5573	A02	79288246	TM19653	A07	23618377	5.12	−0.69	0.21			5.93	5.12	SUG
	TM11103	A05	23962836	TM39729	A11	90257854	3.89	−0.21				0.53	4.64	SUG
	TM12876	A05	78504237	TM76132	D11	22087150	4.70	0.55				3.76	5.48	SUG
	TM21959	A08	4647193	TM73439	D10	5505054	3.60	0.34	−0.36			3.49	3.60	SUG
	TM43738	A13	14056285	TM56002	D04	16231805	5.37	−0.75		−0.17		5.27	5.37	SUG

A × A: additive-by-additive; A × D: additive-by-dominance; D × A: dominance-by-additive; D × D: dominance-by-dominance; PVE (%): phenotypic variation explained; Sig.: significance level; SIG: significant; SUG: suggested.

## References

[B1] Alemu, A., J. Åstrand, O.A. Montesinos-López, J. Isidro y Sánchez, J. Fernández-Gónzalez, W. Tadesse, R.R. Vetukuri, A.S. Carlsson, A. Ceplitis, J. Crossa et al. (2024) Genomic selection in plant breeding: Key factors shaping two decades of progress. Mol Plant 17: 552–578. 38475993 10.1016/j.molp.2024.03.007

[B2] Alexander, D.H., J. Novembre and K. Lange (2009) Fast model-based estimation of ancestry in unrelated individuals. Genome Res 19: 1655–1664. 19648217 10.1101/gr.094052.109 PMC2752134

[B3] Badigannavar, A. and G.O. Myers (2015) Genetic diversity, population structure and marker trait associations for seed quality traits in cotton (*Gossypium hirsutum*). J Genet 94: 87–94. 25846880 10.1007/s12041-015-0489-x

[B4] Cai, C., G. Zhu, T. Zhang and W. Guo (2017) High-density 80 K SNP array is a powerful tool for genotyping *G. hirsutum* accessions and genome analysis. BMC Genomics 18: 654. 28835210 10.1186/s12864-017-4062-2 PMC5569476

[B5] Casale, F.P., D. Horta, B. Rakitsch and O. Stegle (2017) Joint genetic analysis using variant sets reveals polygenic gene-context interactions. PLoS Genet 13: e1006693. 28426829 10.1371/journal.pgen.1006693 PMC5398484

[B6] Cho, S., K. Kim, Y.J. Kim, J.K. Lee, Y.S. Cho, J.Y. Lee, B.G. Han, H. Kim, J. Ott and T. Park (2010) Joint identification of multiple genetic variants via elastic-net variable selection in a genome-wide association analysis. Ann Hum Genet 74: 416–428. 20642809 10.1111/j.1469-1809.2010.00597.x

[B7] Du, X., S. Liu, J. Sun, G. Zhang, Y. Jia, Z. Pan, H. Xiang, S. He, Q. Xia, S. Xiao et al. (2018) Dissection of complicate genetic architecture and breeding perspective of cottonseed traits by genome-wide association study. BMC Genomics 19: 451. 29895260 10.1186/s12864-018-4837-0 PMC5998501

[B8] Fang, L., H. Gong, Y. Hu, C. Liu, B. Zhou, T. Huang, Y. Wang, S. Chen, D.D. Fang, X. Du et al. (2017) Genomic insights into divergence and dual domestication of cultivated allotetraploid cottons. Genome Biol 18: 33. 28219438 10.1186/s13059-017-1167-5 PMC5317056

[B9] Flint-Garcia, S.A., A.C. Thuillet, J. Yu, G. Pressoir, S.M. Romero, S.E. Mitchell, J. Doebley, S. Kresovich, M.M. Goodman and E.S. Buckler (2005) Maize association population: A high-resolution platform for quantitative trait locus dissection. Plant J 44: 1054–1064. 16359397 10.1111/j.1365-313X.2005.02591.x

[B10] Gao, Z.C., J.F. Li and Y.X. Jiang (1984) Glandless cottonseeds are high quality protein sources. China Cotton 39: 19–20 (in Chinese).

[B11] Han, X., Q. Tang, L. Xu, Z. Guan, J. Tu, B. Yi, K. Liu, X. Yao, S. Lu and L. Guo (2022) Genome-wide detection of genotype environment interactions for flowering time in *Brassica napus*. Front Plant Sci 13: 1065766. 36479520 10.3389/fpls.2022.1065766 PMC9721451

[B12] Han, Z., H. Ke, X. Li, R. Peng, D. Zhai, Y. Xu, L. Wu, W. Wang and Y. Cui (2023) Detection of epistasis interaction loci for fiber quality-related trait via 3VmrMLM in upland cotton. Front Plant Sci 14: 1250161. 37841603 10.3389/fpls.2023.1250161 PMC10568130

[B13] Heffner, E.L., M.E. Sorrells and J.L. Jannink (2009) Genomic selection for crop improvement. Crop Sci 49: 1–12.

[B14] Hu, Y., J. Chen, L. Fang, Z. Zhang, W. Ma, Y. Niu, L. Ju, J. Deng, T. Zhao, J. Lian et al. (2019) *Gossypium barbadense* and *Gossypium hirsutum* genomes provide insights into the origin and evolution of allotetraploid cotton. Nat Genet 51: 739–748. 30886425 10.1038/s41588-019-0371-5

[B15] Hu, Y., Z. Han, W. Shen, Y. Jia, L. He, Z. Si, Q. Wang, L. Fang, X. Du and T. Zhang (2022) Identification of candidate genes in cotton associated with specific seed traits and their initial functional characterization in *Arabidopsis*. Plant J 112: 800–811. 36121755 10.1111/tpj.15982

[B16] Huang, T. and F.B. Hu (2015) Gene-environment interactions and obesity: recent developments and future directions. BMC Med Genomics 8: S2. 10.1186/1755-8794-8-S1-S2 PMC4315311 25951849

[B17] Ji, D., G. Zeng and J. Zhu (1985) Analysis of oil and amino acid contents in four cultivated species of *Gossypium*. Acta Agriculturae Universitatis Zhejiangensis 11: 257–262 (in Chinese with English summary).

[B18] Jia, F., F.D. Sun, J.W. Li, A.Y. Liu, Y.Z. Shi, W.K. Gong, T. Wang, Z. Liu and Y.L. Yuan (2011) Identification of QTL for boll weight and lint percentage of Upland cotton (*Gossypium hirsutum* L.) RIL population in multiple environments. Mol Plant Breed 9: 318–326 (in Chinese with English summary).

[B19] Korte, A., B. Vilhjálmsson, V. Segura, A. Platt, Q. Long and M. Nordborg (2012) A mixed-model approach for genome-wide association studies of correlated traits in structured populations. Nat Genet 44: 1066–1071. 22902788 10.1038/ng.2376 PMC3432668

[B20] Li, C., W. Guo, X. Ma and T. Zhang (2008) Tagging and mapping of QTL for yield and its components in upland cotton (*Gossypium hirsutum* L.) population with varied lint percentage. Cotton Sci 20: 163–169 (in Chinese with English summary).

[B21] Li, C., X. Wang, N. Dong, H. Zhao, Z. Xia, R. Wang, R. Converse and Q. Wang (2013a) QTL analysis for early-maturing traits in cotton using two upland cotton (*Gossypium hirsutum* L.) crosses. Breed Sci 63: 154–163. 23853509 10.1270/jsbbs.63.154 PMC3688376

[B22] Li, C., Y. Fu, R. Sun, Y. Wang and Q. Wang (2018a) Single-locus and multi-locus genome-wide association studies in the genetic dissection of fiber quality traits in upland cotton (*Gossypium hirsutum* L.). Front Plant Sci 9: 1083. 30177935 10.3389/fpls.2018.01083 PMC6109694

[B23] Li, C., Y. Wang, N. Ai, Y. Li and J. Song (2018b) A genome-wide association study of early-maturation traits in upland cotton based on the CottonSNP80K array. J Integr Plant Biol 60: 970–985. 29877621 10.1111/jipb.12673

[B24] Li, C., Y. Pu, X. Gao, Y. Cao, Y. Bao, Q. Xu, L. Du, J. Tan, Y. Zhu, H. Zhang et al. (2024) Detection of quantitative trait nucleotides (QTNs) and QTN-by-environment and QTN-by-QTN interactions for cotton early-maturity traits using the 3VmrMLM method. Ind Crop Prod 216: 118706.

[B25] Li, C.Q., G.S. Liu, H.H. Zhao, L.J. Wang, X.F. Zhang, Y. Liu, W.Y. Zhou, L.L. Yang, P.B. Li and Q.L. Wang (2013b) Marker-assisted selection of *Verticillium* wilt resistance in progeny populations of upland cotton derived from mass selection-mass crossing. Euphytica 191: 469–480.

[B26] Li, C.Q., N.J. Ai, Y.J. Zhu, Y.Q. Wang, X.D. Chen, F. Li, Q.Y. Hu and Q.L. Wang (2016) Association mapping and favourable allele exploration for plant architecture traits in upland cotton (*Gossypium hirsutum* L.) accessions. J Agric Sci 154: 567–583.

[B27] Li, H., L. Zhang, S. Gao and J. Wang (2025) Prediction by simulation in plant breeding. Crop J 13: 501–509.

[B28] Li, M., Y. Zhang, Z. Zhang, Y. Xiang, M. Liu, Y. Zhou, J. Zuo, H. Zhang, Y. Chen and Y. Zhang (2022) A compressed variance component mixed model for detecting QTNs and QTN-by-environment and QTN-by-QTN interactions in genome-wide association studies. Mol Plant 15: 630–650. 35202864 10.1016/j.molp.2022.02.012

[B29] Li, Y., J. Wang, L. Qiu, Y. Ma, X. Li and J. Wan (2010) Crop molecular breeding in China: current status and perspectives. Acta Agron Sin 36: 1425–1430 (in Chinese with English summary).

[B30] Lipka, A.E., F. Tian, Q. Wang, J. Peiffer, M. Li, P.J. Bradbury, M.A. Gore, E.S. Buckler and Z. Zhang (2012) GAPIT: genome association and prediction integrated tool. Bioinformatics 28: 2397–2399. 22796960 10.1093/bioinformatics/bts444

[B31] Lippert, C., J. Listgarten, Y. Liu, C.M. Kadie, I.D. Robert and D. Heckerman (2011) FaST linear mixed models for genome-wide association studies. Nat Methods 8: 833–835. 21892150 10.1038/nmeth.1681

[B32] Liu, D., F. Liu, X. Shan, J. Zhang, S. Tang, X. Fang, X. Liu, W. Wang, X. Tan, Z. Teng et al. (2015) Construction of a high-density genetic map and lint percentage and cottonseed nutrient trait QTL identification in upland cotton (*Gossypium hirsutum* L.). Mol Genet Genomics 22: 1683–1700. 10.1007/s00438-015-1027-5 25796191

[B33] Liu, X., J. Li, X. Yu, Y. Shi, J. Fei, F. Sun, A. Liu, J. Gong, H. Shang and W. Gong (2013) Identification of QTL for cottonseed oil and protein content in upland cotton (*Gossypium hirsutum* L.) based on a RIL population. Mol Plant Breed 11: 520–528 (in Chinese with English summary).

[B34] Lyu, F., F. Han, C. Ge, W. Mao, L. Chen, H. Hu, G. Chen, Q. Lang and C. Fang (2023) OmicStudio: A composable bioinformatics cloud platform with real-time feedback that can generate high-quality graphs for publication. iMeta e85. 38868333 10.1002/imt2.85 PMC10989813

[B35] Mei, H., N. Ai, X. Zhang, Z. Ning and T. Zhang (2014) QTLs conferring FOV 7 resistance detected by linkage and association mapping in upland cotton. Euphytica 197: 237–249.

[B36] Meuwissen, T.H.E., B.J. Hayes and M.E. Goddard (2001) Prediction of total genetic value using genome-wide dense marker maps. Genetics 157: 1819–1829. 11290733 10.1093/genetics/157.4.1819 PMC1461589

[B37] Moore, R., F. Casale, J. Jan, D. Horta, B. Consortium and L. Franke (2019) A linear mixed-model approach to study multivariate gene-environment interactions. Nat Genet 51: 180–186. 30478441 10.1038/s41588-018-0271-0 PMC6354905

[B38] Ning, C., W. Wang, D. Kang, R. Mrode, L. Zhou, S. Xu and J. Liu (2018) A rapid epistatic mixed-model association analysis by linear retransformations of genomic estimated values. Bioinformatics 34: 1817–1825. 29342229 10.1093/bioinformatics/bty017 PMC5972602

[B39] Peleman, J. and J. Voort (2003) Breeding by design. Trends Plant Sci 8: 330–334. 12878017 10.1016/S1360-1385(03)00134-1

[B40] Purcell, S., B. Neale, K. Todd-Brown, L. Thomas, M.A.R. Ferreira, D. Bender, J. Maller, P. Sklar, P.I.W. de Bakker, M.J. Daly et al. (2007) PLINK: a tool set for whole-genome association and population-based linkage analyses. Am J Hum Genet 81: 559–575. 17701901 10.1086/519795 PMC1950838

[B41] Qiu, S., Q. Lu, H. Yu and X. Ni (2018) The development and application of rice whole genome selection breeding platform. Chinese Bulletin of Life Sciences 30: 1120–1128.

[B42] Robinson, M.R., G. English, G. Moser, L.R. Lioyd-Jones, M.A. Triplett, Z. Zhu, I.M. Nolte, J. van Vliet-Ostaptchouk, H. Snieder, E. Esko et al. (2017) Genotype-covariate interaction effects and the heritability of adult body mass index. Nat Genet 49: 1174–1181. 28692066 10.1038/ng.3912

[B43] Saeed, M., W. Guo and T. Zhang (2014) Association mapping for salinity tolerance in cotton (*Gossypium hirsutum* L.) germplasm from US and diverse regions of China. Aust J Crop Sci 8: 338–346.

[B44] Segura, V., B. Vilhjálmsson, A. Platt, A. Korte, Ü. Seren, Q. Long and M. Nordborg (2012) An efficient multi-locus mixed-model approach for genome-wide association studies in structured populations. Nat Genet 44: 825–830. 22706313 10.1038/ng.2314 PMC3386481

[B45] Shen, X., W. Guo, X. Zhu, Y. Yuan, J. Yu, R. Kohel and T. Zhang (2005) Molecular mapping of QTLs for fiber qualities in three diverse lines in upland cotton using SSR markers. Mol Breed 15: 169–181.

[B46] Song, X. and T. Zhang (2007) Identification of quantitative traits loci controlling seed physical and nutrient traits in cotton. Seed Sci Res 17: 243–251.

[B47] Song, X. and T. Zhang (2009) Quantitative trait loci controlling plant architectural traits in cotton. Plant Sci 177: 317–323.

[B48] Su, C., W. Lu, T. Zhao and J. Gai (2010) Verification and fine-mapping of QTL conferring days to flowering in soybean using residual heterozygous lines. Chin Sci Bull 55: 499–508.

[B49] Sul, J.H. and E. Eskin (2013) Mixed models can correct for population structure for genomic regions under selection. Nat Rev Genet 14: 300. 10.1038/nrg2813-c1 23438871

[B50] Sun, Z., X. Wang, Z. Liu, Q. Gu, Y. Zhang, Z. Li, H. Ke, J. Yang, J. Wu, L. Wu et al. (2017) Genome-wide association study discovered genetic variation and candidate genes of fibre quality traits in *Gossypium hirsutum* L. Plant Biotechnol J 15: 982–996. 28064470 10.1111/pbi.12693 PMC5506648

[B51] Wang, D., H. Tang, J. Liu, S. Xu, Q. Zhang and C. Ning (2020) Rapid epistatic mixed-model association studies by controlling multiple polygenic effects. Bioinformatics 36: 4833–4837. 32614415 10.1093/bioinformatics/btaa610

[B52] Wang, J., X. Wan, H. Li, W.H. Pfeiffer, J. Crouch and J. Wan (2007) Application of identified QTL-marker associations in rice quality improvement through a design breeding approach. Theor Appl Genet 115: 87–100. 17479243 10.1007/s00122-007-0545-x

[B53] Wang, J., H. Li, L. Zhang and L. Meng (2012) Users’ manual of QTL IciMapping version 3.2, 50–81. The Quantitative Genetics Group, Institute of Crop Science, Chinese Academy of Agricultural Sciences (CAAS), Beijing, China.

[B54] Wang, S., J. Feng, W. Ren, B. Huang, L. Zhou, Y. Wen, J. Zhang, J. Dunwell, S. Xu and Y. Zhang (2016) Improving power and accuracy of genome-wide association studies via a multi-locus mixed linear model methodology. Sci Rep 6: 19444. 26787347 10.1038/srep19444 PMC4726296

[B55] Wang, Y., G. Li, X. Guo, R. Sun, T. Dong, Q. Yang, Q. Wang and C. Li (2019) Dissecting the genetic architecture of seed-cotton and lint yields in upland cotton using genome-wide association mapping. Breed Sci 69: 611–620. 31988625 10.1270/jsbbs.19057 PMC6977443

[B56] Wei, N., S. Zhang, Y. Liu, J. Wang, B. Wu, J. Zhao, L. Qiao, X. Zheng, J. Wang and J. Zheng (2022) Genome-wide association study of coleoptile length with Shanxi wheat. Front Plant Sci 13: 1016551. 36212294 10.3389/fpls.2022.1016551 PMC9532578

[B57] Wen, Y., H. Zhang, Y. Ni, B. Huang, J. Zhang, J. Feng, S. Wang, J. Dunwell, Y. Zhang and R. Wu (2018) Methodological implementation of mixed linear models in multi-locus genome-wide association studies. Brief Bioinform 19: 700–712. 28158525 10.1093/bib/bbw145 PMC6054291

[B58] Wen, Y.J., X. Wu, S. Wang, L. Han, B. Shen, Y. Wang and J. Zhang (2023) Identification of QTN-by-environment interactions for yield related traits in maize under multiple abiotic stresses. Front Plant Sci 14: 1050313. 36875585 10.3389/fpls.2023.1050313 PMC9975332

[B59] Xu, J., Y. Xing, Y. Xu and J. Wan (2021) Breeding by design for future rice: genes and genome technologies. Crop J 9: 491–496.

[B60] Xu, S. (2010) An expectation-maximization algorithm for the Lasso estimation of quantitative trait locus effects. Heredity 105: 483–494. 20051978 10.1038/hdy.2009.180

[B61] Yu, J., S. Yu, S. Fan, M. Song, H. Zhai, X. Li and J. Zhang (2012) Mapping quantitative trait loci for cottonseed oil, protein and gossypol content in a *Gossypium hirsutum* × *Gossypium barbadense* backcross inbred line population. Euphytica 187: 191–201. 10.1007/s00122-012-1980-x 23064252

[B62] Yuan, Y., X. Wang, L. Wang, H. Xing, Q. Wang, M. Saeed, J. Tao, W. Feng, G. Zhang, X. Song et al. (2018) Genome-wide association study identifies candidate genes related to seed oil composition and protein content in *Gossypium hirsutum* L. Front Plant Sci 9: 1359. 30405645 10.3389/fpls.2018.01359 PMC6204537

[B63] Zhang, F., Z. Zhu, X. Tong, Z. Zhu, T. Qi and J. Zhu (2015a) Mixed linear model approaches of association mapping for complex traits based on omics variants. Sci Rep 5: 10298. 26223539 10.1038/srep10298 PMC5155518

[B64] Zhang, L., H. Li and J. Wang (2022) Blib is a multi-module simulation platform for genetics studies and intelligent breeding. Commun Biol 5: 1167. 36323773 10.1038/s42003-022-04151-9 PMC9630530

[B65] Zhang, T., Y. Hu, W. Jiang, L. Fang, X. Guan, J. Chen, C.A. Saski, M. Scheffler, A.M. Stelly, A.M. Hulse-Kemp et al. (2015b) Sequencing of allotetraploid cotton (*Gossypium hirsutum* L. acc. TM-1) provides a resource for fiber improvement. Nat Biotechnol 33: 531–537. 25893781 10.1038/nbt.3207

[B66] Zhao, G. (2002) The research on nutritious property, process of cottonseed protein and its application. Heilongjiang Agricultural Sciences 4: 41–43 (in Chinese with English summary).

[B67] Zhao, Q., X.S. Shi, T. Wang, Y. Chen, R. Yang, J. Mi, Y.W. Zhang and Y.M. Zhang (2023) Identification of QTNs, QTN-by-environment interactions, and their candidate genes for grain size traits in main crop and ratoon rice. Front Plant Sci 14: 1119218. 36818826 10.3389/fpls.2023.1119218 PMC9933869

[B68] Zhao, Y., H. Wang, W. Chen and Y. Li (2014) Genetic structure, linkage disequilibrium and association mapping of verticillium wilt resistance in elite cotton (*Gossypium hirsutum* L.) germplasm population. PLoS One 9: e86308. 24466016 10.1371/journal.pone.0086308 PMC3900507

[B69] Zhou, X. and M. Stephens (2012) Genome-wide efficient mixed-model analysis for association studies. Nat Genet 44: 821–824. 22706312 10.1038/ng.2310 PMC3386377

[B70] Zhou, X., P. Carbonetto and M. Stephens (2013) Polygenic modeling with Bayesian sparse linear mixed models. PLoS Genet 9: e1003264. 23408905 10.1371/journal.pgen.1003264 PMC3567190

