# Supplementary material for: A genome-wide association study to elucidate the genetic basis of the cottonseed protein content in Upland cotton (Gossypium hirsutum L.)
Source: Breed Sci. 2026 Jan 15;76(2):121–32. doi: 10.1270/jsbbs.24092 (PMC13494328; doi:10.1270/jsbbs.24092)
Supplement: Supplementary file 1 [file 76_121_s1.pdf]

doi: 10.1270/jsbbs.24092

Supplemental Data 1. The PLINK command scripts for filtering all polymorphic markers

```
plink --vcf vcf  --maf 0.05  --geno 0.2  --recode vcf-iid  --recode vcf-iid
--allow-extra-chr  --chr-set 95  --out filter.snp
vcftools --vcf vcf  --out filter.snp --plink  --out SNP
plink --file SNP  --allow-extra-chr  --chr-set 95 --indep-pairwise 500kb 5 0.7  --out
ld
```
