# Supplementary material for: A genome-wide association study to elucidate the genetic basis of the cottonseed protein content in Upland cotton (Gossypium hirsutum L.)
Source: Breed Sci. 2026 Jan 15;76(2):121–32. doi: 10.1270/jsbbs.24092 (PMC13494328; doi:10.1270/jsbbs.24092)
Supplement: Supplementary file 2 [file 76_121_s2.pdf]

doi: 10.1270/jsbbs.24092

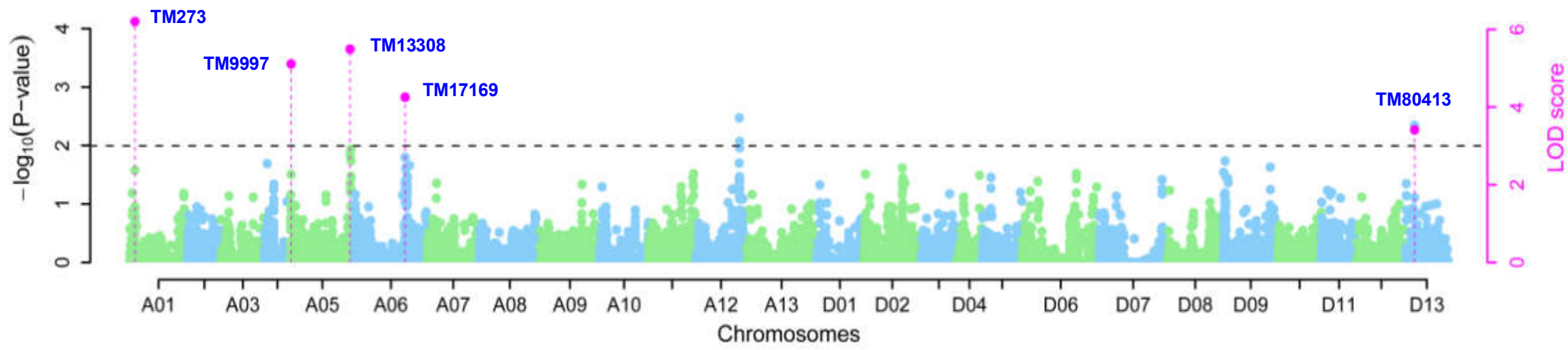

Supplemental Fig. 1. Manhattan plot for the QEIs related to cottonseed protein content detected in multi-environment by 3VmrMLM-Multi_env. The left y-axis shows the -$\log_{10}P$ values obtained from the genome-wide scan of all markers in the first step of 3VmrMLM, while the right y-axis shows the LOD values of the QEIs obtained through the likelihood ratio test in the second step of 3VmrMLM, with the threshold line set at LOD = 3.0

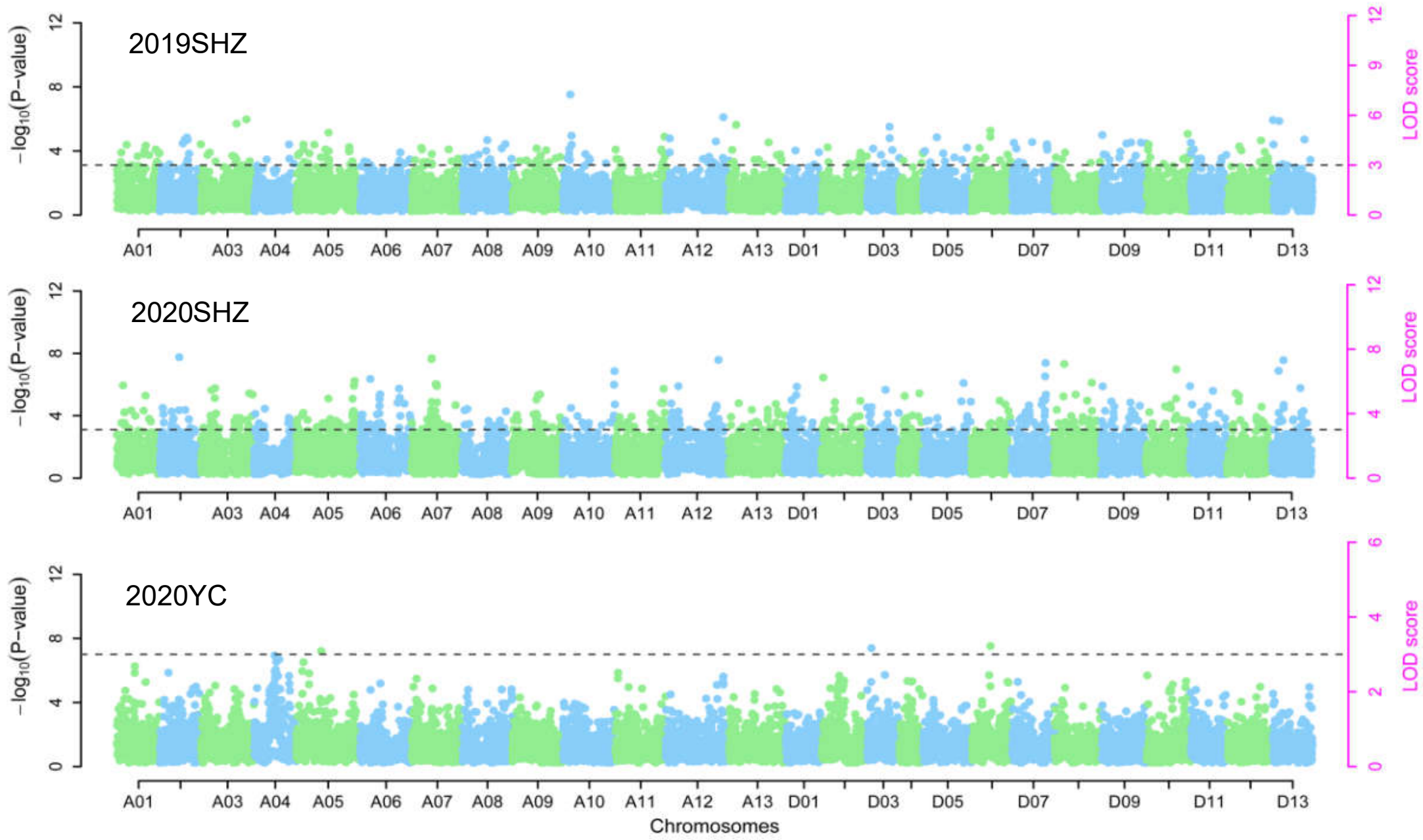

Supplemental Fig. 2. Manhattan plot for the QQIs related to cottonseed protein content detected in three environments by 3VmrMLM-Epistasis. The left y-axis shows the -$\log_{10}P$ values obtained from the genome-wide scan of all marker pairs in the first step of 3VmrMLM, while the right y-axis shows the LOD values of the QQIs obtained through the likelihood ratio test in the second step of 3VmrMLM, with the threshold line set at LOD = 3.0
