# Supplementary material for: A genome-wide association study to elucidate the genetic basis of the cottonseed protein content in Upland cotton (Gossypium hirsutum L.)
Source: Breed Sci. 2026 Jan 15;76(2):121–32. doi: 10.1270/jsbbs.24092 (PMC13494328; doi:10.1270/jsbbs.24092)
Supplement: Supplementary file 3 [file 76_121_s3.pdf]

doi: 10.1270/jsbbs.24092

Supplemental Table 1. Names and origins of 152 Upland cotton accessions

No	Accession	Ecoregional origin	Region
1	Xinluzao1	Northwestern Ghina	Xinjiang, China
2	Xinluzao2	Northwestern Ghina	Xinjiang, China
3	Xinluzao3	Northwestern Ghina	Xinjiang, China
4	Xinluzao6	Northwestern Ghina	Xinjiang, China
5	Xinluzao7	Northwestern Ghina	Xinjiang, China
6	Xinluzao8	Northwestern Ghina	Xinjiang, China
7	Xinluzao9	Northwestern Ghina	Xinjiang, China
8	Xinluzao10	Northwestern Ghina	Xinjiang, China
9	Xinluzao11	Northwestern Ghina	Xinjiang, China
10	Xinluzao12	Northwestern Ghina	Xinjiang, China
11	Xinluzao13	Northwestern Ghina	Xinjiang, China
12	Xinluzao18	Northwestern Ghina	Xinjiang, China
13	Xinluzao19	Northwestern Ghina	Xinjiang, China
14	Xinluzao20	Northwestern Ghina	Xinjiang, China
15	Xinluzao22	Northwestern Ghina	Xinjiang, China
16	Xinluzao23	Northwestern Ghina	Xinjiang, China
17	Xinluzao25	Northwestern Ghina	Xinjiang, China
18	Xinluzao26	Northwestern Ghina	Xinjiang, China
19	Xinluzao27	Northwestern Ghina	Xinjiang, China
20	Xinluzao28	Northwestern Ghina	Xinjiang, China
21	Xinluzao29	Northwestern Ghina	Xinjiang, China
22	Xinluzao30	Northwestern Ghina	Xinjiang, China
23	Xinluzao31	Northwestern Ghina	Xinjiang, China
24	Xinluzao32	Northwestern Ghina	Xinjiang, China
25	Xinluzao35	Northwestern Ghina	Xinjiang, China
26	Xinluzao36	Northwestern Ghina	Xinjiang, China
27	Xinluzao37	Northwestern Ghina	Xinjiang, China
28	Xinluzao38	Northwestern Ghina	Xinjiang, China
29	Xinluzao39	Northwestern Ghina	Xinjiang, China
30	Xinluzao40	Northwestern Ghina	Xinjiang, China
31	Xinluzao41	Northwestern Ghina	Xinjiang, China
32	Xinluzao42	Northwestern Ghina	Xinjiang, China
33	Xinluzao46	Northwestern Ghina	Xinjiang, China
34	Xinluzao47	Northwestern Ghina	Xinjiang, China
35	Xinluzao48	Northwestern Ghina	Xinjiang, China
36	Xinluzhong36	Northwestern Ghina	Xinjiang, China
37	CRI8	Yellow River	Henan, China
38	CRI10	Yellow River	Henan, China
39	CRI12	Yellow River	Henan, China
40	CRI13	Yellow River	Henan, China

41	CRI14	Yellow River	Henan, China
42	CRI15	Yellow River	Henan, China
43	Zhong1707	Yellow River	Henan, China
44	CRI17	Yellow River	Henan, China
45	CRI18	Yellow River	Henan, China
46	CRI19	Yellow River	Henan, China
47	CRI20	Yellow River	Henan, China
48	CRI22	Yellow River	Henan, China
49	CRI23	Yellow River	Henan, China
50	CRI24	Yellow River	Henan, China
51	CRI25	Yellow River	Henan, China
52	CRI26	Yellow River	Henan, China
53	CRI27	Yellow River	Henan, China
54	CRI30	Yellow River	Henan, China
55	CRI31	Yellow River	Henan, China
56	CRI33	Yellow River	Henan, China
57	CRI34	Yellow River	Henan, China
58	CRI35	Yellow River	Henan, China
59	CRI36	Yellow River	Henan, China
60	CRI37	Yellow River	Henan, China
61	CRI40	Yellow River	Henan, China
62	CRI50	Yellow River	Henan, China
63	CRI58	Yellow River	Henan, China
64	CRI64	Yellow River	Henan, China
65	Zhongzhimian2	Yellow River	Henan, China
66	Liaomian4	Northern China	Liaoning, China
67	Liaomian5	Northern China	Liaoning, China
68	Liaomian7	Northern China	Liaoning, China
69	Liaomian8	Northern China	Liaoning, China
70	Liaomian10	Northern China	Liaoning, China
71	Liaomian16	Northern China	Liaoning, China
72	Liaomian18	Northern China	Liaoning, China
73	Liaomian19	Northern China	Liaoning, China
74	Liaomian23	Northern China	Liaoning, China
75	Yumian1	Yellow River	Henan, China
76	Yumian5	Yellow River	Henan, China
77	Yumian7	Yellow River	Henan, China
78	Yumian9	Yellow River	Henan, China
79	Yumian12	Yellow River	Henan, China
80	Yumian21	Yellow River	Henan, China
81	Lumian1	Yellow River	Shandong, China
82	Lumian4	Yellow River	Shandong, China

83	Lumian6	Yellow River	Shandong, China
84	Lumian10	Yellow River	Shandong, China
85	Lumianyan21	Yellow River	Shandong, China
86	Lumianyan28	Yellow River	Shandong, China
87	Lumianyan29	Yellow River	Shandong, China
88	Shiyuan321	Yellow River	Hebei, China
89	Fenwu195	Yellow River	Unknown
90	Qianjing9	Yangtze River	Unknown
91	Xiangmian3	Yangtze River	Hunan, China
92	Xiangmian10	Yangtze River	Hunan, China
93	Yanmian48	Yangtze River	Jiangsu, China
94	Jiangsumian1	Yangtze River	Jiangsu, China
95	Sumian1	Yangtze River	Jiangsu, China
96	Sumian6	Yangtze River	Jiangsu, China
97	Sumian9	Yangtze River	Jiangsu, China
98	Sumian10	Yangtze River	Jiangsu, China
99	Sumian12	Yangtze River	Jiangsu, China
100	Sumian16	Yangtze River	Jiangsu, China
101	Xuzhou142	Yangtze River	Jiangsu, China
102	Qiannong465	Yangtze River	Guizhou, China
103	Dongting1	Yangtze River	Hunan, China
104	Xinqiu1	Yellow River	Shandong, China
105	Simian2	Yangtze River	Jiangsu, China
106	Simian3	Yangtze River	Jiangsu, China
107	Simian4	Yangtze River	Jiangsu, China
108	Chuanmian56	Yangtze River	Sichuan, China
109	Jinzhong169	Northern China	Shanxi, China
110	Jinzhong200	Northern China	Shanxi, China
111	Jinmian5	Northern China	Shanxi, China
112	Jinmian6	Northern China	Shanxi, China
113	Jinmian8	Northern China	Shanxi, China
114	Jinmian9	Northern China	Shanxi, China
115	Jinmian13	Yellow River	Shanxi, China
116	Jinmian14	Northern China	Shanxi, China
117	Jinmian24	Northern China	Shanxi, China
118	Jinmian29	Yellow River	Shanxi, China
119	Jinmian36	Yellow River	Shanxi, China
120	Jinmian45	Yellow River	Shanxi, China
121	Jinzhoumian1	Northern China	Liaoning, China
122	Jinzhoumian2	Northern China	Liaoning, China
123	Jinzhoumian4	Northern China	Liaoning, China
124	Jinzhoumian5	Northern China	Liaoning, China

125	Guoxinmian3	Yellow River	Hebei, China
126	Jimian958	Yellow River	Hebei, China
127	Jimian1	Yellow River	Hebei, China
128	Jimian7	Yellow River	Hebei, China
129	Jimian12	Yellow River	Hebei, China
130	Ejing1	Yangtze River	Hubei, China
131	Emian3	Yangtze River	Hubei, China
132	Emian14	Yangtze River	Hubei, China
133	Esha28	Yangtze River	Hubei, China
134	Jing8891	Yangtze River	Hubei, China
135	Daihongdai	Yangtze River	Hubei, China
136	STV2B	USA	Unknown
137	DPL15	USA	Unknown
138	DPL16	USA	Unknown
139	Shanmian4	Yellow River	Shaanxi, China
140	Shan1155	Yellow River	Shaanxi, China
141	Shan2365	Yellow River	Shaanxi, China
142	Handan802	Yellow River	Hubei, China
143	Handan885	Yellow River	Hubei, China
144	Ganmian8	Yangtze River	Jiangxi, China
145	Uganda3	Uganda	Unknown
146	Lvzao254	Northwestern China	Unknown
147	Shixuan87	Uzbekistan	Unknown
148	Beishinuo	USA	Unknown
149	99M4	USA	Unknown
150	99M7	USA	Unknown
151	99M8	USA	Unknown
152	Lamagan77	Northwestern China	Xinjiang, China

Supplemental Table 2. Correlation analysis for the cottonseed protein content in three environments

Environment	2019SHZ	2020SHZ	2020YC
2019SHZ	1		
2020SHZ	0.5904***	1	
2020YC	0.2867**	0.4639***	1

** and *** represent significance at the $P < 0.01$ and $P < 0.001$ level, respectively.

Supplemental Table 3. Summary of SNPs on 26 *Gossypium hirsutum* chromosomes

Chr.	Size (kb)	No. of SNP	SNP density(kb/SNP)
A01	99721.563	2166	46.04
A02	83028.799	1273	65.22
A03	99890.971	1624	61.51
A04	62700.837	964	65.04
A05	91591.972	2422	37.82
A06	102777	2724	37.73
A07	77971.021	1966	39.66
A08	103322.572	2289	45.14
A09	74503.899	2223	33.52
A10	100783.4	1851	54.45
A11	93136.106	1762	52.86
A12	87020.072	1912	45.51
A13	79865.158	2636	30.30
D01	61280.956	1748	35.06
D02	67183.532	2188	30.71
D03	46583.445	1321	35.26
D04	50970.11	972	52.44
D05	61549.545	1491	41.28
D06	64275.813	2906	22.12
D07	53938.274	2538	21.25
D08	65281.74	2127	30.69
D09	50737.711	2040	24.87
D10	63357.196	1635	38.75
D11	65966.023	1330	49.60
D12	58957.791	1843	31.99
D13	60461.627	1582	38.22

Supplemental Table 4. QTNs for cottonseed protein content detected by six GWAS methods

Method	Env.	Marker	Chr.	Posi. (bp)	LOD	A	D	PVE (%)	–log10P	Sig.
GEMMA-LM	2019SHZ	TM80005	D13	166212				8.24	5.06	SIG
FaST-LMM	2019SHZ	TM80005	D13	166212				8.24	5.07	SIG
	2020YC	TM59869	D06	20608253				9.76	4.94	SIG
	2020YC	TM28555	A08	72270912				6.18	4.84	SIG
	2020YC	TM14460	A06	32066392				5.71	4.70	SIG
EMMAX	2019SHZ	TM57181	D05	10112866				6.67	5.64	SIG
	2019SHZ	TM29000	A08	80447154				9.16	5.40	SIG
	2019SHZ	TM42986	A12	83905690				10.38	5.19	SIG
	2019SHZ	TM66567	D07	49931922				6.62	4.95	SIG
3VmrMLM-Single_env	2019SHZ	TM32199	A09	46704933	6.68	-0.25	-1.53	3.7921	6.67	SIG
	2019SHZ	TM37232	A11	6733232	33.36	2.00	1.19	5.1066	33.36	SIG
	2019SHZ	TM38077	A11	25344206	6.22	-0.13	-4.48	3.752	6.22	SIG
	2019SHZ	TM39928	A11	92537253	5.35	-0.62		2.2682	6.16	SIG
	2019SHZ	TM40095	A12	2063413	10.05	0.98	-0.16	7.1276	10.05	SIG
	2019SHZ	TM42987	A12	83911906	8.95	0.81		5.5876	9.86	SIG
	2019SHZ	TM49452	D01	47561789	4.59	-0.12	-1.11	1.7997	4.59	SUG
	2019SHZ	TM49462	D01	48532371	4.59	-0.12	-1.11	1.7997	4.59	SUG
	2019SHZ	TM54968	D03	37772356	11.37	-0.96		7.9352	12.34	SIG
	2019SHZ	TM66567	D07	49931922	6.20	0.76	0.07	2.8585	6.20	SIG
	2019SHZ	TM71935	D09	32343135	6.95	-0.83	0.05	4.4414	6.95	SIG
	2019SHZ	TM76938	D11	56159244	13.34	0.30	2.37	6.2358	13.34	SIG
	2019SHZ	TM80339	D13	5309480	22.85	-1.52	1.04	9.0235	22.85	SIG
	2020SHZ	TM6227	A03	5860961	7.18	1.31	0.71	11.8212	7.18	SIG
	2020SHZ	TM14428	A06	31716814	4.67	-1.04	-0.50	5.1037	4.67	SUG

	2020SHZ	TM29819	A08	100218275	14.19	-2.00	-0.78	11.0895	14.19	SIG
	2020SHZ	TM42843	A12	79165430	11.29	-1.57		11.0435	12.25	SIG
	2020SHZ	TM46947	A13	66302165	5.20	-0.05	-2.43	6.6538	5.19	SUG
	2020SHZ	TM76375	D11	48924784	8.07	1.30		10.9604	8.97	SIG
	2020SHZ	TM80417	D13	7825364	3.85	-0.21	-2.14	5.1008	3.85	SUG
	2020YC	TM4804	A02	44797495	6.70	-0.68	-0.72	5.0569	6.70	SIG
	2020YC	TM6436	A03	11642099	8.79	0.77		6.5872	9.70	SIG
	2020YC	TM9790	A04	59805084	8.86	0.78		4.7534	9.77	SIG
	2020YC	TM19997	A07	26433432	3.83	-0.49		2.7932	4.58	SUG
	2020YC	TM37009	A11	1135555	10.40	0.88	0.75	6.4594	10.40	SIG
	2020YC	TM44163	A13	35146650	6.34	-0.66		5.0499	7.19	SIG
	2020YC	TM50134	D01	59324179	5.37	0.40	-1.49	4.7825	5.37	SUG
	2020YC	TM55883	D04	9376629	5.62	0.71	0.04	4.0257	5.62	SUG
	2020YC	TM59869	D06	20608253	8.60	0.77		6.8361	9.50	SIG
	2020YC	TM75084	D10	60224110	8.62	0.79		6.2943	9.53	SIG
	2020YC	TM75163	D10	61664718	6.20	-0.69		5.3105	7.04	SIG
3VmrMLM-Multi_env	Multi-env.	TM3544	A01	96886927	18.43	-1.01	0.89	5.5881	18.43	SIG
	Multi-env.	TM19958	A07	26115002	8.54	-0.66		3.652	9.45	SIG
	Multi-env.	TM32293	A09	48100370	4.42	-0.50	-0.34	2.0709	4.42	SUG
	Multi-env.	TM40095	A12	2063413	4.92	0.53	0.29	2.1487	4.92	SUG
	Multi-env.	TM46963	A13	66563407	4.41	0.08	-1.06	1.7781	4.41	SUG
	Multi-env.	TM54965	D03	37738332	12.26	0.83		4.0986	13.25	SIG
	Multi-env.	TM59327	D06	7539844	11.88	-0.23	-5.63	4.7222	11.88	SIG
	Multi-env.	TM62915	D06	59009603	13.93	-0.85		3.9328	14.94	SIG
	Multi-env.	TM63795	D07	12896010	13.22	0.95	-0.19	4.752	13.22	SIG
	Multi-env.	TM75163	D10	61664718	7.90	-0.68		3.7039	8.79	SIG

3VmrMLM-Epistasis	2019SHZ	TM3544	A01	96886927	5.11	-0.31	-0.81	1.6129	5.11	SUG
	2019SHZ	TM32248	A09	47348358	3.24	0.13	-0.79	2.5366	3.24	SUG
	2019SHZ	TM39928	A11	92537253	3.81	-0.22		0.2893	4.55	SUG
	2019SHZ	TM40095	A12	2063413	7.18	0.72	-0.22	4.2607	7.18	SIG
	2019SHZ	TM49452	D01	47561789	6.49	0.19	-0.75	4.7082	6.49	SIG
	2019SHZ	TM54968	D03	37772356	6.45	-1.22		12.8716	7.30	SIG
	2019SHZ	TM59327	D06	7539844	3.75	0.60	-3.48	11.2851	3.75	SUG
	2019SHZ	TM66568	D07	49935809	4.92	1.22	0.25	6.8552	4.92	SUG
	2019SHZ	TM71935	D09	32343135	6.50	-0.94	-0.04	5.5487	6.50	SIG
	2019SHZ	TM76938	D11	56159244	6.25	0.81	1.46	8.4437	6.25	SIG
	2019SHZ	TM80339	D13	5309480	8.37	-0.99	-0.29	2.2015	8.37	SIG
	2020SHZ	TM273	A01	5351569	9.35	0.87		5.5626	10.28	SIG
	2020SHZ	TM6227	A03	5860961	3.28	0.39	0.05	1.0166	3.28	SUG
	2020SHZ	TM14428	A06	31716814	8.30	-0.76	0.07	3.0527	8.30	SIG
	2020SHZ	TM17464	A06	90766701	10.37	0.83		4.5622	11.32	SIG
	2020SHZ	TM29819	A08	100218275	10.56	-1.29	-0.08	5.1548	10.56	SIG
	2020SHZ	TM37308	A11	8971069	7.28	-0.24	0.50	1.3809	7.28	SIG
	2020SHZ	TM37376	A11	13845872	8.96	0.07	-0.62	2.2616	8.96	SIG
	2020SHZ	TM40600	A12	10666734	6.08	-1.02		4.2514	6.92	SIG
	2020SHZ	TM48226	D01	7557305	21.91	0.00	-1.91	6.7245	21.91	SIG
	2020SHZ	TM50955	D02	13583390	8.32	-0.09	0.62	2.7484	8.32	SIG
	2020SHZ	TM58784	D05	59749743	16.53	1.06	-0.50	5.281	16.53	SIG
	2020SHZ	TM63430	D07	6019337	4.29	0.42	0.00	0.9993	4.29	SUG
	2020SHZ	TM63753	D07	11110213	9.17	-0.56	0.39	2.3301	9.17	SIG
	2020SHZ	TM72484	D09	42070439	5.29	0.73	-0.05	3.0894	5.29	SUG
	2020SHZ	TM80417	D13	7825364	8.01	0.33	-0.91	4.5906	8.01	SIG

2020YC	TM1119	A01	21344836	4.10	-1.69		14.5857	4.86	SUG
2020YC	TM11103	A05	23962836	3.56	1.04		7.6504	4.28	SUG
2020YC	TM37571	A11	17158562	3.18	0.30	0.66	3.7163	3.18	SUG
2020YC	TM59869	D06	20608253	4.53	0.86		8.3652	5.30	SUG

LOD, logarithm of the odds score; A, additive effect; D, dominance effect; PVE (%), phenotypic variation explained; Sig., significance level; SIG, significant; SUG, suggested

Supplemental Table 5. Functional annotation of the candidate genes for two stable QTNs, five QEIs, and four significant QQIs according to GO, KEGG, and TAIR databases

Loci	SNP	#GeneID	GO	KEGG	ArabidopsisID	ArabDesc
Two stable QTNs	TM40095	*GH_A12G0120*	GO:0004683(calmodulin-dependent protein kinase activity);GO:0005516(calmodulin binding);GO:0005634(nucleus);GO:0005737(cytoplasm);GO:0005886(plasma membrane);GO:0009738(abscisic acid-activated signaling pathway);GO:0009931(calcium-dependent protein serine/threonine kinase activity);GO:0018105(peptidyl-serine phosphorylation);GO:0035556(intracellular signal transduction);GO:0046777(protein autophosphorylation)	04626(Plant-pathogen interaction)	*AT2G17290*	Calcium-dependent protein kinase family protein
		GH_A12G0121	GO:0003735(structural constituent of ribosome);GO:0006412(translation)	NA	*AT2G17270*	phosphate transporter 3%3B3
		GH_A12G0125	GO:0004675(transmembrane receptor protein serine/threonine kinase activity);GO:0005524(ATP binding);GO:0005634(nucleus);GO:0005886(plasma membrane);GO:0006468(protein phosphorylation);GO:0006952(defense response);GO:0007166(cell surface receptor signaling pathway);GO:0009507(chloroplast);GO:0016301(kinase activity)	NA	*AT2G17220*	Protein kinase superfamily protein
		GH_A12G0126	GO:0005739(mitochondrion);GO:0043231(intracellular membrane-bounded organelle)	00500(Starch and sucrose metabolism)	*AT2G17210*	Tetratricopeptide repeat (TPR)-like superfamily protein
		GH_A12G0128	GO:0004609(phosphatidylserine decarboxylase activity);GO:0005509(calcium ion binding);GO:0005737(cytoplasm);GO:0005783(endoplasmic reticulum);GO:0005789(endoplasmic reticulum	00564(Glycerophospholipid metabolism)	*AT5G57190*	phosphatidylserine decarboxylase 2

	membrane);GO:0006646(phosphatidylethanolamine biosynthetic process);GO:0008654(phospholipid biosynthetic process);GO:0097708(intracellular vesicle)			
GH_A12G0129	GO:0003677(DNA binding);GO:0003700(DNA binding transcription factor activity);GO:0005634(nucleus);GO:0006351(transcription, DNA-templated);GO:0006355(regulation of transcription, DNA-templated);GO:0007275(multicellular organism development);GO:0010089(xylem development);GO:0043067(regulation of programmed cell death);GO:0048367(shoot system development)	NA	*AT5G64530*	xylem NAC domain 1
GH_A12G0134	GO:0002213(defense response to insect);GO:0004768(stearoyl-CoA 9-desaturase activity);GO:0006631(fatty acid metabolic process);GO:0006633(fatty acid biosynthetic process);GO:0006636(unsaturated fatty acid biosynthetic process);GO:0006952(defense response);GO:0008610(lipid biosynthetic process);GO:0009507(chloroplast);GO:0009534(chloroplast thylakoid);GO:0009536(plastid);GO:0009570(chloroplast stroma);GO:0009695(jasmonic acid biosynthetic process);GO:0042742(defense response to bacterium);GO:0045300(acyl-[acyl-carrier-protein] desaturase activity);GO:0046872(metal ion binding);GO:0051607(defense response to virus);GO:0055114(oxidation-reduction process);GO:0102786(stearoyl-[acp] desaturase	00061(Fatty acid biosynthesis);01040(Biosynthesis of unsaturated fatty acids)	*AT2G43710*	Plant stearoyl-acyl-carrier-protein desaturase family protein

	activity);GO:2000022(regulation of jasmonic acid mediated signaling pathway);GO:2000031(regulation of salicylic acid mediated signaling pathway)			
GH_A12G0135	GO:0003674(molecular_function);GO:0005886(plasma membrane);GO:0008150(biological_process);GO:0009507(chloroplast)	NA	*AT5G64090*	hyccin
GH_A12G0138	GO:0003674(molecular_function);GO:0005634(nucleus);GO:0008150(biological_process)	NA	*AT5G64180*	tropomyosin
GH_A12G0139	GO:0000325(plant-type vacuole);GO:0005774(vacuolar membrane);GO:0015079(potassium ion transmembrane transporter activity)	NA	*AT5G09400*	K+ uptake permease 7
GH_A12G0140	GO:0004040(amidase activity);GO:0005739(mitochondrion);GO:0006626(protein targeting to mitochondrion)	00330(Arginine and proline metabolism);00360(Phenylalanine metabolism);00380(Tryptophan metabolism)	*AT5G09420*	translocon at the outer membrane of chloroplasts 64-V
GH_A12G0143	GO:0005739(mitochondrion);GO:0005829(cytosol);GO:0009610(response to symbiotic fungus);GO:0016491(oxidoreductase activity);GO:0018580(nitronate monooxygenase activity);GO:0046686(response to cadmium ion);GO:0051213(dioxygenase activity);GO:0055114(oxidation-reduction process)	NA	*AT5G64250*	Aldolase-type TIM barrel family protein
GH_A12G0144	GO:0003723(RNA binding);GO:0005515(protein binding);GO:0005634(nucleus);GO:0005774(vacuolar membrane);GO:0008143(poly(A) binding);GO:0008150(biological_process);GO:0016607(nuclear speck);GO:0043621(protein self-association)	03015(mRNA surveillance pathway)	*AT5G65260*	RNA-binding (RRM/RBD/RNP motifs) family protein

GH_A12 G0146	GO:0000245(spliceosomal complex assembly);GO:0003729(mRNA binding);GO:0005686(U2 snRNP);GO:0005689(U12-type spliceosomal complex);GO:0071004(U2-type prespliceosome);GO:0071013(catalytic step 2 spliceosome)	03040(Spliceosome)	*AT5G64270*	splicing factor
GH_A12 G0148	GO:0003677(DNA binding);GO:0005524(ATP binding);GO:0005634(nucleus);GO:0006270(DNA replication initiation);GO:0008094(DNA-dependent ATPase activity)	NA	*AT2G14050*	minichromosome maintenance 9
GH_A12 G0149	GO:0043161(proteasome-mediated ubiquitin-dependent protein catabolic process);GO:0061630(ubiquitin protein ligase activity)	NA	*AT4G33565*	RING/U-box superfamily protein
GH_A12 G0150	GO:0004683(calmodulin-dependent protein kinase activity);GO:0005516(calmodulin binding);GO:0005634(nucleus);GO:0005737(cytoplasm);GO:0005886(plasma membrane);GO:0009738(abscisic acid-activated signaling pathway);GO:0009931(calcium-dependent protein serine/threonine kinase activity);GO:0018105(peptidyl-serine phosphorylation);GO:0035556(intracellular signal transduction);GO:0046777(protein autophosphorylation)	04626(Plant-pathogen interaction)	*AT1G12680*	phosphoenolpyruvate carboxylase-related kinase 2
GH_A12 G0151	NA	NA	-	-
GH_A12 G0152	NA	NA	-	-
GH_A12 G0153	NA	NA	-	-
GH_A12 G0154	GO:0003837(beta-ureidopropionase activity);GO:0005737(cytoplasm);GO:0005829(cytosol);GO:0006	00240(Pyrimidine metabolism);00410(beta-Alanine	*AT5G64370*	beta-ureidopropionase

	212(uracil catabolic process);GO:0006807(nitrogen compound metabolic process);GO:0016811(hydrolase activity, acting on carbon-nitrogen (but not peptide) bonds, in linear amides);GO:0019483(beta-alanine biosynthetic process);GO:0043562(cellular response to nitrogen levels)	metabolism);00770(Pantothenate and CoA biosynthesis)		
GH_A12 G0155	GO:0003824(catalytic activity);GO:0005634(nucleus);GO:0005737(cytoplasm);GO:0006769(nicotinamide metabolic process);GO:0008936(nicotinamidase activity);GO:0009737(response to abscisic acid);GO:0019365(pyridine nucleotide salvage)	NA	*AT2G22570*	nicotinamidase 1
GH_A12 G0159	GO:0000977(RNA polymerase II regulatory region sequence-specific DNA binding);GO:0001085(RNA polymerase II transcription factor binding);GO:0001228(transcriptional activator activity, RNA polymerase II transcription regulatory region sequence-specific DNA binding);GO:0003682(chromatin binding);GO:0005634(nucleus);GO:0005667(transcription factor complex);GO:0030154(cell differentiation)	NA	*AT3G21175*	ZIM-like 1
GH_A12 G0163	GO:0005524(ATP binding);GO:0005618(cell wall);GO:0005739(mitochondrion);GO:0005759(mitochondrial matrix);GO:0005774(vacuolar membrane);GO:0005794(Golgi apparatus);GO:0006457(protein folding);GO:0009408(response to heat);GO:0009507(chloroplast);GO:0009615(response to virus);GO:0009651(response to salt stress);GO:0034605(cellular response to heat);GO:0042026(protein refolding);GO:0046686(response to cadmium ion);GO:0051082(unfolded protein	03018(RNA degradation)	*AT5G09590*	mitochondrial HSO70 2

	binding);GO:0051085(chaperone cofactor-dependent protein refolding)			
GH_A12G0164	GO:0004040(amidase activity);GO:0005773(vacuole);GO:0005774(vacuolar membrane);GO:0005783(endoplasmic reticulum);GO:0005789(endoplasmic reticulum membrane);GO:0005794(Golgi apparatus);GO:0005886(plasma membrane);GO:0016021(integral component of membrane);GO:0042742(defense response to bacterium);GO:0047412(N-(long-chain-acyl)ethanolamine deacylase activity);GO:0070291(N-acylethanolamine metabolic process);GO:0102077(oleamide hydrolase activity);GO:0103073(anandamide amidohydrolase activity)	00970(Aminoacyl-tRNA biosynthesis)	*AT5G64440*	fatty acid amide hydrolase
GH_A12G0166	GO:0006351(transcription, DNA-templated);GO:0006355(regulation of transcription, DNA-templated);GO:0009631(cold acclimation);GO:0010150(leaf senescence);GO:0016592(mediator complex);GO:0042542(response to hydrogen peroxide);GO:0048364(root development)	00900(Terpenoid backbone biosynthesis)	*AT1G11760*	mediator of RNA polymerase II transcription subunit-like protein
GH_A12G0171	NA	NA	*AT3G49780*	phytosulfokine 4 precursor
GH_A12G0173	NA	NA	*AT5G65925*	hypothetical protein
GH_A12G0180	NA	NA	-	-
GH_A12	GO:0003674(molecular_function);GO:0005634(nucleus);GO:0008	NA	*AT3G19650*	cyclin-like protein

	G0181	150(biological_process)			
	GH_A12 G0182	GO:0003674(molecular_function);GO:0005739(mitochondrion);GO:0008150(biological_process)	00520(Amino sugar and nucleotide sugar metabolism)	*AT3G19615*	beta-1%2C4-xylosidase
	GH_A12 G0183	GO:0008270(zinc ion binding);GO:0009737(response to abscisic acid);GO:0016021(integral component of membrane)	NA	*AT2G14835*	RING/U-box superfamily protein
	GH_A12 G0186	GO:0005886(plasma membrane);GO:0006952(defense response);GO:0007165(signal transduction)	04016(MAPK signaling pathway - plant);04075(Plant hormone signal transduction);04626(Plant-pathogen interaction)	*AT3G43740*	Leucine-rich repeat (LRR) family protein
TM59869	*GH_D06 G1018*	GO:0003677(DNA binding);GO:0003700(DNA binding transcription factor activity);GO:0005634(nucleus);GO:0006351(transcription, DNA-templated);GO:0006355(regulation of transcription, DNA-templated);GO:0009873(ethylene-activated signaling pathway);GO:0043565(sequence-specific DNA binding);GO:0045893(positive regulation of transcription, DNA-templated)	NA	*AT5G11590*	Integrase-type DNA-binding superfamily protein
	GH_D06 G1020	GO:0003674(molecular_function);GO:0005886(plasma membrane);GO:0008150(biological_process);GO:0016021(integral component of membrane)	NA	*AT4G37235*	Uncharacterized protein family (UPF0497)
	GH_D06 G1021	GO:0003674(molecular_function);GO:0008150(biological_process)	NA	*AT5G11600*	hypothetical protein
	GH_D06 G1029	GO:0005737(cytoplasm);GO:0005773(vacuole);GO:0005774(vacuolar membrane);GO:0005794(Golgi apparatus);GO:0005886(plasma membrane);GO:0009414(response	NA	*AT3G63520*	carotenoid cleavage dioxygenase 1

	to water deprivation);GO:0009506(plasmodesma);GO:0016118(carotenoid catabolic process);GO:0016121(carotene catabolic process);GO:0016124(xanthophyll catabolic process);GO:0045549(9-cis-epoxycarotenoid dioxygenase activity);GO:0046872(metal ion binding);GO:0055114(oxidation-reduction process)			
GH_D06G1031	GO:0004462(lactoylglutathione lyase activity);GO:0005739(mitochondrion);GO:0005975(carbohydrate metabolic process);GO:0009409(response to cold);GO:0009507(chloroplast);GO:0009570(chloroplast stroma);GO:0009579(thylakoid);GO:0010319(stromule);GO:0019243(methylglyoxal catabolic process to D-lactate via S-lactoyl-glutathione);GO:0031977(thylakoid lumen);GO:0046872(metal ion binding)	00620(Pyruvate metabolism)	*AT1G67280*	Glyoxalase/Bleomycin resistance protein/Dioxygenase superfamily protein
GH_D06G1033	GO:0034975(protein folding in endoplasmic reticulum);GO:0072546(ER membrane protein complex)	NA	*AT5G11560*	catalytics
GH_D06G1034	GO:0003700(DNA binding transcription factor activity);GO:0005634(nucleus);GO:0006355(regulation of transcription, DNA-templated);GO:0007275(multicellular organism development);GO:0046872(metal ion binding)	NA	*AT4G29000*	Tesmin/TSO1-like CXC domain-containing protein
GH_D06G1036	GO:0008150(biological_process);GO:0009507(chloroplast);GO:0016787(hydrolase activity)	NA	*AT5G25770*	alpha/beta-Hydrolases superfamily protein
GH_D06G1037	GO:0005737(cytoplasm);GO:0031625(ubiquitin protein ligase binding);GO:0061630(ubiquitin protein ligase activity)	NA	*AT5G25760*	peroxin4
GH_D06	NA	NA	-	-

		G1038				
		GH_D06 G1039	GO:0003674(molecular_function);GO:0008150(biological_process)	NA	*AT1G55205*	hypothetical protein
		GH_D06 G1043	GO:0005634(nucleus);GO:0016874(ligase activity)	03050(Proteasome)	*AT5G25590*	DNA ligase (DUF630 and DUF632)
		GH_D06 G1045	GO:0004497(monooxygenase activity);GO:0055114(oxidation-reduction process)	00380(Tryptophan metabolism)	*AT5G25620*	Flavin-binding monooxygenase family protein
		GH_D06 G1046	GO:0003723(RNA binding);GO:0003735(structural constituent of ribosome);GO:0006412(translation);GO:0022625(cytosolic large ribosomal subunit)	03010(Ribosome)	*AT1G15250*	Zinc-binding ribosomal protein family protein
		GH_D06 G1047	GO:0005525(GTP binding);GO:0005737(cytoplasm);GO:0009507(chloroplast);GO:0016787(hydrolase activity)	NA	*AT5G11480*	P-loop containing nucleoside triphosphate hydrolases superfamily protein
		GH_D06 G1049	GO:0004675(transmembrane receptor protein serine/threonine kinase activity);GO:0005524(ATP binding);GO:0005886(plasma membrane);GO:0006468(protein phosphorylation);GO:0007166(cell surface receptor signaling pathway);GO:0016021(integral component of membrane);GO:0016301(kinase activity);GO:0016310(phosphorylation)	NA	*AT4G32710*	Protein kinase superfamily protein
Five QEIs	TM273	*GH_A01 G0459*	NA	NA	-	-
		GH_A01 G0464	GO:0005739(mitochondrion);GO:0005794(Golgi apparatus);GO:0005886(plasma membrane);GO:0009306(protein secretion);GO:0015031(protein transport);GO:0015450(P-P-bond-hydrolysis-driven protein	03060(Protein export);04141(Protein processing in endoplasmic reticulum);04145(Phagosome)	*AT1G29310*	SecY protein transport family protein

	transmembrane transporter activity);GO:0016020(membrane);GO:0016021(integral component of membrane)			
GH_A01 G0465	GO:0009699(phenylpropanoid biosynthetic process);GO:0042349(guiding stereospecific synthesis activity)	00970(Aminoacyl-tRNA biosynthesis)	*AT4G38700*	Disease resistance-responsive (dirigent-like protein) family protein
GH_A01 G0466	GO:0009699(phenylpropanoid biosynthetic process);GO:0042349(guiding stereospecific synthesis activity)	00970(Aminoacyl-tRNA biosynthesis)	*AT2G21110*	Disease resistance-responsive (dirigent-like protein) family protein
GH_A01 G0468	GO:0009699(phenylpropanoid biosynthetic process);GO:0042349(guiding stereospecific synthesis activity)	00970(Aminoacyl-tRNA biosynthesis)	*AT4G38700*	Disease resistance-responsive (dirigent-like protein) family protein
GH_A01 G0470	GO:0003887(DNA-directed DNA polymerase activity);GO:0005634(nucleus);GO:0006271(DNA strand elongation involved in DNA replication)	00230(Purine metabolism);00240(Pyrimidine metabolism);03030(DNA replication);03410(Base excision repair);03420(Nucleotide excision repair);03430(Mismatch repair);03440(Homologous recombination)	*AT1G78650*	DNA-directed DNA polymerase
GH_A01 G0471	GO:0005773(vacuole);GO:0034722(gamma-glutamyl-peptidase activity);GO:0046900(tetrahydrofolylpolyglutamate metabolic process)	00790(Folate biosynthesis)	*AT1G78680*	gamma-glutamyl hydrolase 2
GH_A01 G0472	NA	NA	-	-
GH_A01	NA	NA	-	-

G0476				
GH_A01 G0477	GO:0000723(telomere maintenance);GO:0000784(nuclear chromosome, telomeric region);GO:0003684(damaged DNA binding);GO:0003690(double-stranded DNA binding);GO:0004003(ATP-dependent DNA helicase activity);GO:0006303(double-strand break repair via nonhomologous end joining);GO:0006310(DNA recombination);GO:0032508(DNA duplex unwinding);GO:0042162(telomeric DNA binding);GO:0043564(Ku70:Ku80 complex);GO:0071480(cellular response to gamma radiation);GO:0071481(cellular response to X-ray)	03450(Non-homologous end-joining)	*AT1G16970*	ATP-dependent DNA helicase 2 subunit Ku70-like protein
GH_A01 G0478	GO:0005886(plasma membrane);GO:0016301(kinase activity);GO:0016310(phosphorylation)	NA	*AT5G38260*	Protein kinase superfamily protein
GH_A01 G0480	GO:0003677(DNA binding);GO:0003700(DNA binding transcription factor activity);GO:0005634(nucleus);GO:0006351(transcription, DNA-templated);GO:0006355(regulation of transcription, DNA-templated);GO:0009742(brassinosteroid mediated signaling pathway)	04075(Plant hormone signal transduction)	*AT1G78700*	BES1/BZR1 homolog 4
GH_A01 G0481	GO:0032587(ruffle membrane);GO:0035091(phosphatidylinositol binding);GO:1900027(regulation of ruffle assembly)	NA	*AT1G29800*	RING/FYVE/PHD-type zinc finger family protein
GH_A01 G0483	GO:0006952(defense response);GO:0007165(signal transduction);GO:0016021(integral component of membrane);GO:0016301(kinase activity)	03022(Basal transcription factors)	*AT1G45616*	receptor like protein 6
GH_A01	GO:0005737(cytoplasm);GO:0008152(metabolic	NA	*AT3G30841*	Cofactor-independent

G0486	process);GO:0046537(2,3-bisphosphoglycerate-independent phosphoglycerate mutase activity);GO:0046872(metal ion binding)			phosphoglycerate mutase
GH_A01 G0487	GO:0003674(molecular_function);GO:0005739(mitochondrion);GO:0008150(biological_process)	00790(Folate biosynthesis)	*AT3G14830*	epstein-barr nuclear antigen
GH_A01 G0488	GO:0000373(Group II intron splicing);GO:0003723(RNA binding);GO:0005739(mitochondrion);GO:0008270(zinc ion binding);GO:0009507(chloroplast);GO:0009658(chloroplast organization);GO:0010468(regulation of gene expression);GO:0015979(photosynthesis);GO:0016021(integral component of membrane)	00500(Starch and sucrose metabolism)	*AT1G64810*	APO RNA-binding protein (DUF794)
GH_A01 G0491	NA	NA	-	-
GH_A01 G0493	GO:0003674(molecular_function);GO:0005737(cytoplasm);GO:0008150(biological_process)	NA	*AT1G78780*	pathogenesis-related family protein
GH_A01 G0495	GO:0016757(transferase activity, transferring glycosyl groups);GO:0033577(protein glycosylation in endoplasmic reticulum)	00510(N-Glycan biosynthesis)	*AT1G78800*	UDP-Glycosyltransferase superfamily protein
GH_A01 G0496	GO:0003674(molecular_function);GO:0005739(mitochondrion);GO:0008150(biological_process)	NA	*AT1G16916*	transmembrane protein
GH_A01 G0497	GO:0003677(DNA binding);GO:0003700(DNA binding transcription factor activity);GO:0005515(protein binding);GO:0005634(nucleus);GO:0006355(regulation of transcription, DNA-templated);GO:0009944(polarity specification of adaxial/abaxial axis);GO:0045165(cell fate commitment);GO:0046872(metal ion binding);GO:1902183(regulation of shoot apical meristem	NA	*AT2G26580*	plant-specific transcription factor YABBY family protein

		development);GO:2000024(regulation of leaf development)			
	GH_A01 G0498	GO:0005929(cilium);GO:0035091(phosphatidylinositol binding);GO:0061512(protein localization to cilium)	NA	*AT1G53320*	tubby like protein 7
	GH_A01 G0499	GO:0003677(DNA binding);GO:0003713(transcription coactivator activity);GO:0003729(mRNA binding);GO:0005634(nucleus);GO:0005730(nucleolus);GO:0006351(transcription, DNA-templated);GO:0009723(response to ethylene);GO:0043565(sequence-specific DNA binding);GO:0045893(positive regulation of transcription, DNA-templated)	NA	*AT2G42680*	multiprotein bridging factor 1A
	GH_A01 G0500	GO:0003674(molecular_function);GO:0005634(nucleus);GO:0008150(biological_process)	NA	*AT1G78810*	hypothetical protein
	GH_A01 G0501	GO:0003690(double-stranded DNA binding);GO:0046403(polynucleotide 3'-phosphatase activity);GO:0046404(ATP-dependent polydeoxyribonucleotide 5'-hydroxyl-kinase activity);GO:0098504(DNA 3' dephosphorylation involved in DNA repair);GO:0098506(polynucleotide 3' dephosphorylation)	NA	*AT3G14890*	phosphoesterase
	GH_A01 G0502	GO:0003674(molecular_function);GO:0005737(cytoplasm)	NA	*AT3G14910*	Rab3 GTPase-activating protein non-catalytic subunit
TM9997	*GH_A05 G0162*	GO:0005783(endoplasmic reticulum)	NA	*AT5G20150*	SPX domain-containing protein 1
	GH_A05 G0164	GO:0004392(heme oxygenase (decyclizing) activity);GO:0005515(protein binding);GO:0006788(heme oxidation);GO:0009507(chloroplast);GO:0009585(red, far-red light phototransduction);GO:0009813(flavonoid biosynthetic	00860(Porphyrin and chlorophyll metabolism)	*AT2G26670*	Plant heme oxygenase (decyclizing) family protein

	process);GO:0010019(chloroplast-nucleus signaling pathway);GO:0010024(phytochromobilin biosynthetic process);GO:0010075(regulation of meristem growth);GO:0010119(regulation of stomatal movement);GO:0015979(photosynthesis);GO:0016117(carotenoid biosynthetic process);GO:0020037(heme binding);GO:0046872(metal ion binding);GO:0071494(cellular response to UV-C)			
GH_A05G0165	GO:0042214(terpene metabolic process);GO:0052579((+)-pulegone reductase, (+)-isomenthone as substrate, activity);GO:0052580((+)-pulegone reductase, (-)-menthone as substrate, activity);GO:0070402(NADPH binding)	NA	*AT5G16990*	Zinc-binding dehydrogenase family protein
GH_A05G0166	GO:0005768(endosome);GO:0005794(Golgi apparatus);GO:0005802(trans-Golgi network);GO:0005886(plasma membrane);GO:0008514(organic anion transmembrane transporter activity);GO:0008643(carbohydrate transport);GO:0016021(integral component of membrane)	NA	*AT4G32390*	Nucleotide-sugar transporter family protein
GH_A05G0167	GO:0000723(telomere maintenance);GO:0000781(chromosome, telomeric region);GO:0003677(DNA binding);GO:0005515(protein binding);GO:0005634(nucleus)	NA	*AT4G09680*	conserved telomere maintenance component 1
GH_A05G0169	GO:0000786(nucleosome);GO:0003677(DNA binding);GO:0005634(nucleus);GO:0006334(nucleosome assembly);GO:0031492(nucleosomal DNA binding)	NA	*AT5G65360*	Histone superfamily protein
GH_A05G0171	GO:0000055(ribosomal large subunit export from nucleus);GO:0000447(endonucleolytic cleavage in ITS1 to	NA	*AT2G37990*	ribosome biogenesis regulatory protein (RRS1) family protein

	separate SSU-rRNA from 5.8S rRNA and LSU-rRNA from tricistronic rRNA transcript (SSU-rRNA, 5.8S rRNA, LSU-rRNA));GO:0005730(nucleolus);GO:0030687(preribosome, large subunit precursor);GO:0042273(ribosomal large subunit biogenesis)			
GH_A05G0172	GO:0003729(mRNA binding);GO:0005634(nucleus);GO:0005829(cytosol);GO:0005886(plasma membrane);GO:0008150(biological_process)	NA	*AT2G34160*	Alba DNA/RNA-binding protein
GH_A05G0174	GO:0003674(molecular_function);GO:0005575(cellular_component);GO:0008150(biological_process)	NA	*AT1G15400*	hypothetical protein
GH_A05G0175	GO:0003777(microtubule motor activity);GO:0005737(cytoplasm);GO:0005868(cytoplasmic dynein complex);GO:0005875(microtubule associated complex);GO:0007017(microtubule-based process);GO:0030286(dynein complex)	NA	*AT5G20110*	Dynein light chain type 1 family protein
GH_A05G0176	GO:0004462(lactoylglutathione lyase activity);GO:0005737(cytoplasm);GO:0005975(carbohydrate metabolic process);GO:0016829(lyase activity)	NA	*AT1G80160*	Lactoylglutathione lyase / glyoxalase I family protein
GH_A05G0177	GO:0003674(molecular_function);GO:0008150(biological_process);GO:0009507(chloroplast);GO:0016021(integral component of membrane)	NA	*AT2G30300*	Major facilitator superfamily protein
GH_A05G0179	GO:0009252(peptidoglycan biosynthetic process);GO:0047661(amino-acid racemase activity)	NA	*AT1G15410*	aspartate-glutamate racemase family
GH_A05G0181	GO:0003674(molecular_function);GO:0005739(mitochondrion);GO:0016021(integral component of membrane)	NA	*AT5G25940*	early nodulin-like protein
GH_A05	GO:0003674(molecular_function);GO:0005739(mitochondrion);G	NA	*AT5G25940*	early nodulin-like protein

G0182	O:0016021(integral component of membrane)			
GH_A05 G0183	GO:0004674(protein serine/threonine kinase activity);GO:0005634(nucleus);GO:0005737(cytoplasm);GO:0005886(plasma membrane);GO:0035556(intracellular signal transduction)	NA	*AT2G26700*	AGC (cAMP-dependent%2C cGMP-dependent and protein kinase C) kinase family protein
GH_A05 G0186	GO:0005634(nucleus);GO:0016301(kinase activity);GO:0016310(phosphorylation)	00511(Other glycan degradation)	*AT3G25910*	MAP kinase kinase kinase%2C putative (DUF1644)
GH_A05 G0187	GO:0003676(nucleic acid binding);GO:0003964(RNA-directed DNA polymerase activity);GO:0005739(mitochondrion);GO:0008150(biological_process)	NA	*AT2G02650*	Ribonuclease H-like superfamily protein
GH_A05 G0189	GO:0002084(protein depalmitoylation);GO:0005737(cytoplasm);GO:0008474(palmitoyl-(protein) hydrolase activity);GO:0052689(carboxylic ester hydrolase activity)	00564(Glycerophospholipid metabolism)	*AT5G20060*	alpha/beta-Hydrolases superfamily protein
GH_A05 G0190	GO:0003677(DNA binding);GO:0003700(DNA binding transcription factor activity);GO:0005634(nucleus);GO:0006351(transcription, DNA-templated);GO:0006355(regulation of transcription, DNA-templated);GO:0009873(ethylene-activated signaling pathway)	NA	*AT1G50680*	AP2/B3 transcription factor family protein
GH_A05 G0191	GO:0003677(DNA binding);GO:0003700(DNA binding transcription factor activity);GO:0005634(nucleus);GO:0006351(transcription, DNA-templated);GO:0006355(regulation of transcription, DNA-templated);GO:0009873(ethylene-activated signaling	NA	*AT1G51120*	AP2/B3 transcription factor family protein

	pathway)			
GH_A05G0192	GO:0005829(cytosol);GO:0006355(regulation of transcription, DNA-templated);GO:0008469(histone-arginine N-methyltransferase activity);GO:0035242(protein-arginine omega-N asymmetric methyltransferase activity)	NA	*AT1G04870*	protein arginine methyltransferase 10
GH_A05G0193	GO:0004497(monooxygenase activity);GO:0010268(brassinosteroid homeostasis);GO:0016131(brassinosteroid metabolic process)	00905(Brassinosteroid biosynthesis)	*AT2G26710*	Cytochrome P450 superfamily protein
GH_A05G0194	GO:0008270(zinc ion binding);GO:0016021(integral component of membrane)	NA	*AT1G11020*	RING/FYVE/PHD zinc finger superfamily protein
GH_A05G0197	GO:0005634(nucleus);GO:0006633(fatty acid biosynthetic process);GO:0009409(response to cold);GO:0009416(response to light stimulus);GO:0016021(integral component of membrane);GO:0102336(3-oxo-arachidoyl-CoA synthase activity);GO:0102337(3-oxo-cerotoyl-CoA synthase activity);GO:0102338(3-oxo-lignoceronyl-CoA synthase activity);GO:0102756(very-long-chain 3-ketoacyl-CoA synthase activity)	00062(Fatty acid elongation)	*AT2G26640*	3-ketoacyl-CoA synthase 11
GH_A05G0198	NA	NA	*AT5G20140*	SOUL heme-binding family protein
GH_A05G0199	NA	NA	-	-
GH_A05G0201	NA	NA	-	-
GH_A05G0202	NA	NA	-	-

GH_A05G0204	GO:0000139(Golgi membrane);GO:0005457(GDP-fucose transmembrane transporter activity);GO:0005794(Golgi apparatus);GO:0005886(plasma membrane);GO:0008643(carbohydrate transport);GO:0015297(antiporter activity);GO:0015780(nucleotide-sugar transmembrane transport);GO:0016020(membrane);GO:0016021(integral component of membrane);GO:0055085(transmembrane transport)	NA	*AT5G19980*	golgi nucleotide sugar transporter 4
GH_A05G0205	GO:0003700(DNA binding transcription factor activity);GO:0005634(nucleus);GO:0006355(regulation of transcription, DNA-templated);GO:0009723(response to ethylene);GO:0009737(response to abscisic acid);GO:0009863(salicylic acid mediated signaling pathway);GO:0009867(jasmonic acid mediated signaling pathway);GO:0009938(negative regulation of gibberellic acid mediated signaling pathway);GO:0010187(negative regulation of seed germination);GO:0042538(hyperosmotic salinity response);GO:0043565(sequence-specific DNA binding);GO:2000033(regulation of seed dormancy process);GO:2000377(regulation of reactive oxygen species metabolic process)	04075(Plant hormone signal transduction)	*AT1G14920*	GRAS family transcription factor family protein
GH_A05G0208	GO:0003723(RNA binding);GO:0005634(nucleus);GO:0008150(biological_process)	03040(Spliceosome)	*AT5G19960*	RNA-binding (RRM/RBD/RNP motifs) family protein
GH_A05G0209	GO:0003677(DNA binding);GO:0003700(DNA binding transcription factor activity);GO:0005515(protein binding);GO:0005634(nucleus);GO:0006351(transcription,	04933(AGE-RAGE signaling pathway in diabetic complications)	*AT2G03500*	Homeodomain-like superfamily protein

	DNA-templated);GO:0006355(regulation of transcription, DNA-templated);GO:0009266(response to temperature stimulus);GO:0009740(gibberellic acid mediated signaling pathway);GO:0009908(flower development);GO:0010452(histone H3-K36 methylation);GO:0048579(negative regulation of long-day photoperiodism, flowering);GO:1903507(negative regulation of nucleic acid-templated transcription)			
GH_A05 G0210	GO:0003723(RNA binding);GO:0004519(endonuclease activity);GO:0009507(chloroplast);GO:0016556(mRNA modification);GO:0043231(intracellular membrane-bounded organelle)	NA	*AT5G55740*	Tetratricopeptide repeat (TPR)-like superfamily protein
GH_A05 G0211	GO:0004672(protein kinase activity);GO:0004674(protein serine/threonine kinase activity);GO:0005524(ATP binding);GO:0005886(plasma membrane);GO:0006468(protein phosphorylation);GO:0007169(transmembrane receptor protein tyrosine kinase signaling pathway);GO:0016021(integral component of membrane)	NA	*AT2G26730*	Leucine-rich repeat protein kinase family protein
GH_A05 G0214	GO:0000159(protein phosphatase type 2A complex);GO:0005737(cytoplasm);GO:0007165(signal transduction);GO:0019888(protein phosphatase regulator activity)	03015(mRNA surveillance pathway)	*AT5G25510*	Protein phosphatase 2A regulatory B subunit family protein
GH_A05 G0215	GO:0005634(nucleus);GO:0005770(late endosome);GO:0005773(vacuole);GO:0005774(vacuolar membrane);GO:0005783(endoplasmic reticulum);GO:0005794(Golgi apparatus);GO:0005802(trans-Golgi network);GO:0005886(plasma membrane);GO:0006457(protein	NA	*AT2G26890*	DNAJ heat shock N-terminal domain-containing protein

	folding);GO:0006623(protein targeting to vacuole);GO:0006897(endocytosis);GO:0006898(receptor-mediated endocytosis);GO:0007032(endosome organization);GO:0007033(vacuole organization);GO:0009660(amyloplast organization);GO:0009793(embryo development ending in seed dormancy);GO:0009958(positive gravitropism);GO:0009959(negative gravitropism);GO:0010008(endosome membrane);GO:0042594(response to starvation);GO:0043231(intracellular membrane-bounded organelle);GO:0045022(early endosome to late endosome transport);GO:0051301(cell division)			
GH_A05G0216	GO:0003674(molecular_function);GO:0005634(nucleus);GO:0005829(cytosol);GO:0008150(biological_process)	NA	*AT4G13370*	serine/arginine repetitive matrix protein%2C putative (DUF936)
GH_A05G0217	GO:0016787(hydrolase activity)	NA	*AT3G24420*	alpha/beta-Hydrolases superfamily protein
GH_A05G0219	GO:0005524(ATP binding);GO:0009507(chloroplast);GO:0009570(chloroplast stroma);GO:0016226(iron-sulfur cluster assembly);GO:0046872(metal ion binding);GO:0051536(iron-sulfur cluster binding)	NA	*AT3G24430*	ATP binding protein
GH_A05G0220	GO:0005515(protein binding);GO:0005634(nucleus);GO:0005677(chromatin silencing complex);GO:0006351(transcription, DNA-templated);GO:0006355(regulation of transcription,	NA	*AT3G24440*	Fibronectin type III domain-containing protein

	DNA-templated);GO:0009409(response to cold);GO:0009908(flower development);GO:0009909(regulation of flower development);GO:0010048(vernalization response);GO:0016571(histone methylation);GO:0016607(nuclear speck);GO:0031062(positive regulation of histone methylation);GO:0045814(negative regulation of gene expression, epigenetic);GO:0046872(metal ion binding);GO:0048572(short-day photoperiodism);GO:0048575(short-day photoperiodism, flowering);GO:0051571(positive regulation of histone H3-K4 methylation);GO:0061087(positive regulation of histone H3-K27 methylation)			
GH_A05G0221	GO:0003674(molecular_function);GO:0008150(biological_process);GO:0009507(chloroplast)	NA	*AT5G19590*	DUF538 family protein (Protein of unknown function%2C DUF538)
GH_A05G0222	GO:0005737(cytoplasm);GO:0030001(metal ion transport);GO:0046914(transition metal ion binding);GO:0046916(cellular transition metal ion homeostasis)	03013(RNA transport)	*AT3G24450*	Heavy metal transport/detoxification superfamily protein
GH_A05G0226	GO:0005887(integral component of plasma membrane);GO:0015116(sulfate transmembrane transporter activity)	NA	*AT5G19600*	sulfate transporter 3%3B5
GH_A05G0227	GO:0005086(ARF guanyl-nucleotide exchange factor activity);GO:0005802(trans-Golgi network);GO:0016192(vesicle-mediated transport)	04144(Endocytosis)	*AT5G19610*	GNOM-like 2
GH_A05G0228	GO:0004029(aldehyde dehydrogenase (NAD) activity)	00940(Phenylpropanoid biosynthesis)	*AT3G24503*	aldehyde dehydrogenase 2C4

GH_A05 G0229	GO:0004674(protein serine/threonine kinase activity);GO:0005634(nucleus);GO:0005737(cytoplasm);GO:0006897(endocytosis);GO:0008360(regulation of cell shape);GO:0016055(Wnt signaling pathway);GO:0018105(peptidyl-serine phosphorylation)	NA	*AT3G03940*	Protein kinase family protein
GH_A05 G0230	GO:0005886(plasma membrane);GO:0008150(biological_process);GO:0031225(anchored component of membrane)	NA	*AT5G53250*	arabinogalactan protein 22
GH_A05 G0231	GO:0005768(endosome);GO:0030139(endocytic vesicle)	04144(Endocytosis);04145(Phagosome)	*AT5G45130*	Ras small GTP-binding family protein
GH_A05 G0232	GO:0003674(molecular_function);GO:0005634(nucleus);GO:0008150(biological_process);GO:0009507(chloroplast)	NA	*AT3G24506*	hypothetical protein
GH_A05 G0233	GO:0003777(microtubule motor activity);GO:0005871(kinesin complex);GO:0007018(microtubule-based movement);GO:0016887(ATPase activity)	NA	*AT3G16060*	ATP binding microtubule motor family protein
GH_A05 G0234	GO:0003677(DNA binding);GO:0003700(DNA binding transcription factor activity);GO:0005515(protein binding);GO:0005634(nucleus);GO:0006351(transcription, DNA-templated);GO:0006355(regulation of transcription, DNA-templated);GO:0043565(sequence-specific DNA binding);GO:0061408(positive regulation of transcription from RNA polymerase II promoter in response to heat stress)	NA	*AT3G24520*	heat shock transcription factor C1
GH_A05 G0235	GO:0004497(monooxygenase activity);GO:0055114(oxidation-reduction process)	00380(Tryptophan metabolism)	*AT4G13260*	Flavin-binding monooxygenase family protein
GH_A05 G0236	GO:0005515(protein binding);GO:0005634(nucleus);GO:0006351(transcription,	00970(Aminoacyl-tRNA biosynthesis)	*AT5G19650*	ovate family protein 8

	DNA-templated);GO:0045892(negative regulation of transcription, DNA-templated)			
GH_A05G0237	GO:0010304(PSII associated light-harvesting complex II catabolic process);GO:0015996(chlorophyll catabolic process);GO:0034256(chlorophyll(ide) b reductase activity)	00860(Porphyrin and chlorophyll metabolism)	*AT4G13250*	NAD(P)-binding Rossmann-fold superfamily protein
GH_A05G0239	GO:0004252(serine-type endopeptidase activity);GO:0005618(cell wall)	04141(Protein processing in endoplasmic reticulum)	*AT5G19660*	SITE-1 protease
GH_A05G0240	GO:0005737(cytoplasm);GO:0009507(chloroplast);GO:0009658(chloroplast organization);GO:0009793(embryo development ending in seed dormancy);GO:0010098(suspensor development)	NA	*AT3G24560*	Adenine nucleotide alpha hydrolases-like superfamily protein
GH_A05G0241	GO:0003674(molecular_function);GO:0005739(mitochondrion);GO:0005778(peroxisomal membrane);GO:0008150(biological_process);GO:0016021(integral component of membrane)	04146(Peroxisome)	*AT3G24570*	Peroxisomal membrane 22 kDa (Mpv17/PMP22) family protein
GH_A05G0242	GO:0004252(serine-type endopeptidase activity);GO:0006465(signal peptide processing);GO:0006627(protein processing involved in protein targeting to mitochondrion);GO:0009535(chloroplast thylakoid membrane);GO:0010027(thylakoid membrane organization);GO:0033108(mitochondrial respiratory chain complex assembly);GO:0042720(mitochondrial inner membrane peptidase complex)	03060(Protein export)	*AT3G24590*	plastidic type i signal peptidase 1
GH_A05G0243	GO:0016021(integral component of membrane)	NA	*AT3G24605*	late embryogenesis abundant protein%2C group 2
GH_A05G0244	GO:0003674(molecular_function);GO:0005634(nucleus);GO:0005794(Golgi apparatus);GO:0009734(auxin-activated signaling	NA	*AT2G20120*	CONTINUOUS VASCULAR RING protein (DUF502)

	pathway);GO:0010222(stem vascular tissue pattern formation);GO:0016021(integral component of membrane)			
GH_A05 G0246	GO:0005351(carbohydrate:proton symporter activity);GO:0008643(carbohydrate transport);GO:0015144(carbohydrate transmembrane transporter activity);GO:0016020(membrane);GO:0016021(integral component of membrane);GO:0022857(transmembrane transporter activity);GO:0031969(chloroplast membrane);GO:0055085(transmembrane transport)	NA	*AT1G67300*	Major facilitator superfamily protein
GH_A05 G0248	GO:0003924(GTPase activity);GO:0005525(GTP binding);GO:0005576(extracellular region);GO:0005622(intracellular);GO:0005886(plasma membrane);GO:0007264(small GTPase mediated signal transduction);GO:0009738(abscisic acid-activated signaling pathway)	04145(Phagosome);04933(AGE-RAGE signaling pathway in diabetic complications)	*AT4G28950*	RHO-related protein from plants 9
GH_A05 G0259	GO:0005634(nucleus);GO:0005737(cytoplasm);GO:0006417(regulation of translation);GO:0016763(transferase activity, transferring pentosyl groups);GO:0019988(charged-tRNA amino acid modification);GO:0043399(tRNA A64-2'-O-ribosylphosphate transferase activity)	NA	*AT2G40570*	initiator tRNA phosphoribosyl transferase family protein
GH_A05 G0260	GO:0006679(glucosylceramide biosynthetic process);GO:0008120(ceramide glucosyltransferase activity);GO:0016020(membrane)	NA	*AT2G19880*	Nucleotide-diphospho-sugar transferases superfamily protein
GH_A05 G0261	GO:0004674(protein serine/threonine kinase activity);GO:0005524(ATP binding);GO:0005886(plasma membrane);GO:0006468(protein	NA	*AT5G25930*	kinase family with leucine-rich repeat domain-containing protein

	phosphorylation);GO:0016020(membrane);GO:0016021(integral component of membrane);GO:0016301(kinase activity)			
GH_A05G0262	GO:0006855(drug transmembrane transport)	NA	*AT4G29140*	MATE efflux family protein
GH_A05G0263	GO:0005516(calmodulin binding);GO:0005794(Golgi apparatus);GO:0008150(biological_process)	NA	*AT3G16490*	IQ-domain 26
GH_A05G0264	GO:0004674(protein serine/threonine kinase activity);GO:0005643(nuclear pore);GO:0005770(late endosome);GO:0006468(protein phosphorylation);GO:0006623(protein targeting to vacuole);GO:0016236(macroautophagy);GO:0030242(autophagy of peroxisome);GO:0034271(phosphatidylinositol 3-kinase complex, class III, type I);GO:0034272(phosphatidylinositol 3-kinase complex, class III, type II);GO:0045324(late endosome to vacuole transport);GO:0071561(nucleus-vacuole junction)	04136(Autophagy - other eukaryotes)	*AT4G29380*	protein kinase family protein / WD-40 repeat family protein
GH_A05G0268	GO:0004553(hydrolase activity, hydrolyzing O-glycosyl compounds);GO:0005576(extracellular region);GO:0005886(plasma membrane);GO:0005975(carbohydrate metabolic process);GO:0006952(defense response);GO:0009505(plant-type cell wall);GO:0031225(anchored component of membrane);GO:0042973(glucan endo-1,3-beta-D-glucosidase activity);GO:0046658(anchored component of plasma membrane);GO:0071555(cell wall organization)	00500(Starch and sucrose metabolism)	*AT5G56590*	O-Glycosyl hydrolases family 17 protein
GH_A05G0269	GO:0003785(actin monomer binding);GO:0005938(cell cortex);GO:0015629(actin	NA	*AT5G56600*	profilin

	cytoskeleton);GO:0042989(sequestering of actin monomers)			
GH_A05G0270	GO:0003785(actin monomer binding);GO:0005938(cell cortex);GO:0015629(actin cytoskeleton);GO:0042989(sequestering of actin monomers)	NA	*AT2G19770*	profilin 5
GH_A05G0271	GO:0003674(molecular_function);GO:0005634(nucleus);GO:0008150(biological_process);GO:0009505(plant-type cell wall)	NA	*AT4G29310*	DUF1005 family protein (DUF1005)
GH_A05G0273	GO:0004439(phosphatidylinositol-4,5-bisphosphate 5-phosphatase activity)	NA	*AT5G56610*	Phosphotyrosine protein phosphatases superfamily protein
GH_A05G0274	GO:0003677(DNA binding);GO:0003700(DNA binding transcription factor activity);GO:0005634(nucleus);GO:0006351(transcription, DNA-templated);GO:0006355(regulation of transcription, DNA-templated);GO:0007275(multicellular organism development);GO:0044212(transcription regulatory region DNA binding);GO:0045893(positive regulation of transcription, DNA-templated);GO:0071555(cell wall organization);GO:1905177(tracheary element differentiation)	NA	*AT4G29230*	NAC domain containing protein 75
GH_A05G0276	GO:0003872(6-phosphofructokinase activity);GO:0005524(ATP binding);GO:0005737(cytoplasm);GO:0005829(cytosol);GO:0005945(6-phosphofructokinase complex);GO:0006002(fructose 6-phosphate metabolic process);GO:0006096(glycolytic process);GO:0010053(root epidermal cell differentiation);GO:0046872(metal ion binding)	00010(Glycolysis / Gluconeogenesis);00030(Pentose phosphate pathway);00051(Fructose and mannose metabolism);00052(Galactose metabolism);03018(RNA degradation)	*AT4G26270*	phosphofructokinase 3
GH_A05	GO:0005515(protein binding);GO:0005794(Golgi	NA	*AT2G19790*	SNARE-like superfamily protein

G0277	apparatus);GO:0005802(trans-Golgi network);GO:0005905(clathrin-coated pit);GO:0006886(intracellular protein transport);GO:0015031(protein transport);GO:0016192(vesicle-mediated transport);GO:0030124(AP-4 adaptor complex);GO:0030125(clathrin vesicle coat)			
GH_A05 G0278	GO:0003676(nucleic acid binding);GO:0003677(DNA binding);GO:0003700(DNA binding transcription factor activity);GO:0005634(nucleus);GO:0005886(plasma membrane);GO:0006355(regulation of transcription, DNA-templated);GO:0006979(response to oxidative stress);GO:0046872(metal ion binding)	NA	*AT4G29190*	Zinc finger C-x8-C-x5-C-x3-H type family protein
GH_A05 G0282	GO:0002181(cytoplasmic translation);GO:0003735(structural constituent of ribosome);GO:0022625(cytosolic large ribosomal subunit)	03010(Ribosome)	*AT5G56710*	Ribosomal protein L31e family protein
GH_A05 G0283	GO:0004222(metalloendopeptidase activity);GO:0005739(mitochondrion);GO:0008270(zinc ion binding);GO:0016485(protein processing);GO:0043171(peptide catabolic process);GO:0051603(proteolysis involved in cellular protein catabolic process)	NA	*AT5G56730*	Insulinase (Peptidase family M16) protein
GH_A05 G0284	GO:0003735(structural constituent of ribosome);GO:0005739(mitochondrion);GO:0005773(vacuole);GO:0006412(translation);GO:0009507(chloroplast);GO:0022626(cytosolic ribosome);GO:0022627(cytosolic small ribosomal subunit)	03010(Ribosome)	*AT4G29430*	ribosomal protein S15A E
GH_A05	GO:0005515(protein	00270(Cysteine and methionine	*AT4G26200*	1-amino-cyclopropane-1-carboxyl

	G0288	binding);GO:0005737(cytoplasm);GO:0009693(ethylene biosynthetic process);GO:0009835(fruit ripening);GO:0016847(1-aminocyclopropane-1-carboxylate synthase activity);GO:0030170(pyridoxal phosphate binding);GO:0042802(identical protein binding)	metabolism)		ate synthase 7
TM13308	*GH_A05G4023*	GO:0003333(amino acid transmembrane transport);GO:0005886(plasma membrane);GO:0015171(amino acid transmembrane transporter activity)	NA	*AT5G09220*	amino acid permease 2
	GH_A05G4025	GO:0004565(beta-galactosidase activity);GO:0005618(cell wall);GO:0005773(vacuole)	00052(Galactose metabolism);00600(Sphingolipid metabolism);00531(Glycosaminoglycan degradation);00604(Glycosphingolipid biosynthesis - ganglio series);00511(Other glycan degradation)	*AT5G63800*	Glycosyl hydrolase family 35 protein
	GH_A05G4026	GO:0003677(DNA binding);GO:0005515(protein binding);GO:0005634(nucleus);GO:0006351(transcription, DNA-templated);GO:0007275(multicellular organism development);GO:0008361(regulation of cell size);GO:0009416(response to light stimulus);GO:0009611(response to wounding);GO:0009620(response to fungus);GO:0009744(response to sucrose);GO:0009751(response to salicylic acid);GO:0009753(response to jasmonic acid);GO:0009793(embryo development ending in seed	NA	*AT5G08790*	NAC (No Apical Meristem) domain transcriptional regulator superfamily protein

	dormancy);GO:0010099(regulation of photomorphogenesis);GO:0010150(leaf senescence);GO:0016032(viral process);GO:0043424(protein histidine kinase binding);GO:0045892(negative regulation of transcription, DNA-templated);GO:0045893(positive regulation of transcription, DNA-templated)			
GH_A05G4028	GO:0003674(molecular_function);GO:0005739(mitochondrion);GO:0008150(biological_process)	NA	*AT5G08770*	topoisomerase I damage affected-like protein
GH_A05G4029	GO:0005634(nucleus);GO:0008270(zinc ion binding)	NA	*AT5G08750*	RING/FYVE/PHD zinc finger superfamily protein
GH_A05G4032	GO:0003674(molecular_function);GO:0009733(response to auxin)	04075(Plant hormone signal transduction)	*AT5G53590*	SAUR-like auxin-responsive protein family
GH_A05G4039	GO:0003677(DNA binding);GO:0003700(DNA binding transcription factor activity);GO:0005634(nucleus);GO:0006351(transcription, DNA-templated);GO:0006355(regulation of transcription, DNA-templated);GO:0007275(multicellular organism development);GO:0010089(xylem development);GO:0043067(regulation of programmed cell death);GO:0048367(shoot system development)	NA	*AT5G64530*	xylem NAC domain 1
GH_A05G4040	GO:0003863(3-methyl-2-oxobutanoate dehydrogenase (2-methylpropanoyl-transferring) activity);GO:0005739(mitochondrion);GO:0005759(mitochondrial matrix);GO:0016624(oxidoreductase activity, acting on the aldehyde or oxo group of donors, disulfide as acceptor);GO:0046872(metal ion	00640(Propanoate metabolism);00280(Valine, leucine and isoleucine degradation)	*AT5G09300*	Thiamin diphosphate-binding fold (THDP-binding) superfamily protein

	binding);GO:0055114(oxidation-reduction process)			
GH_A05G4041	GO:0005451(monovalent cation:proton antiporter activity);GO:0006885(regulation of pH);GO:0012505(endomembrane system)	NA	*AT2G13620*	cation/hydrogen exchanger 15
GH_A05G4044	GO:0004842(ubiquitin-protein transferase activity);GO:0005737(cytoplasm);GO:0016567(protein ubiquitination)	04120(Ubiquitin mediated proteolysis)	*AT3G19380*	plant U-box 25
GH_A05G4045	GO:0003924(GTPase activity);GO:0005525(GTP binding);GO:0005794(Golgi apparatus);GO:0005886(plasma membrane);GO:0015031(protein transport)	NA	*AT1G43890*	RAB GTPASE HOMOLOG B18
GH_A05G4048	GO:0003674(molecular_function);GO:0005768(endosome);GO:0005794(Golgi apparatus);GO:0005802(trans-Golgi network);GO:0008150(biological_process);GO:0009507(chloroplast);GO:0016021(integral component of membrane)	NA	*AT1G31130*	polyadenylate-binding protein 1-B-binding protein
GH_A05G4049	GO:0004650(polygalacturonase activity);GO:0005576(extracellular region);GO:0005975(carbohydrate metabolic process);GO:0016021(integral component of membrane);GO:0016829(lyase activity);GO:0071555(cell wall organization)	00040(Pentose and glucuronate interconversions)	*AT4G33440*	Pectin lyase-like superfamily protein
GH_A05G4050	GO:0070765(gamma-secretase complex)	00195(Photosynthesis)	*AT5G64040*	photosystem I reaction center subunit PSI-N%2C chloroplast%2C putative / PSI-N%2C putative (PSAN)
GH_A05G4055	GO:0005737(cytoplasm)	NA	*AT1G53570*	mitogen-activated protein kinase kinase kinase 3

GH_A05G4056	GO:0005634(nucleus);GO:0016567(protein ubiquitination)	NA	*AT2G13690*	PRLI-interacting factor
GH_A05G4057	GO:0005634(nucleus)	NA	*AT4G33467*	hypothetical protein
GH_A05G4059	GO:0003674(molecular_function);GO:0005634(nucleus);GO:0008150(biological_process);GO:0016021(integral component of membrane)	NA	*AT4G14620*	hypothetical protein (DUF506)
GH_A05G4060	GO:0003735(structural constituent of ribosome);GO:0022625(cytosolic large ribosomal subunit)	03010(Ribosome)	*AT4G15000*	Ribosomal L27e protein family
GH_A05G4065	GO:0004842(ubiquitin-protein transferase activity);GO:0019005(SCF ubiquitin ligase complex);GO:0031146(SCF-dependent proteasomal ubiquitin-dependent protein catabolic process)	NA	*AT1G64290*	F-box protein-like protein
GH_A05G4066	GO:0004674(protein serine/threonine kinase activity);GO:0005886(plasma membrane);GO:0006468(protein phosphorylation)	NA	*AT5G60900*	receptor-like protein kinase 1
GH_A05G4067	GO:0005634(nucleus);GO:0016301(kinase activity);GO:0016310(phosphorylation)	NA	*AT1G49170*	hypothetical protein
GH_A05G4068	GO:0004674(protein serine/threonine kinase activity);GO:0005886(plasma membrane);GO:0006468(protein phosphorylation)	NA	*AT5G60900*	receptor-like protein kinase 1
GH_A05G4069	GO:0004674(protein serine/threonine kinase activity);GO:0005886(plasma membrane);GO:0006468(protein phosphorylation)	NA	*AT5G60900*	receptor-like protein kinase 1
GH_A05G4074	GO:0004674(protein serine/threonine kinase activity);GO:0005886(plasma membrane);GO:0006468(protein	NA	*AT5G60900*	receptor-like protein kinase 1

		phosphorylation)			
TM17169	*GH_A06G1634*	GO:0004190(aspartic-type endopeptidase activity);GO:0006508(proteolysis);GO:0030163(protein catabolic process)	NA	*AT5G45120*	Eukaryotic aspartyl protease family protein
	GH_A06G1635	GO:0003729(mRNA binding);GO:0005515(protein binding);GO:0005634(nucleus);GO:0005737(cytoplasm);GO:0005829(cytosol);GO:0006351(transcription, DNA-templated);GO:0006355(regulation of transcription, DNA-templated);GO:0006417(regulation of translation);GO:0019048(modulation by virus of host morphology or physiology);GO:0031047(gene silencing by RNA);GO:0035197(siRNA binding);GO:0042742(defense response to bacterium);GO:0051607(defense response to virus)	NA	*AT1G31280*	Argonaute family protein
	GH_A06G1655	GO:0003674(molecular_function);GO:0005737(cytoplasm);GO:0007275(multicellular organism development);GO:0009733(response to auxin);GO:0009734(auxin-activated signaling pathway);GO:0040008(regulation of growth)	04075(Plant hormone signal transduction)	*AT1G56150*	SAUR-like auxin-responsive protein family
	GH_A06G1656	GO:0003674(molecular_function);GO:0005739(mitochondrion);GO:0008150(biological_process)	NA	*AT1G79660*	ephrin-A3 protein
	GH_A06G1657	GO:0015194(L-serine transmembrane transporter activity);GO:0016021(integral component of membrane)	NA	*AT1G16180*	Serinc-domain containing serine and sphingolipid biosynthesis protein
	GH_A06G1658	GO:0003684(damaged DNA binding);GO:0005515(protein binding);GO:0005634(nucleus);GO:0005737(cytoplasm);GO:0006284(base-excision repair);GO:0006289(nucleotide-excision repair);GO:0031593(polyubiquitin modification-dependent protein	04141(Protein processing in endoplasmic reticulum);03420(Nucleotide excision repair)	*AT1G79650*	Rad23 UV excision repair protein family

		binding);GO:0043130(ubiquitin binding);GO:0043161(proteasome-mediated ubiquitin-dependent protein catabolic process);GO:0070628(proteasome binding)			
	GH_A06 G1660	GO:0003674(molecular_function);GO:0005886(plasma membrane);GO:0031225(anchored component of membrane)	NA	*AT3G12660*	FASCICLIN-like arabinogalactan protein 14 precursor
	GH_A06 G1661	GO:0004034(aldose 1-epimerase activity);GO:0005829(cytosol);GO:0005975(carbohydrate metabolic process);GO:0006012(galactose metabolic process);GO:0009737(response to abscisic acid);GO:0047938(glucose-6-phosphate 1-epimerase activity)	00010(Glycolysis / Gluconeogenesis)	*AT5G57330*	Galactose mutarotase-like superfamily protein
	GH_A06 G1662	GO:0003674(molecular_function);GO:0005575(cellular_component);GO:0048367(shoot system development)	NA	*AT1G53708*	ROTUNDIFOLIA like 9
	GH_A06 G1663	GO:0004672(protein kinase activity);GO:0004674(protein serine/threonine kinase activity);GO:0004675(transmembrane receptor protein serine/threonine kinase activity);GO:0005524(ATP binding);GO:0005576(extracellular region);GO:0005886(plasma membrane);GO:0006468(protein phosphorylation);GO:0007166(cell surface receptor signaling pathway);GO:0007169(transmembrane receptor protein tyrosine kinase signaling pathway);GO:0016021(integral component of membrane)	NA	*AT1G53730*	STRUBBELIG-receptor family 6
	GH_A06 G1665	GO:0046922(peptide-O-fucosyltransferase activity)	00514(Other types of O-glycan biosynthesis)	*AT1G17270*	O-fucosyltransferase family protein
TM80413	*GH_D13 G0562*	GO:0003674(molecular_function);GO:0005774(vacuolar membrane);GO:0005783(endoplasmic reticulum);GO:0005794(Golgi apparatus)	NA	*AT1G36050*	Endoplasmic reticulum vesicle transporter protein

GH_D13G0565	GO:0008150(biological_process);GO:0009507(chloroplast)	03018(RNA degradation)	*AT4G13070*	RNA-binding CRS1 / YhbY (CRM) domain protein
GH_D13G0566	GO:0004672(protein kinase activity);GO:0004674(protein serine/threonine kinase activity);GO:0005524(ATP binding);GO:0006468(protein phosphorylation);GO:0007169(transmembrane receptor protein tyrosine kinase signaling pathway);GO:0016021(integral component of membrane);GO:0016301(kinase activity);GO:0080092(regulation of pollen tube growth)	NA	*AT4G31250*	Leucine-rich repeat protein kinase family protein
GH_D13G0569	GO:0003674(molecular_function);GO:0005737(cytoplasm)	NA	*AT1G22040*	Galactose oxidase/kelch repeat superfamily protein
GH_D13G0572	GO:0004675(transmembrane receptor protein serine/threonine kinase activity);GO:0005515(protein binding);GO:0005524(ATP binding);GO:0005886(plasma membrane);GO:0006468(protein phosphorylation);GO:0007166(cell surface receptor signaling pathway);GO:0008289(lipid binding);GO:0009556(microsporogenesis);GO:0009742(brassinosteroid mediated signaling pathway);GO:0010152(pollen maturation);GO:0016021(integral component of membrane);GO:0016301(kinase activity);GO:0033612(receptor serine/threonine kinase binding)	04016(MAPK signaling pathway - plant);04075(Plant hormone signal transduction);04626(Plant-pathogen interaction)	*AT4G33430*	BRI1-associated receptor kinase
GH_D13G0573	GO:0003674(molecular_function);GO:0005634(nucleus)	NA	*AT5G64550*	loricrin-like protein
GH_D13G0574	GO:0003730(mRNA 3'-UTR binding);GO:0005829(cytosol);GO:0046872(metal ion binding);GO:0061158(3'-UTR-mediated mRNA destabilization)	NA	*AT1G66810*	Zinc finger C-x8-C-x5-C-x3-H type family protein

GH_D13G0576	GO:0005545(1-phosphatidylinositol binding);GO:0005634(nucleus);GO:0005794(Golgi apparatus);GO:0005905(clathrin-coated pit);GO:0006897(endocytosis);GO:0030136(clathrin-coated vesicle);GO:0030276(clathrin binding);GO:0048268(clathrin coat assembly)	NA	*AT1G03050*	ENTH/ANTH/VHS superfamily protein
GH_D13G0578	GO:0004675(transmembrane receptor protein serine/threonine kinase activity);GO:0005524(ATP binding);GO:0005634(nucleus);GO:0005886(plasma membrane);GO:0006468(protein phosphorylation);GO:0006952(defense response);GO:0007166(cell surface receptor signaling pathway);GO:0009507(chloroplast);GO:0016301(kinase activity)	NA	*AT2G17220*	Protein kinase superfamily protein
GH_D13G0579	GO:0003333(amino acid transmembrane transport);GO:0015171(amino acid transmembrane transporter activity)	NA	*AT2G42005*	Transmembrane amino acid transporter family protein
GH_D13G0580	GO:0004683(calmodulin-dependent protein kinase activity);GO:0005516(calmodulin binding);GO:0005634(nucleus);GO:0005737(cytoplasm);GO:0005886(plasma membrane);GO:0009738(abscisic acid-activated signaling pathway);GO:0009931(calcium-dependent protein serine/threonine kinase activity);GO:0018105(peptidyl-serine phosphorylation);GO:0035556(intracellular signal transduction);GO:0046777(protein autophosphorylation)	04626(Plant-pathogen interaction)	*AT2G17290*	Calcium-dependent protein kinase family protein
GH_D13G0583	GO:0005351(carbohydrate:proton symporter activity);GO:0005355(glucose transmembrane transporter	NA	*AT4G35300*	tonoplast monosaccharide transporter2

	activity);GO:0005887(integral component of plasma membrane);GO:0046323(glucose import)			
GH_D13 G0584	GO:0004970(ionotropic glutamate receptor activity);GO:0005217(intracellular ligand-gated ion channel activity);GO:0005576(extracellular region);GO:0006811(ion transport);GO:0006874(cellular calcium ion homeostasis);GO:0006883(cellular sodium ion homeostasis);GO:0009416(response to light stimulus);GO:0016021(integral component of membrane);GO:0030007(cellular potassium ion homeostasis)	NA	*AT4G35290*	glutamate receptor 2
GH_D13 G0586	GO:0000462(maturation of SSU-rRNA from tricistronic rRNA transcript (SSU-rRNA, 5.8S rRNA, LSU-rRNA));GO:0005730(nucleolus);GO:0030692(Noc4p-Nop14p complex);GO:0032040(small-subunit processome)	NA	*AT2G17250*	CCAAT-binding factor
GH_D13 G0588	GO:0004594(pantothenate kinase activity);GO:0005737(cytoplasm);GO:0016301(kinase activity);GO:0016310(phosphorylation)	00770(Pantothenate and CoA biosynthesis)	*AT4G35360*	pantothenate kinase
GH_D13 G0590	GO:0004046(aminoacylase activity);GO:0005737(cytoplasm);GO:0005773(vacuole);GO:0005783(endoplasmic reticulum);GO:0005794(Golgi apparatus);GO:0010043(response to zinc ion)	00220(Arginine biosynthesis)	*AT4G38220*	Peptidase M20/M25/M40 family protein
GH_D13 G0591	NA	NA	*AT4G38213*	tubulin-tyrosine ligase
GH_D13 G0594	GO:0004420(hydroxymethylglutaryl-CoA reductase (NADPH) activity);GO:0005778(peroxisomal membrane);GO:0005789(endoplasmic reticulum	00900(Terpenoid backbone biosynthesis)	*AT1G76490*	hydroxy methylglutaryl CoA reductase 1

	membrane);GO:0008299(isoprenoid biosynthetic process);GO:0016126(sterol biosynthetic process)			
GH_D13G0596	NA	NA	*AT4G38160*	Mitochondrial transcription termination factor family protein
GH_D13G0597	GO:0003674(molecular_function);GO:0005739(mitochondrion);GO:0008150(biological_process)	NA	*AT4G38150*	Pentatricopeptide repeat (PPR) superfamily protein
GH_D13G0599	GO:0016787(hydrolase activity)	NA	*AT5G38520*	alpha/beta-Hydrolases superfamily protein
GH_D13G0601	GO:0004557(alpha-galactosidase activity);GO:0005576(extracellular region);GO:0005975(carbohydrate metabolic process);GO:0009505(plant-type cell wall);GO:0009620(response to fungus);GO:0009911(positive regulation of flower development);GO:0009965(leaf morphogenesis);GO:0048046(apoplast);GO:0052692(raffinose alpha-galactosidase activity);GO:0071555(cell wall organization)	00052(Galactose metabolism);00561(Glycerolipid metabolism);00600(Sphingolipid metabolism);00603(Glycosphingolipid biosynthesis - globo and isoglobo series)	*AT5G08370*	alpha-galactosidase 2
GH_D13G0604	GO:0000287(magnesium ion binding);GO:0004743(pyruvate kinase activity);GO:0005737(cytoplasm);GO:0005829(cytosol);GO:0006096(glycolytic process);GO:0016301(kinase activity);GO:0030955(potassium ion binding)	00010(Glycolysis / Gluconeogenesis);00620(Pyruvate metabolism);00230(Purine metabolism)	*AT5G08570*	Pyruvate kinase family protein
GH_D13G0606	GO:0005929(cilium);GO:0030991(intraciliary transport particle A);GO:0035721(intraciliary retrograde transport);GO:0060271(cilium assembly);GO:0061512(protein localization to cilium)	NA	*AT3G19990*	E3 ubiquitin-protein ligase
GH_D13	GO:0005929(cilium);GO:0030991(intraciliary transport particle	NA	*AT3G19990*	E3 ubiquitin-protein ligase

G0607	A);GO:0035721(intraciliary retrograde transport);GO:0060271(cilium assembly);GO:0061512(protein localization to cilium)			
GH_D13 G0608	GO:0005618(cell wall);GO:0005829(cytosol);GO:0005886(plasma membrane);GO:0006952(defense response);GO:0007165(signal transduction);GO:0009506(plasmodesma)	04016(MAPK signaling pathway - plant);04626(Plant-pathogen interaction)	*AT5G23400*	Leucine-rich repeat (LRR) family protein
GH_D13 G0610	GO:0005829(cytosol);GO:0006355(regulation of transcription, DNA-templated);GO:0008469(histone-arginine N-methyltransferase activity);GO:0035242(protein-arginine omega-N asymmetric methyltransferase activity)	NA	*AT3G20020*	protein arginine methyltransferase 6
GH_D13 G0611	GO:0005509(calcium ion binding);GO:0005783(endoplasmic reticulum);GO:0005794(Golgi apparatus);GO:0005886(plasma membrane);GO:0016021(integral component of membrane)	NA	*AT5G08580*	Calcium-binding EF hand family protein
GH_D13 G0613	GO:0003677(DNA binding);GO:0003700(DNA binding transcription factor activity);GO:0005515(protein binding);GO:0005634(nucleus);GO:0006351(transcription, DNA-templated);GO:0006355(regulation of transcription, DNA-templated);GO:0009414(response to water deprivation);GO:0009651(response to salt stress);GO:0009738(abscisic acid-activated signaling pathway);GO:0009739(response to gibberellin);GO:0009751(response to salicylic acid);GO:0009787(regulation of abscisic acid-activated signaling pathway);GO:0010116(positive regulation of abscisic acid biosynthetic process);GO:0045893(positive regulation of transcription, DNA-templated);GO:1901001(negative regulation of	NA	*AT5G08520*	Duplicated homeodomain-like superfamily protein

			response to salt stress);GO:1901371(regulation of leaf morphogenesis);GO:1905615(positive regulation of developmental vegetative growth)			
Four significant QQIs	TM10542/TM59085	*GH_A05G1290*	GO:0005887(integral component of plasma membrane);GO:0015116(sulfate transmembrane transporter activity)	NA	*AT3G15990*	sulfate transporter 3%3B4
		GH_A05G1318	GO:0001046(core promoter sequence-specific DNA binding);GO:0001228(transcriptional activator activity, RNA polymerase II transcription regulatory region sequence-specific DNA binding);GO:0005634(nucleus);GO:0006366(transcription by RNA polymerase II)	NA	*AT4G29100*	basic helix-loop-helix (bHLH) DNA-binding superfamily protein
		GH_A05G1320	GO:0003899(DNA-directed 5'-3' RNA polymerase activity);GO:0005739(mitochondrion);GO:0005768(endosome);GO:0005794(Golgi apparatus);GO:0005802(trans-Golgi network);GO:0008150(biological_process);GO:0016021(integral component of membrane)	NA	*AT3G16200*	DNA-directed RNA polymerase subunit beta
		GH_A05G1321	GO:0016787(hydrolase activity)	00860(Porphyrin and chlorophyll metabolism)	*AT4G09900*	methyl esterase 12
		GH_A05G1322	GO:0005634(nucleus);GO:0006886(intracellular protein transport);GO:0016020(membrane)	03013(RNA transport)	*AT3G16270*	ENTH/VHS family protein
		GH_A05G1323	GO:0005739(mitochondrion);GO:0009507(chloroplast);GO:0009941(chloroplast envelope)	NA	*AT5G06220*	LETM1-like protein
		GH_A05G1324	GO:0005758(mitochondrial intermembrane space);GO:0009060(aerobic	NA	*AT1G69750*	cytochrome c oxidase 19-2

	respiration);GO:0033617(mitochondrial respiratory chain complex IV assembly)			
GH_A05G1325	GO:0003677(DNA binding);GO:0003700(DNA binding transcription factor activity);GO:0005634(nucleus);GO:0006351(transcription, DNA-templated);GO:0006355(regulation of transcription, DNA-templated);GO:0009873(ethylene-activated signaling pathway);GO:0043565(sequence-specific DNA binding);GO:0045893(positive regulation of transcription, DNA-templated)	NA	*AT5G11590*	Integrase-type DNA-binding superfamily protein
GH_A05G1326	GO:0005515(protein binding);GO:0005524(ATP binding);GO:0005739(mitochondrion);GO:0005794(Golgi apparatus);GO:0005829(cytosol);GO:0005886(plasma membrane);GO:0006457(protein folding);GO:0007005(mitochondrion organization);GO:0008219(cell death);GO:0009409(response to cold);GO:0009507(chloroplast);GO:0009570(chloroplast stroma);GO:0009627(systemic acquired resistance);GO:0009941(chloroplast envelope);GO:0010319(stromule);GO:0016020(membrane);GO:0022626(cytosolic ribosome);GO:0042026(protein refolding);GO:0044183(protein binding involved in protein folding);GO:0048046(apoplast);GO:0051082(unfolded protein binding);GO:0051085(chaperone cofactor-dependent protein refolding)	03018(RNA degradation)	*AT1G55490*	chaperonin 60 beta
GH_A05	GO:0003677(DNA binding);GO:0003700(DNA binding	NA	*AT1G52150*	Homeobox-leucine zipper family

G1328	transcription factor activity);GO:0005634(nucleus);GO:0006351(transcription, DNA-templated);GO:0006355(regulation of transcription, DNA-templated);GO:0008289(lipid binding);GO:0009855(determination of bilateral symmetry);GO:0009965(leaf morphogenesis);GO:0010014(meristem initiation);GO:0010073(meristem maintenance);GO:0010075(regulation of meristem growth);GO:0010087(phloem or xylem histogenesis);GO:0030154(cell differentiation);GO:0048263(determination of dorsal identity);GO:0080060(integument development);GO:1905392(plant organ morphogenesis)			protein / lipid-binding START domain-containing protein
GH_A05 G1329	GO:0003674(molecular_function);GO:0005634(nucleus);GO:0008150(biological_process)	NA	*AT5G25840*	DUF1677 family protein (DUF1677)
GH_A05 G1330	GO:0003674(molecular_function);GO:0005575(cellular_component);GO:0008150(biological_process)	04120(Ubiquitin mediated proteolysis)	*AT1G52140*	Avr9/Cf-9 rapidly elicited protein
GH_A05 G1332	GO:0005886(plasma membrane);GO:0016021(integral component of membrane);GO:0042626(ATPase activity, coupled to transmembrane movement of substances)	02010(ABC transporters)	*AT1G59870*	ABC-2 and Plant PDR ABC-type transporter family protein
GH_A05 G1333	GO:0009723(response to ethylene)	NA	*AT3G16350*	Homeodomain-like superfamily protein
GH_A05 G1335	GO:0000160(phosphorelay signal transduction system);GO:0005634(nucleus);GO:0005737(cytoplasm);GO:0009736(cytokinin-activated signaling pathway);GO:0009927(histidine	04075(Plant hormone signal transduction)	*AT3G16360*	HPT phosphotransmitter 4

	phosphotransfer kinase activity);GO:0016310(phosphorylation);GO:0043424(protein histidine kinase binding)			
GH_A05 G1337	GO:0003674(molecular_function);GO:0005634(nucleus);GO:0006351(transcription, DNA-templated);GO:0045892(negative regulation of transcription, DNA-templated)	NA	*AT2G30395*	ovate family protein 17
GH_A05 G1338	GO:0001678(cellular glucose homeostasis);GO:0004340(glucokinase activity);GO:0005829(cytosol);GO:0006096(glycolytic process);GO:0008865(fructokinase activity);GO:0019158(mannokinase activity)	00010(Glycolysis / Gluconeogenesis);00051(Fructose and mannose metabolism);00052(Galactose metabolism);00500(Starch and sucrose metabolism);00520(Amino sugar and nucleotide sugar metabolism);00524(Neomycin, kanamycin and gentamicin biosynthesis)	*AT2G19860*	hexokinase 2
GH_A05 G1339	GO:0005516(calmodulin binding);GO:0005794(Golgi apparatus);GO:0008150(biological_process)	NA	*AT3G16490*	IQ-domain 26
GH_A05 G1346	GO:0003674(molecular_function);GO:0005634(nucleus);GO:0008150(biological_process)	04712(Circadian rhythm - plant)	*AT3G21320*	EARLY FLOWERING protein
GH_A05 G1350	NA	NA	*AT3G21305*	hypothetical protein
GH_A05 G1352	GO:0003674(molecular_function);GO:0005634(nucleus);GO:0008150(biological_process)	00230(Purine metabolism)	*AT1G51745*	Tudor/PWWP/MBT superfamily protein
GH_A05	GO:0005737(cytoplasm);GO:0008150(biological_process)	NA	*AT1G51730*	Ubiquitin-conjugating enzyme

G1353				family protein
GH_A05 G1354	NA	NA	-	-
GH_A05 G1369	GO:0004565(beta-galactosidase activity);GO:0005773(vacuole)	00052(Galactose metabolism);00600(Sphingolipid metabolism);00531(Glycosamino glycan degradation);00604(Glycosphing olipid biosynthesis - ganglio series);00511(Other glycan degradation)	*AT1G72990*	beta-galactosidase 17
GH_A05 G1371	GO:0005515(protein binding);GO:0005634(nucleus);GO:0006351(transcription, DNA-templated);GO:0006355(regulation of transcription, DNA-templated);GO:0016021(integral component of membrane);GO:0016592(mediator complex)	NA	*AT1G29580*	mediator of RNA polymerase II transcription subunit
GH_A05 G1373	GO:0003674(molecular_function);GO:0005886(plasma membrane);GO:0008150(biological_process);GO:0009506(plasmo desma);GO:0016021(integral component of membrane)	NA	*AT1G17620*	Late embryogenesis abundant (LEA) hydroxyproline-rich glycoprotein family
GH_A05 G1375	GO:0005576(extracellular region);GO:0007267(cell-cell signaling);GO:0009553(embryo sac development);GO:0010430(fatty acid omega-oxidation);GO:0016614(oxidoreductase activity, acting on CH-OH group of donors);GO:0016832(aldehyde-lyase activity);GO:0046593(mandelonitrile lyase activity);GO:0050660(flavin adenine dinucleotide	00073(Cutin, suberine and wax biosynthesis)	*AT1G72970*	Glucose-methanol-choline (GMC) oxidoreductase family protein

	binding);GO:0055114(oxidation-reduction process)			
GH_A05 G1377	GO:0000981(RNA polymerase II transcription factor activity, sequence-specific DNA binding);GO:0001135(transcription factor activity, RNA polymerase II transcription factor recruiting);GO:0003677(DNA binding);GO:0003700(DNA binding transcription factor activity);GO:0005634(nucleus);GO:0006351(transcription, DNA-templated);GO:0006355(regulation of transcription, DNA-templated);GO:0006357(regulation of transcription by RNA polymerase II);GO:0009739(response to gibberellin);GO:0009834(plant-type secondary cell wall biogenesis);GO:0009901(anther dehiscence);GO:0030154(cell differentiation);GO:0043565(sequence-specific DNA binding);GO:0044212(transcription regulatory region DNA binding)	NA	*AT3G13890*	myb domain protein 26
GH_A05 G1378	GO:0005516(calmodulin binding);GO:0005737(cytoplasm);GO:0008150(biological_process)	03015(mRNA surveillance pathway)	*AT1G72670*	IQ-domain 8
GH_A05 G1379	GO:0010052(guard cell differentiation)	NA	*AT3G13898*	EPIDERMAL PATTERNING FACTOR-like protein
GH_A05 G1380	GO:0004012(phospholipid-translocating ATPase activity);GO:0005802(trans-Golgi network);GO:0005886(plasma membrane);GO:0048194(Golgi vesicle budding)	NA	*AT1G17500*	ATPase E1-E2 type family protein / haloacid dehalogenase-like hydrolase family protein
GH_A05 G1381	GO:0004674(protein serine/threonine kinase activity);GO:0005634(nucleus);GO:0005737(cytoplasm);GO:0006897(endocytosis);GO:0008360(regulation of cell	NA	*AT1G72710*	casein kinase 1-like protein 2

	shape);GO:0016055(Wnt signaling pathway);GO:0018105(peptidyl-serine phosphorylation)			
GH_A05 G1382	GO:0003674(molecular_function);GO:0005634(nucleus);GO:0008150(biological_process)	NA	*AT5G11970*	ABC family ABC transporter%2C putative (DUF3511)
GH_A05 G1383	GO:0004004(ATP-dependent RNA helicase activity);GO:0006413(translational initiation);GO:0010468(regulation of gene expression);GO:0010501(RNA secondary structure unwinding)	03013(RNA transport)	*AT3G13920*	eukaryotic translation initiation factor 4A1
GH_A05 G1385	GO:0004742(dihydrolipoyllysine-residue acetyltransferase activity);GO:0005507(copper ion binding);GO:0005739(mitochondrion);GO:0005759(mitochondrial matrix);GO:0006096(glycolytic process);GO:0009507(chloroplast);GO:0009941(chloroplast envelope);GO:0031405(lipoic acid binding);GO:0045254(pyruvate dehydrogenase complex)	00010(Glycolysis / Gluconeogenesis);00020(Citrate cycle (TCA cycle));00620(Pyruvate metabolism)	*AT3G13930*	Dihydrolipoamide acetyltransferase%2C long form protein
GH_A05 G1386	GO:0005739(mitochondrion);GO:0005743(mitochondrial inner membrane);GO:0005744(mitochondrial inner membrane presequence translocase complex);GO:0015031(protein transport);GO:0015450(P-P-bond-hydrolysis-driven protein transmembrane transporter activity);GO:0016020(membrane);GO:0030150(protein import into mitochondrial matrix);GO:0031305(integral component of mitochondrial inner membrane)	NA	*AT1G17530*	translocase of inner mitochondrial membrane 23
GH_A05 G1387	GO:0004722(protein serine/threonine phosphatase activity);GO:0005515(protein binding);GO:0005634(nucleus);GO:0005737(cytoplasm);GO:0006	04016(MAPK signaling pathway - plant);04075(Plant hormone signal transduction)	*AT1G72770*	HYPERSENSITIVE TO ABA1

	470(protein dephosphorylation);GO:0009738(abscisic acid-activated signaling pathway);GO:0046872(metal ion binding)			
GH_A05G1389	GO:0003735(structural constituent of ribosome);GO:0006412(translation)	NA	*AT1G72820*	Mitochondrial substrate carrier family protein
GH_A05G1393	GO:0003674(molecular_function);GO:0005634(nucleus);GO:0009734(auxin-activated signaling pathway)	NA	*AT3G13980*	SKI/DACH domain protein
GH_A05G1394	GO:0004439(phosphatidylinositol-4,5-bisphosphate 5-phosphatase activity);GO:0005634(nucleus);GO:0005774(vacuolar membrane);GO:0007033(vacuole organization);GO:0036092(phosphatidylinositol-3-phosphate biosynthetic process);GO:0042578(phosphoric ester hydrolase activity)	00562(Inositol phosphate metabolism);04070(Phosphatidylinositol signaling system)	*AT3G14205*	Phosphoinositide phosphatase family protein
GH_A05G1395	GO:0003674(molecular_function);GO:0005737(cytoplasm);GO:0009408(response to heat);GO:0009644(response to high light intensity);GO:0042542(response to hydrogen peroxide)	04141(Protein processing in endoplasmic reticulum)	*AT1G54050*	HSP20-like chaperones superfamily protein
GH_A05G1396	GO:0003674(molecular_function);GO:0005575(cellular_component);GO:0009733(response to auxin)	04075(Plant hormone signal transduction)	*AT1G17345*	SAUR-like auxin-responsive protein family
GH_A05G1397	GO:0003674(molecular_function);GO:0005739(mitochondrion);GO:0008150(biological_process)	NA	*AT1G17350*	NADH:ubiquinone oxidoreductase intermediate-associated protein 30
GH_A05G1398	GO:0006457(protein folding)	NA	*AT3G14200*	Chaperone DnaJ-domain superfamily protein
GH_A05G1399	GO:0003674(molecular_function);GO:0005634(nucleus);GO:0008150(biological_process)	NA	*AT1G54070*	Dormancy/auxin associated family protein
GH_A05G1400	GO:0003700(DNA binding transcription factor activity);GO:0005515(protein	NA	*AT3G14180*	sequence-specific DNA binding transcription factor

	binding);GO:0005634(nucleus);GO:0006351(transcription, DNA-templated);GO:0006355(regulation of transcription, DNA-templated);GO:0009793(embryo development ending in seed dormancy);GO:0010431(seed maturation);GO:0043565(sequence-specific DNA binding)			
GH_A05G1401	GO:0003674(molecular_function);GO:0005634(nucleus);GO:0008150(biological_process)	NA	*AT3G14172*	GPI-anchored adhesin-like protein
GH_A05G1402	GO:0000149(SNARE binding);GO:0005484(SNAP receptor activity);GO:0006887(exocytosis);GO:0006906(vesicle fusion);GO:0031201(SNARE complex)	04130(SNARE interactions in vesicular transport);04145(Phagosome)	*AT1G11890*	Synaptobrevin family protein
GH_A05G1403	GO:0005634(nucleus);GO:0008150(biological_process)	NA	*AT1G72390*	nuclear receptor coactivator
GH_A05G1405	GO:0003714(transcription corepressor activity);GO:0005634(nucleus);GO:0009611(response to wounding);GO:0031347(regulation of defense response);GO:1903507(negative regulation of nucleic acid-templated transcription);GO:2000022(regulation of jasmonic acid mediated signaling pathway)	04075(Plant hormone signal transduction)	*AT1G72450*	jasmonate-zim-domain protein 6
GH_A05G1406	GO:0004674(protein serine/threonine kinase activity);GO:0005515(protein binding);GO:0005524(ATP binding);GO:0005576(extracellular region);GO:0006468(protein phosphorylation);GO:0007169(transmembrane receptor protein tyrosine kinase signaling pathway);GO:0009506(plasmodesma);GO:0016021(integral component of membrane);GO:0016301(kinase activity);GO:0080092(regulation of pollen tube growth)	NA	*AT3G42880*	Leucine-rich repeat protein kinase family protein

GH_A05G1407	GO:0000145(exocyst);GO:0005634(nucleus);GO:0005829(cytosol);GO:0006887(exocytosis);GO:0006904(vesicle docking involved in exocytosis)	NA	*AT1G72470*	exocyst subunit exo70 family protein D1
GH_A05G1409	GO:0000290(deadenylation-dependent decapping of nuclear-transcribed mRNA);GO:0000339(RNA cap binding);GO:0000932(P-body);GO:0005634(nucleus)	03018(RNA degradation)	*AT3G14080*	Small nuclear ribonucleoprotein family protein
GH_A05G1411	GO:0006812(cation transport);GO:0006813(potassium ion transport);GO:0006814(sodium ion transport);GO:0015297(antiporter activity);GO:0015491(cation:cation antiporter activity);GO:0016021(integral component of membrane);GO:0055085(transmembrane transport)	NA	*AT1G54115*	cation calcium exchanger 4
GH_A05G1412	GO:0004252(serine-type endopeptidase activity);GO:0005618(cell wall)	NA	*AT3G14067*	Subtilase family protein
GH_A05G1413	GO:0003674(molecular_function);GO:0005739(mitochondrion);GO:0008150(biological_process)	NA	*AT1G54120*	hypothetical protein
GH_A05G1414	GO:0005524(ATP binding);GO:0005886(plasma membrane);GO:0016301(kinase activity);GO:0016310(phosphorylation)	NA	*AT1G72540*	Protein kinase superfamily protein
GH_A05G1416	NA	NA	-	-
GH_A05G1417	GO:0000981(RNA polymerase II transcription factor activity, sequence-specific DNA binding);GO:0001135(transcription factor activity, RNA polymerase II transcription factor recruiting);GO:0003677(DNA binding);GO:0003700(DNA binding transcription factor activity);GO:0005515(protein	NA	*AT5G35550*	Duplicated homeodomain-like superfamily protein

	binding);GO:0005524(ATP binding);GO:0005634(nucleus);GO:0006351(transcription, DNA-templated);GO:0006355(regulation of transcription, DNA-templated);GO:0006357(regulation of transcription by RNA polymerase II);GO:0006633(fatty acid biosynthetic process);GO:0006970(response to osmotic stress);GO:0009813(flavonoid biosynthetic process);GO:0010023(proanthocyanidin biosynthetic process);GO:0030154(cell differentiation);GO:0043565(sequence-specific DNA binding);GO:0044212(transcription regulatory region DNA binding)			
GH_A05G1418	GO:0003674(molecular_function);GO:0005634(nucleus);GO:0009648(photoperiodism);GO:0042753(positive regulation of circadian rhythm);GO:0048511(rhythmic process)	NA	*AT1G17455*	ELF4-like 4
GH_A05G1421	GO:0005576(extracellular region);GO:0005615(extracellular space);GO:0009620(response to fungus);GO:0009627(systemic acquired resistance);GO:0009751(response to salicylic acid);GO:0009817(defense response to fungus, incompatible interaction);GO:0009866(induced systemic resistance, ethylene mediated signaling pathway);GO:0009871(jasmonic acid and ethylene-dependent systemic resistance, ethylene mediated signaling pathway);GO:0016042(lipid catabolic process);GO:0016298(lipase activity);GO:0042742(defense response to bacterium);GO:0050832(defense response to fungus);GO:0052689(carboxylic ester hydrolase activity)	00901(Indole alkaloid biosynthesis)	*AT5G40990*	GDSL lipase 1

GH_A05 G1422	GO:0005576(extracellular region);GO:0005615(extracellular space);GO:0009620(response to fungus);GO:0009627(systemic acquired resistance);GO:0009751(response to salicylic acid);GO:0009817(defense response to fungus, incompatible interaction);GO:0009866(induced systemic resistance, ethylene mediated signaling pathway);GO:0009871(jasmonic acid and ethylene-dependent systemic resistance, ethylene mediated signaling pathway);GO:0016042(lipid catabolic process);GO:0016298(lipase activity);GO:0042742(defense response to bacterium);GO:0050832(defense response to fungus);GO:0052689(carboxylic ester hydrolase activity)	00901(Indole alkaloid biosynthesis)	*AT5G40990*	GDSL lipase 1
GH_A05 G1423	GO:0005576(extracellular region);GO:0016042(lipid catabolic process);GO:0016298(lipase activity);GO:0052689(carboxylic ester hydrolase activity)	00901(Indole alkaloid biosynthesis)	*AT1G53920*	GDSL-motif lipase 5
GH_A05 G1426	GO:0004252(serine-type endopeptidase activity);GO:0005618(cell wall)	NA	*AT3G14240*	Subtilase family protein
GH_A05 G1427	GO:0005085(guanyl-nucleotide exchange factor activity);GO:0005851(eukaryotic translation initiation factor 2B complex);GO:0006446(regulation of translational initiation);GO:0019509(L-methionine salvage from methylthioadenosine);GO:0046523(S-methyl-5-thioribose-1-phosphate isomerase activity)	03013(RNA transport)	*AT1G53900*	Eukaryotic translation initiation factor 2B (eIF-2B) family protein
GH_A05 G1430	GO:0030599(pectinesterase activity);GO:0046910(pectinesterase inhibitor activity);GO:0071944(cell periphery)	00040(Pentose and glucuronate interconversions)	*AT1G53840*	pectin methylesterase 1
GH_A05 G1431	GO:0030599(pectinesterase activity);GO:0046910(pectinesterase inhibitor activity);GO:0071944(cell periphery)	00040(Pentose and glucuronate interconversions)	*AT3G14310*	pectin methylesterase 3

GH_A05 G1434	GO:0043161(proteasome-mediated ubiquitin-dependent protein catabolic process);GO:0061630(ubiquitin protein ligase activity)	NA	*AT1G04360*	RING/U-box superfamily protein
GH_A05 G1435	GO:0005886(plasma membrane);GO:0008150(biological_process)	00562(Inositol phosphate metabolism);04070(Phosphatidyl inositol signaling system);04933(AGE-RAGE signaling pathway in diabetic complications)	*AT1G53800*	muscle M-line assembly protein
GH_D06 G0291	GO:0003723(RNA binding);GO:0004519(endonuclease activity);GO:0008270(zinc ion binding);GO:0009451(RNA modification);GO:0009507(chloroplast);GO:0009793(embryo development ending in seed dormancy);GO:0043231(intracellular membrane-bounded organelle)	NA	*AT2G22070*	pentatricopeptide (PPR) repeat-containing protein
GH_D06 G0292	NA	00630(Glyoxylate and dicarboxylate metabolism);00260(Glycine, serine and threonine metabolism);00460(Cyanoamino acid metabolism);00670(One carbon pool by folate)	-	-
GH_D06 G0293	GO:0003674(molecular_function);GO:0005739(mitochondrion);GO:0008150(biological_process);GO:0009451(RNA modification);GO:0016020(membrane)	NA	*AT1G09900*	Pentatricopeptide repeat (PPR-like) superfamily protein
GH_D06 G0296	GO:0005634(nucleus);GO:0008150(biological_process);GO:0016021(integral component of membrane)	NA	*AT1G09920*	TRAF-type zinc finger-like protein
GH_D06	GO:0004842(ubiquitin-protein transferase	04120(Ubiquitin mediated	*AT1G55860*	LOW protein: E3 ubiquitin

G0298	activity);GO:0005634(nucleus);GO:0005737(cytoplasm)	proteolysis)		ligase-like protein
GH_D06 G0300	GO:0003674(molecular_function);GO:0005634(nucleus);GO:0008150(biological_process)	NA	*AT5G09570*	Cox19-like CHCH family protein
GH_D06 G0301	GO:0000977(RNA polymerase II regulatory region sequence-specific DNA binding);GO:0001085(RNA polymerase II transcription factor binding);GO:0001228(transcriptional activator activity, RNA polymerase II transcription regulatory region sequence-specific DNA binding);GO:0003682(chromatin binding);GO:0005634(nucleus);GO:0005667(transcription factor complex);GO:0030154(cell differentiation)	NA	*AT3G21175*	ZIM-like 1
GH_D06 G0303	GO:0009813(flavonoid biosynthetic process);GO:0043231(intracellular membrane-bounded organelle);GO:0052696(flavonoid glucuronidation);GO:0080043(quercetin 3-O-glucosyltransferase activity);GO:0080044(quercetin 7-O-glucosyltransferase activity)	NA	*AT5G49690*	UDP-Glycosyltransferase superfamily protein
GH_D06 G0305	NA	NA	*AT1G58225*	hypothetical protein
GH_D06 G0309	GO:0000778(condensed nuclear chromosome kinetochore);GO:0005886(plasma membrane);GO:0009793(embryo development ending in seed dormancy)	NA	*AT1G58215*	spindle pole body component 110
GH_D06 G0311	GO:0003777(microtubule motor activity);GO:0005737(cytoplasm);GO:0007018(microtubule-based movement)	NA	*AT5G33300*	chromosome-associated kinesin-like protein
GH_D06 G0313	GO:0005216(ion channel activity);GO:0005886(plasma membrane);GO:0006811(ion	NA	*AT1G58200*	MSCS-like 3

	transport);GO:0009507(chloroplast);GO:0009526(plastid envelope);GO:0009657(plastid organization);GO:0010020(chloroplast fission);GO:0016020(membrane);GO:0016021(integral component of membrane);GO:0031969(chloroplast membrane);GO:0055085(transmembrane transport)			
GH_D06G0318	GO:0004843(thiol-dependent ubiquitin-specific protease activity);GO:0005515(protein binding);GO:0005576(extracellular region);GO:0006511(ubiquitin-dependent protein catabolic process);GO:0008283(cell proliferation);GO:0009651(response to salt stress);GO:0009908(flower development);GO:0016021(integral component of membrane);GO:0016579(protein deubiquitination);GO:0036459(thiol-dependent ubiquitinyl hydrolase activity);GO:0046872(metal ion binding);GO:0048364(root development);GO:0048366(leaf development);GO:0048367(shoot system development);GO:1901000(regulation of response to salt stress)	NA	*AT4G24560*	ubiquitin-specific protease 16
GH_D06G0319	GO:0005737(cytoplasm)	NA	*AT1G09740*	Adenine nucleotide alpha hydrolases-like superfamily protein
GH_D06G0320	GO:0004347(glucose-6-phosphate isomerase activity);GO:0005829(cytosol);GO:0005982(starch metabolic process);GO:0006094(gluconeogenesis);GO:0006096(glycolytic process);GO:0009507(chloroplast);GO:0009536(plastid);GO:0009570(chloroplast stroma);GO:0009911(positive regulation of flower	00010(Glycolysis / Gluconeogenesis);00030(Pentose phosphate pathway);00500(Starch and sucrose	*AT4G24620*	phosphoglucose isomerase 1

	development);GO:0009941(chloroplast envelope)	metabolism);00520(Amino sugar and nucleotide sugar metabolism)		
GH_D06G0321	GO:0005507(copper ion binding)	NA	*AT2G33740*	Nitrogen regulatory PII-like%2C alpha/beta
GH_D06G0322	GO:0004693(cyclin-dependent protein serine/threonine kinase activity);GO:0005515(protein binding);GO:0005634(nucleus);GO:0005737(cytoplasm);GO:0007049(cell cycle);GO:0010440(stomatal lineage progression);GO:0051301(cell division);GO:0051726(regulation of cell cycle)	NA	*AT5G65420*	CYCLIN D4%3B1
GH_D06G0324	GO:0000166(nucleotide binding);GO:0005515(protein binding);GO:0005634(nucleus);GO:0005737(cytoplasm);GO:0005829(cytosol);GO:0005886(plasma membrane);GO:0009506(plasmodesma);GO:0045717(negative regulation of fatty acid biosynthetic process);GO:0080008(Cul4-RING E3 ubiquitin ligase complex)	NA	*AT5G10940*	transducin family protein / WD-40 repeat family protein
GH_D06G0327	GO:0003677(DNA binding);GO:0003700(DNA binding transcription factor activity);GO:0005515(protein binding);GO:0005634(nucleus);GO:0006351(transcription, DNA-templated);GO:0006355(regulation of transcription, DNA-templated);GO:0009793(embryo development ending in seed dormancy);GO:0042803(protein homodimerization activity);GO:0046872(metal ion binding)	NA	*AT4G24660*	homeobox protein 22
GH_D06G0328	GO:0000822(inositol hexakisphosphate binding);GO:0009734(auxin-activated signaling pathway)	04075(Plant hormone signal transduction)	*AT5G49980*	auxin F-box protein 5
GH_D06	GO:0000139(Golgi	04130(SNARE interactions in	*AT4G14600*	Target SNARE coiled-coil domain

G0329	membrane);GO:0003674(molecular_function);GO:0005789(endoplasmic reticulum membrane);GO:0005794(Golgi apparatus);GO:0005886(plasma membrane);GO:0015031(protein transport);GO:0016021(integral component of membrane);GO:0016192(vesicle-mediated transport)	vesicular transport)		protein
GH_D06 G0330	GO:0004683(calmodulin-dependent protein kinase activity);GO:0005516(calmodulin binding);GO:0005634(nucleus);GO:0005737(cytoplasm);GO:0005886(plasma membrane);GO:0009738(abscisic acid-activated signaling pathway);GO:0009931(calcium-dependent protein serine/threonine kinase activity);GO:0018105(peptidyl-serine phosphorylation);GO:0035556(intracellular signal transduction);GO:0046777(protein autophosphorylation)	04626(Plant-pathogen interaction)	*AT1G12680*	phosphoenolpyruvate carboxylase-related kinase 2
GH_D06 G0332	NA	NA	-	-
GH_D06 G0334	GO:0006855(drug transmembrane transport)	NA	*AT5G65380*	MATE efflux family protein
GH_D06 G0335	GO:0005543(phospholipid binding);GO:0005739(mitochondrion);GO:0005794(Golgi apparatus);GO:0005905(clathrin-coated pit);GO:0006897(endocytosis);GO:0030136(clathrin-coated vesicle)	NA	*AT4G40080*	ENTH/ANTH/VHS superfamily protein
GH_D06 G0336	GO:0003674(molecular_function);GO:0005737(cytoplasm);GO:0005829(cytosol);GO:0008150(biological_process)	NA	*AT4G09060*	hypothetical protein
GH_D06 G0337	GO:0046658(anchored component of plasma membrane)	NA	*AT1G72230*	Cupredoxin superfamily protein

GH_D06 G0338	GO:0046658(anchored component of plasma membrane)	NA	*AT1G72230*	Cupredoxin superfamily protein
GH_D06 G0341	GO:0003735(structural constituent of ribosome);GO:0005762(mitochondrial large ribosomal subunit);GO:0006412(translation)	NA	*AT5G40080*	Mitochondrial ribosomal protein L27
GH_D06 G0342	GO:0003674(molecular_function);GO:0005575(cellular_component);GO:0008150(biological_process)	NA	*AT1G72240*	hypothetical protein
GH_D06 G0345	GO:0005515(protein binding);GO:0005634(nucleus);GO:0006883(cellular sodium ion homeostasis);GO:0030007(cellular potassium ion homeostasis);GO:0043433(negative regulation of DNA binding transcription factor activity);GO:1901001(negative regulation of response to salt stress)	NA	*AT1G78310*	VQ motif-containing protein
GH_D06 G0346	GO:0004022(alcohol dehydrogenase (NAD) activity);GO:0005737(cytoplasm);GO:0008270(zinc ion binding);GO:0016491(oxidoreductase activity);GO:0055114(oxidation-reduction process)	00010(Glycolysis / Gluconeogenesis);00071(Fatty acid degradation);00350(Tyrosine metabolism)	*AT1G22430*	GroES-like zinc-binding dehydrogenase family protein
GH_D06 G0347	GO:0004842(ubiquitin-protein transferase activity);GO:0005515(protein binding);GO:0005634(nucleus);GO:0006511(ubiquitin-dependent protein catabolic process);GO:0009737(response to abscisic acid);GO:0010608(posttranscriptional regulation of gene expression);GO:0010629(negative regulation of gene expression);GO:0016567(protein ubiquitination)	NA	*AT4G08980*	F-BOX WITH WD-40 2
GH_D06	GO:0004842(ubiquitin-protein transferase	NA	*AT4G08980*	F-BOX WITH WD-40 2

G0348	activity);GO:0005515(protein binding);GO:0005634(nucleus);GO:0006511(ubiquitin-dependent protein catabolic process);GO:0009737(response to abscisic acid);GO:0010608(posttranscriptional regulation of gene expression);GO:0010629(negative regulation of gene expression);GO:0016567(protein ubiquitination)			
GH_D06 G0349	GO:0004842(ubiquitin-protein transferase activity);GO:0005515(protein binding);GO:0005634(nucleus);GO:0006511(ubiquitin-dependent protein catabolic process);GO:0009737(response to abscisic acid);GO:0010608(posttranscriptional regulation of gene expression);GO:0010629(negative regulation of gene expression);GO:0016567(protein ubiquitination)	NA	*AT4G08980*	F-BOX WITH WD-40 2
GH_D06 G0350	GO:0004842(ubiquitin-protein transferase activity);GO:0005515(protein binding);GO:0005634(nucleus);GO:0006511(ubiquitin-dependent protein catabolic process);GO:0009737(response to abscisic acid);GO:0010608(posttranscriptional regulation of gene expression);GO:0010629(negative regulation of gene expression);GO:0016567(protein ubiquitination)	NA	*AT4G08980*	F-BOX WITH WD-40 2
GH_D06 G0352	GO:0003723(RNA binding);GO:0003729(mRNA binding);GO:0008150(biological_process);GO:0016021(integral component of membrane)	03040(Spliceosome)	*AT1G22330*	RNA-binding (RRM/RBD/RNP motifs) family protein
GH_D06 G0353	GO:0005768(endosome);GO:0005774(vacuolar membrane);GO:0005802(trans-Golgi network);GO:0008757(S-adenosylmethionine-dependent	NA	*AT1G78240*	S-adenosyl-L-methionine-dependent methyltransferases superfamily protein

		methyltransferase activity);GO:0032259(methylation)			
	GH_D06G0354	GO:0003756(protein disulfide isomerase activity);GO:0005783(endoplasmic reticulum);GO:0006457(protein folding);GO:0034976(response to endoplasmic reticulum stress)	NA	*AT1G34780*	APR-like 4
	GH_D06G0356	GO:0004722(protein serine/threonine phosphatase activity);GO:0005634(nucleus);GO:0005886(plasma membrane);GO:0006470(protein dephosphorylation);GO:0046872(metal ion binding)	NA	*AT1G34750*	Protein phosphatase 2C family protein
TM3769/TM69636	*GH_A02G0065*	GO:0005515(protein binding);GO:0005777(peroxisome);GO:0005829(cytosol);GO:0009082(branched-chain amino acid biosynthetic process);GO:0009097(isoleucine biosynthetic process);GO:0009099(valine biosynthetic process);GO:0009507(chloroplast);GO:0043621(protein self-association);GO:0050790(regulation of catalytic activity)	00650(Butanoate metabolism);00660(C5-Branched dibasic acid metabolism);00290(Valine, leucine and isoleucine biosynthesis);00770(Pantothenate and CoA biosynthesis)	*AT2G31810*	ACT domain-containing small subunit of acetolactate synthase protein
	GH_A02G0068	GO:0009813(flavonoid biosynthetic process);GO:0043231(intracellular membrane-bounded organelle);GO:0052696(flavonoid glucuronidation);GO:0080043(quercetin 3-O-glucosyltransferase activity);GO:0080044(quercetin 7-O-glucosyltransferase activity)	NA	*AT2G43820*	UDP-glucosyltransferase 74F2
	GH_A02G0069	GO:0003674(molecular_function);GO:0005634(nucleus);GO:0008150(biological_process)	NA	*AT2G44200*	pre-mRNA splicing factor domain-containing protein
	GH_A02G0070	GO:0003712(transcription cofactor activity);GO:0004402(histone acetyltransferase activity);GO:0005516(calmodulin binding);GO:0005634(nucleus);GO:0005737(cytoplasm);GO:0006	03010(Ribosome)	*AT1G05690*	BTB and TAZ domain protein 3

	355(regulation of transcription, DNA-templated);GO:0008270(zinc ion binding);GO:0009409(response to cold);GO:0009553(embryo sac development);GO:0009555(pollen development);GO:0009651(response to salt stress);GO:0009723(response to ethylene);GO:0009751(response to salicylic acid);GO:0019005(SCF ubiquitin ligase complex);GO:0030162(regulation of proteolysis);GO:0031625(ubiquitin protein ligase binding);GO:0042542(response to hydrogen peroxide);GO:0043161(proteasome-mediated ubiquitin-dependent protein catabolic process)			
GH_A02G0073	GO:0003779(actin binding);GO:0005515(protein binding);GO:0005618(cell wall);GO:0005634(nucleus);GO:0005886(plasma membrane);GO:0016020(membrane);GO:0016021(integral component of membrane);GO:0030036(actin cytoskeleton organization);GO:0045010(actin nucleation);GO:0051015(actin filament binding);GO:0051016(barbed-end actin filament capping)	NA	*AT2G43800*	Actin-binding FH2 (formin homology 2) family protein
GH_A02G0076	GO:0005515(protein binding);GO:0005524(ATP binding);GO:0005622(intracellular);GO:0005623(cell);GO:0005634(nucleus);GO:0005739(mitochondrion);GO:0005829(cytosol);GO:0006457(protein folding);GO:0009408(response to heat);GO:0009409(response to cold);GO:0009507(chloroplast);GO:0009532(plastid stroma);GO:0009570(chloroplast stroma);GO:0009579(thylakoid);GO:0009941(chloroplast	03040(Spliceosome);04141(Protein processing in endoplasmic reticulum);04144(Endocytosis)	*AT5G49910*	chloroplast heat shock protein 70-2

	envelope);GO:0016032(viral process);GO:0042026(protein refolding);GO:0045036(protein targeting to chloroplast);GO:0046686(response to cadmium ion);GO:0051082(unfolded protein binding)			
GH_A02G0082	GO:0003674(molecular_function);GO:0005575(cellular_component);GO:0008150(biological_process)	NA	*AT3G59210*	F-box/RNI-like superfamily protein
GH_A02G0097	NA	NA	-	-
GH_A02G0100	GO:0005516(calmodulin binding);GO:0005634(nucleus);GO:0005886(plasma membrane)	NA	*AT3G59690*	IQ-domain 13
GH_A02G0101	GO:0003674(molecular_function);GO:0005634(nucleus);GO:0008380(RNA splicing)	00970(Aminoacyl-tRNA biosynthesis)	*AT1G17130*	coiled-coil protein (DUF572)
GH_A02G0103	GO:0001046(core promoter sequence-specific DNA binding);GO:0001228(transcriptional activator activity, RNA polymerase II transcription regulatory region sequence-specific DNA binding);GO:0005634(nucleus);GO:0006366(transcription by RNA polymerase II)	NA	*AT1G05710*	basic helix-loop-helix (bHLH) DNA-binding superfamily protein
GH_A02G0105	GO:0005524(ATP binding);GO:0005634(nucleus);GO:0006952(defense response);GO:0043531(ADP binding)	NA	*AT3G14460*	LRR and NB-ARC domains-containing disease resistance protein
GH_A02G0108	GO:0005737(cytoplasm);GO:0005829(cytosol);GO:0006007(glucose catabolic process);GO:0006096(glycolytic process);GO:0009506(plasmodesma);GO:0009555(pollen development);GO:0010118(stomatal movement);GO:0030145(manganese ion binding);GO:0046537(2,3-bisphosphoglycerate-independent	00010(Glycolysis / Gluconeogenesis);00260(Glycine, serine and threonine metabolism)	*AT3G08590*	Phosphoglycerate mutase%2C 2%2C3-bisphosphoglycerate-independent

	phosphoglycerate mutase activity);GO:0046686(response to cadmium ion);GO:0048046(apoplast)			
GH_A02G0119	GO:0003700(DNA binding transcription factor activity);GO:0005634(nucleus);GO:0006355(regulation of transcription, DNA-templated);GO:0009723(response to ethylene);GO:0009737(response to abscisic acid);GO:0009863(salicylic acid mediated signaling pathway);GO:0009867(jasmonic acid mediated signaling pathway);GO:0009938(negative regulation of gibberellic acid mediated signaling pathway);GO:0010187(negative regulation of seed germination);GO:0042538(hyperosmotic salinity response);GO:0043565(sequence-specific DNA binding);GO:2000033(regulation of seed dormancy process);GO:2000377(regulation of reactive oxygen species metabolic process)	04075(Plant hormone signal transduction)	*AT1G14920*	GRAS family transcription factor family protein
GH_A02G0120	GO:0003674(molecular_function);GO:0005575(cellular_component);GO:0008150(biological_process)	NA	*AT1G08760*	GPI-anchored adhesin-like protein%2C putative (DUF936)
GH_A02G0121	GO:0005509(calcium ion binding);GO:0005783(endoplasmic reticulum);GO:0005788(endoplasmic reticulum lumen);GO:0005789(endoplasmic reticulum membrane);GO:0006457(protein folding);GO:0009507(chloroplast);GO:0009626(plant-type hypersensitive response);GO:0010204(defense response signaling pathway, resistance gene-independent);GO:0030246(carbohydrate binding);GO:0042742(defense response to bacterium);GO:0046283(anthocyanin-containing compound	04141(Protein processing in endoplasmic reticulum);04145(Phagosome)	*AT1G08450*	calreticulin 3

	metabolic process);GO:0051082(unfolded protein binding);GO:0055074(calcium ion homeostasis)			
GH_A02G0124	GO:0000398(mRNA splicing, via spliceosome);GO:0003676(nucleic acid binding);GO:0005634(nucleus)	NA	*AT2G43410*	RNA binding protein
GH_A02G0125	GO:0005515(protein binding);GO:0005634(nucleus);GO:0016567(protein ubiquitination);GO:0019005(SCF ubiquitin ligase complex)	NA	*AT1G06110*	SKP1/ASK-interacting protein 16
GH_A02G0126	GO:0003700(DNA binding transcription factor activity);GO:0005634(nucleus)	04016(MAPK signaling pathway - plant)	*AT1G06070*	Basic-leucine zipper (bZIP) transcription factor family protein
GH_A02G0128	NA	NA	*AT4G26020*	4/1 protein short form protein
GH_A02G0130	GO:0008420(CTD phosphatase activity);GO:0016591(DNA-directed RNA polymerase II, holoenzyme);GO:0070940(dephosphorylation of RNA polymerase II C-terminal domain)	NA	*AT5G58003*	C-terminal domain phosphatase-like 4
GH_A02G0131	GO:0003674(molecular_function);GO:0005793(endoplasmic reticulum-Golgi intermediate compartment);GO:0008150(biological_process);GO:0016021(integral component of membrane)	NA	*AT1G30890*	Integral membrane HRF1 family protein
GH_A02G0135	GO:0000408(EKC/KEOPS complex);GO:0002949(tRNA threonylcarbamoyladenosine modification)	NA	*AT4G22720*	Actin-like ATPase superfamily protein
GH_A02G0137	GO:0005509(calcium ion binding)	04626(Plant-pathogen interaction)	*AT1G05990*	EF hand calcium-binding protein family
GH_A02G0141	NA	NA	*AT2G44380*	Cysteine/Histidine-rich C1 domain family protein

GH_A02G0143	GO:0004190(aspartic-type endopeptidase activity);GO:0006508(proteolysis);GO:0030163(protein catabolic process)	NA	*AT1G05840*	Eukaryotic aspartyl protease family protein
GH_A02G0144	GO:0000139(Golgi membrane);GO:0003924(GTPase activity);GO:0005515(protein binding);GO:0005525(GTP binding);GO:0005794(Golgi apparatus);GO:0005795(Golgi stack);GO:0005798(Golgi-associated vesicle);GO:0005802(trans-Golgi network);GO:0005829(cytosol);GO:0005886(plasma membrane);GO:0006886(intracellular protein transport);GO:0009408(response to heat);GO:0048219(inter-Golgi cisterna vesicle-mediated transport)	04144(Endocytosis)	*AT1G05810*	Rab GTPase-like A5A protein
GH_A02G0145	GO:0004806(triglyceride lipase activity);GO:0005811(lipid droplet);GO:0006629(lipid metabolic process);GO:0008970(phosphatidylcholine 1-acylhydrolase activity);GO:0009507(chloroplast);GO:0009611(response to wounding);GO:0009695(jasmonic acid biosynthetic process);GO:0016042(lipid catabolic process);GO:0030308(negative regulation of cell growth);GO:0047714(galactolipase activity);GO:0050832(defense response to fungus);GO:0052739(phosphatidylserine 1-acylhydrolase activity);GO:0052740(1-acyl-2-lysophosphatidylserine acylhydrolase activity);GO:0102549(1-18:1-2-16:0-monogalactosyldiacylglycerol lipase activity)	00564(Glycerophospholipid metabolism);00592(alpha-Linolenic acid metabolism)	*AT1G05800*	alpha/beta-Hydrolases superfamily protein

GH_A02 G0148	GO:0005634(nucleus);GO:0005794(Golgi apparatus);GO:0015031(protein transport);GO:0030136(clathrin-coated vesicle)	04144(Endocytosis)	*AT2G43160*	ENTH/VHS family protein
GH_A02 G0157	NA	NA	-	-
GH_A02 G0159	GO:0000151(ubiquitin ligase complex);GO:0000209(protein polyubiquitination);GO:0005737(cytoplasm);GO:0031624(ubiquitin conjugating enzyme binding);GO:0032436(positive regulation of proteasomal ubiquitin-dependent protein catabolic process);GO:0061630(ubiquitin protein ligase activity)	NA	*AT2G31510*	IBR domain-containing protein
GH_A02 G0160	GO:0004683(calmodulin-dependent protein kinase activity);GO:0005516(calmodulin binding);GO:0005634(nucleus);GO:0005737(cytoplasm);GO:0005886(plasma membrane);GO:0009738(abscisic acid-activated signaling pathway);GO:0009931(calcium-dependent protein serine/threonine kinase activity);GO:0018105(peptidyl-serine phosphorylation);GO:0035556(intracellular signal transduction);GO:0046777(protein autophosphorylation)	04626(Plant-pathogen interaction)	*AT2G31500*	calcium-dependent protein kinase 24
GH_A02 G0163	GO:0008168(methyltransferase activity);GO:0009507(chloroplast);GO:0032259(methylation)	NA	*AT2G43320*	S-adenosyl-L-methionine-dependent methyltransferases superfamily protein
GH_A02 G0165	GO:0003677(DNA binding);GO:0003700(DNA binding transcription factor activity);GO:0005634(nucleus);GO:0006351(transcription, DNA-templated);GO:0006355(regulation of transcription, DNA-templated);GO:0007275(multicellular organism	NA	*AT5G39820*	NAC domain containing protein 94

	development);GO:0009733(response to auxin);GO:0045770(positive regulation of asymmetric cell division);GO:0048103(somatic stem cell division);GO:0048829(root cap development)			
GH_D08G2255	GO:0003674(molecular_function);GO:0008150(biological_process);GO:0009507(chloroplast);GO:0009536(plastid)	NA	*AT2G28430*	zinc finger%2C C3HC4 type family protein
GH_D08G2256	GO:0003674(molecular_function);GO:0008150(biological_process);GO:0016021(integral component of membrane);GO:0031225(anchored component of membrane)	NA	*AT2G28410*	transmembrane protein
GH_D08G2257	GO:0005773(vacuole);GO:0005887(integral component of plasma membrane);GO:0009506(plasmodesma);GO:0009992(cellular water homeostasis);GO:0015250(water channel activity);GO:0015254(glycerol channel activity);GO:0034220(ion transmembrane transport)	NA	*AT2G37170*	plasma membrane intrinsic protein 2
GH_D08G2258	GO:0000027(ribosomal large subunit assembly);GO:0003735(structural constituent of ribosome);GO:0006412(translation);GO:0019843(rRNA binding);GO:0022625(cytosolic large ribosomal subunit)	03010(Ribosome)	*AT5G60670*	Ribosomal protein L11 family protein
GH_D08G2260	GO:0003725(double-stranded RNA binding);GO:0005515(protein binding);GO:0005634(nucleus);GO:0005737(cytoplasm);GO:0035196(production of miRNAs involved in gene silencing by miRNA);GO:0070919(production of siRNA involved in chromatin silencing by small RNA)	NA	*AT2G28380*	dsRNA-binding protein 2
GH_D08G2261	GO:0003677(DNA binding);GO:0003700(DNA binding transcription factor activity);GO:0005634(nucleus);GO:0006351(transcription,	NA	*AT5G60690*	Homeobox-leucine zipper family protein / lipid-binding START domain-containing protein

	DNA-templated);GO:0006355(regulation of transcription, DNA-templated);GO:0008289(lipid binding);GO:0009855(determination of bilateral symmetry);GO:0009944(polarity specification of adaxial/abaxial axis);GO:0009956(radial pattern formation);GO:0010014(meristem initiation);GO:0010051(xylem and phloem pattern formation);GO:0010089(xylem development);GO:0030154(cell differentiation);GO:0043565(sequence-specific DNA binding);GO:0044212(transcription regulatory region DNA binding)			
GH_D08G2262	GO:0003674(molecular_function);GO:0005886(plasma membrane);GO:0008150(biological_process);GO:0016021(integral component of membrane)	NA	*AT2G28370*	Uncharacterized protein family (UPF0497)
GH_D08G2264	GO:0000976(transcription regulatory region sequence-specific DNA binding);GO:0000977(RNA polymerase II regulatory region sequence-specific DNA binding);GO:0000987(proximal promoter sequence-specific DNA binding);GO:0003677(DNA binding);GO:0003700(DNA binding transcription factor activity);GO:0005515(protein binding);GO:0005634(nucleus);GO:0006351(transcription, DNA-templated);GO:0043078(polar nucleus);GO:0043565(sequence-specific DNA binding);GO:0045944(positive regulation of transcription by RNA polymerase II);GO:0046983(protein dimerization activity)	NA	*AT5G48670*	AGAMOUS-like 80
GH_D08	GO:0000976(transcription regulatory region sequence-specific	NA	*AT5G48670*	AGAMOUS-like 80

G2265	DNA binding);GO:0000977(RNA polymerase II regulatory region sequence-specific DNA binding);GO:0000987(proximal promoter sequence-specific DNA binding);GO:0003677(DNA binding);GO:0003700(DNA binding transcription factor activity);GO:0005515(protein binding);GO:0005634(nucleus);GO:0006351(transcription, DNA-templated);GO:0043078(polar nucleus);GO:0043565(sequence-specific DNA binding);GO:0045944(positive regulation of transcription by RNA polymerase II);GO:0046983(protein dimerization activity)			
GH_D08 G2266	GO:0000976(transcription regulatory region sequence-specific DNA binding);GO:0000977(RNA polymerase II regulatory region sequence-specific DNA binding);GO:0000987(proximal promoter sequence-specific DNA binding);GO:0003677(DNA binding);GO:0003700(DNA binding transcription factor activity);GO:0005515(protein binding);GO:0005634(nucleus);GO:0006351(transcription, DNA-templated);GO:0043078(polar nucleus);GO:0043565(sequence-specific DNA binding);GO:0045944(positive regulation of transcription by RNA polymerase II);GO:0046983(protein dimerization activity)	NA	*AT5G48670*	AGAMOUS-like 80
GH_D08 G2269	GO:0000398(mRNA splicing, via spliceosome);GO:0005634(nucleus);GO:0005737(cytoplasm);GO:0006464(cellular protein modification process);GO:0031386(protein tag)	NA	*AT5G42300*	ubiquitin-like protein 5
GH_D08	GO:0005464(UDP-xylose transmembrane transporter	NA	*AT2G28315*	Nucleotide/sugar transporter

G2271	activity);GO:0005783(endoplasmic reticulum);GO:0005794(Golgi apparatus);GO:0005886(plasma membrane);GO:0015790(UDP-xylose transmembrane transport);GO:0016020(membrane)			family protein
GH_D08G2272	GO:0009058(biosynthetic process);GO:0016021(integral component of membrane);GO:0016740(transferase activity);GO:0016757(transferase activity, transferring glycosyl groups)	NA	*AT5G60700*	glycosyltransferase family protein 2
GH_D08G2275	GO:0004842(ubiquitin-protein transferase activity);GO:0005576(extracellular region);GO:0005886(plasma membrane);GO:0008270(zinc ion binding)	NA	*AT5G60710*	Zinc finger (C3HC4-type RING finger) family protein
GH_D08G2276	GO:0004386(helicase activity);GO:0005524(ATP binding);GO:0005634(nucleus);GO:0006342(chromatin silencing);GO:0006344(maintenance of chromatin silencing);GO:0006351(transcription, DNA-templated);GO:0031047(gene silencing by RNA);GO:0032259(methylation);GO:0042803(protein homodimerization activity);GO:0046872(metal ion binding);GO:0051537(2 iron, 2 sulfur cluster binding)	NA	*AT1G08060*	ATP-dependent helicase family protein
GH_D08G2277	GO:0005515(protein binding);GO:0005634(nucleus);GO:0005975(carbohydrate metabolic process);GO:0009744(response to sucrose);GO:0019887(protein kinase regulator activity);GO:0043562(cellular response to nitrogen levels)	NA	*AT2G28060*	5'-AMP-activated protein kinase beta-2 subunit protein
GH_D08G2279	NA	NA	-	-

GH_D08G2282	GO:0000981(RNA polymerase II transcription factor activity, sequence-specific DNA binding);GO:0001135(transcription factor activity, RNA polymerase II transcription factor recruiting);GO:0003677(DNA binding);GO:0003700(DNA binding transcription factor activity);GO:0005634(nucleus);GO:0006351(transcription, DNA-templated);GO:0006355(regulation of transcription, DNA-templated);GO:0006357(regulation of transcription by RNA polymerase II);GO:0008285(negative regulation of cell proliferation);GO:0030154(cell differentiation);GO:0035987(endodermal cell differentiation);GO:0043565(sequence-specific DNA binding);GO:0044212(transcription regulatory region DNA binding);GO:0045597(positive regulation of cell differentiation);GO:0045892(negative regulation of transcription, DNA-templated);GO:0045893(positive regulation of transcription, DNA-templated);GO:0048226(Casparian strip);GO:2000067(regulation of root morphogenesis)	NA	*AT5G57620*	myb domain protein 36
GH_D08G2288	GO:0003674(molecular_function);GO:0005634(nucleus);GO:0008150(biological_process)	NA	*AT5G22930*	enabled-like protein (DUF1635)
GH_D08G2291	GO:0004722(protein serine/threonine phosphatase activity);GO:0005634(nucleus);GO:0006470(protein dephosphorylation);GO:0009536(plastid);GO:0009611(response to wounding);GO:0009620(response to fungus);GO:0009738(abscisic acid-activated signaling pathway);GO:0046872(metal ion binding);GO:0050832(defense response to fungus)	NA	*AT2G30020*	Protein phosphatase 2C family protein

GH_D08G2294	GO:0015079(potassium ion transmembrane transporter activity);GO:0016020(membrane)	NA	*AT2G30070*	potassium transporter 1
GH_D08G2295	GO:0005385(zinc ion transmembrane transporter activity);GO:0005886(plasma membrane);GO:0071577(zinc II ion transmembrane transport)	NA	*AT2G30080*	ZIP metal ion transporter family
GH_D08G2296	GO:0004596(peptide alpha-N-acetyltransferase activity);GO:0006474(N-terminal protein amino acid acetylation);GO:0031248(protein acetyltransferase complex)	NA	*AT2G30090*	Acyl-CoA N-acyltransferases (NAT) superfamily protein
GH_D08G2298	GO:0005634(nucleus);GO:0005829(cytosol);GO:0009408(response to heat);GO:0009409(response to cold);GO:0009651(response to salt stress);GO:0010228(vegetative to reproductive phase transition of meristem);GO:0051087(chaperone binding)	04141(Protein processing in endoplasmic reticulum)	*AT3G51780*	BCL-2-associated athanogene 4
GH_D08G2299	NA	NA	*AT2G42380*	Basic-leucine zipper (bZIP) transcription factor family protein
GH_D08G2300	NA	NA	*AT2G42380*	Basic-leucine zipper (bZIP) transcription factor family protein
GH_D08G2301	GO:0003677(DNA binding);GO:0003700(DNA binding transcription factor activity);GO:0005515(protein binding);GO:0005634(nucleus);GO:0006351(transcription, DNA-templated);GO:0006355(regulation of transcription, DNA-templated)	04933(AGE-RAGE signaling pathway in diabetic complications)	*AT2G38300*	myb-like HTH transcriptional regulator family protein
GH_D08G2302	GO:0005515(protein binding);GO:0005634(nucleus);GO:0009693(ethylene biosynthetic process);GO:0009873(ethylene-activated signaling pathway);GO:0010182(sugar mediated signaling pathway);GO:0010364(regulation of ethylene biosynthetic	NA	*AT3G51770*	tetratricopeptide repeat (TPR)-containing protein

	process);GO:0016036(cellular response to phosphate starvation);GO:0016567(protein ubiquitination);GO:0017145(stem cell division);GO:0030674(protein binding, bridging);GO:0043161(proteasome-mediated ubiquitin-dependent protein catabolic process);GO:2000069(regulation of post-embryonic root development)			
GH_D08G2303	GO:0004672(protein kinase activity);GO:0005524(ATP binding);GO:0005886(plasma membrane);GO:0006468(protein phosphorylation);GO:0016021(integral component of membrane);GO:0016301(kinase activity)	NA	*AT4G18640*	Leucine-rich repeat protein kinase family protein
GH_D08G2309	GO:0003824(catalytic activity);GO:0005829(cytosol);GO:0009809(lignin biosynthetic process);GO:0016621(cinnamoyl-CoA reductase activity);GO:0050662(coenzyme binding)	00940(Phenylpropanoid biosynthesis)	*AT5G58490*	NAD(P)-binding Rossmann-fold superfamily protein
GH_D08G2310	GO:0003677(DNA binding);GO:0005515(protein binding);GO:0005634(nucleus);GO:0009965(leaf morphogenesis);GO:0010016(shoot system morphogenesis)	NA	*AT2G30130*	Lateral organ boundaries (LOB) domain family protein
GH_D08G2312	GO:0003674(molecular_function);GO:0005783(endoplasmic reticulum);GO:0010008(endosome membrane);GO:0016021(integral component of membrane);GO:0016192(vesicle-mediated transport)	NA	*AT2G38360*	prenylated RAB acceptor 1.B4
GH_D08G2314	GO:0003674(molecular_function);GO:0008150(biological_process)	NA	*AT2G30230*	6%2C7-dimethyl-8-ribityllumazine synthase
GH_D08G2317	GO:0006952(defense response)	NA	*AT2G30270*	LURP-one-like protein (DUF567)
GH_D08	GO:0005634(nucleus)	NA	*AT2G40435*	transcription factor SCREAM-like

	G2318				protein
	GH_D08 G2319	GO:0004601(peroxidase activity);GO:0005515(protein binding);GO:0005576(extracellular region);GO:0005618(cell wall);GO:0005794(Golgi apparatus);GO:0005829(cytosol);GO:0006979(response to oxidative stress);GO:0009809(lignin biosynthetic process);GO:0010089(xylem development);GO:0020037(heme binding);GO:0042744(hydrogen peroxide catabolic process);GO:0046872(metal ion binding);GO:0048046(apoplast);GO:0055114(oxidation-reduction process);GO:1901430(positive regulation of syringal lignin biosynthetic process)	00940(Phenylpropanoid biosynthesis)	*AT5G05340*	Peroxidase superfamily protein
TM4959/TM6094	*GH_A02 G1218*	NA	NA	-	-
	GH_A02 G1222	GO:0009507(chloroplast);GO:0009535(chloroplast thylakoid membrane);GO:0010258(NADH dehydrogenase complex (plastoquinone) assembly);GO:0010598(NAD(P)H dehydrogenase complex (plastoquinone));GO:0016655(oxidoreductase activity, acting on NAD(P)H, quinone or similar compound as acceptor);GO:0048038(quinone binding);GO:0055114(oxidation-reduction process)	NA	*AT4G37925*	subunit NDH-M of NAD(P)H:plastoquinone dehydrogenase complex
	GH_A02 G1225	GO:0004364(glutathione transferase activity);GO:0005737(cytoplasm);GO:0006749(glutathione metabolic process)	NA	*AT1G77290*	Glutathione S-transferase family protein

GH_A02G1226	NA	NA	*AT2G02650*	Ribonuclease H-like superfamily protein
GH_A02G1229	GO:0003713(transcription coactivator activity);GO:0005622(intracellular);GO:0005634(nucleus);GO:0006974(cellular response to DNA damage stimulus);GO:0007131(reciprocal meiotic recombination);GO:0007140(male meiotic nuclear division);GO:0007143(female meiotic nuclear division);GO:0033314(mitotic DNA replication checkpoint)	03440(Homologous recombination)	*AT1G77320*	transcription coactivator
GH_A02G1231	GO:0009693(ethylene biosynthetic process);GO:0051213(dioxygenase activity)	00270(Cysteine and methionine metabolism)	*AT1G77330*	2-oxoglutarate (2OG) and Fe(II)-dependent oxygenase superfamily protein
GH_A02G1232	GO:0009306(protein secretion);GO:0015450(P-P-bond-hydrolysis-driven protein transmembrane transporter activity)	03060(Protein export)	*AT1G21650*	Preprotein translocase SecA family protein
GH_A02G1233	GO:0003723(RNA binding);GO:0005739(mitochondrion);GO:0008150(biological_process)	NA	*AT1G77360*	Tetratricopeptide repeat (TPR)-like superfamily protein
GH_A03G0412	GO:0005886(plasma membrane);GO:0015112(nitrate transmembrane transporter activity);GO:0015706(nitrate transport)	00910(Nitrogen metabolism)	*AT5G60780*	nitrate transporter 2.3
GH_A03G0413	GO:0005886(plasma membrane);GO:0015112(nitrate transmembrane transporter activity);GO:0015706(nitrate transport)	00910(Nitrogen metabolism)	*AT1G08090*	nitrate transporter 2:1
GH_A03G0416	GO:0005634(nucleus);GO:0010264(myo-inositol hexakisphosphate biosynthetic process);GO:0016787(hydrolase activity)	NA	*AT5G60760*	P-loop containing nucleoside triphosphate hydrolases superfamily protein
GH_A03	GO:0005634(nucleus);GO:0005789(endoplasmic reticulum	00563(Glycosylphosphatidylinos	*AT3G45100*	UDP-Glycosyltransferase

G0417	membrane);GO:0006506(GPI anchor biosynthetic process);GO:0009846(pollen germination);GO:0009860(pollen tube growth);GO:0016021(integral component of membrane);GO:0016757(transferase activity, transferring glycosyl groups);GO:0017176(phosphatidylinositol N-acetylglucosaminyltransferase activity)	itol(GPI)-anchor biosynthesis)		superfamily protein
GH_A03G0418	GO:0005737(cytoplasm);GO:0006508(proteolysis);GO:0008234(cysteine-type peptidase activity)	NA	*AT5G04250*	Cysteine proteinases superfamily protein
GH_A03G0420	GO:0003677(DNA binding);GO:0003700(DNA binding transcription factor activity);GO:0005634(nucleus);GO:0006351(transcription, DNA-templated);GO:0006355(regulation of transcription, DNA-templated);GO:0007389(pattern specification process);GO:0010076(maintenance of floral meristem identity);GO:0010077(maintenance of inflorescence meristem identity);GO:0010223(secondary shoot formation);GO:0010228(vegetative to reproductive phase transition of meristem);GO:0010229(inflorescence development);GO:0080006(internode patterning);GO:1905393(plant organ formation)	NA	*AT2G27990*	BEL1-like homeodomain 8
GH_A03G0422	GO:0004675(transmembrane receptor protein serine/threonine kinase activity);GO:0005524(ATP binding);GO:0005768(endosome);GO:0005886(plasma membrane);GO:0006468(protein phosphorylation);GO:0007166(cell surface receptor signaling pathway);GO:0010008(endosome membrane);GO:0010089(xylem	NA	*AT2G28250*	Protein kinase superfamily protein

	development);GO:0012505(endomembrane system);GO:0016021(integral component of membrane);GO:0016301(kinase activity);GO:0031902(late endosome membrane);GO:0051020(GTPase binding)			
GH_A03G0424	GO:0005634(nucleus);GO:0005829(cytosol);GO:0009691(cytokinin biosynthetic process);GO:0016799(hydrolase activity, hydrolyzing N-glycosyl compounds)	NA	*AT2G37210*	lysine decarboxylase family protein
GH_A03G0426	GO:0009058(biosynthetic process);GO:0016021(integral component of membrane);GO:0016740(transferase activity);GO:0016757(transferase activity, transferring glycosyl groups)	NA	*AT5G60700*	glycosyltransferase family protein 2
GH_A03G0427	GO:0005634(nucleus);GO:0005768(endosome);GO:0005794(Golgi apparatus);GO:0005802(trans-Golgi network);GO:0008150(biological_process);GO:0016021(integral component of membrane);GO:0016757(transferase activity, transferring glycosyl groups)	03013(RNA transport)	*AT2G28310*	trimethylguanosine synthase (DUF707)
GH_A03G0428	GO:0005464(UDP-xylose transmembrane transporter activity);GO:0005783(endoplasmic reticulum);GO:0005794(Golgi apparatus);GO:0005886(plasma membrane);GO:0015790(UDP-xylose transmembrane transport);GO:0016020(membrane)	NA	*AT2G28315*	Nucleotide/sugar transporter family protein
GH_A03G0430	GO:0015015(heparan sulfate proteoglycan biosynthetic process, enzymatic modification);GO:0017095(heparan sulfate 6-O-sulfotransferase activity)	NA	*AT1G08030*	tyrosylprotein sulfotransferase
GH_A03G0431	GO:0000977(RNA polymerase II regulatory region sequence-specific DNA binding);GO:0001085(RNA polymerase II	NA	*AT1G08010*	GATA transcription factor 11

	transcription factor binding);GO:0001228(transcriptional activator activity, RNA polymerase II transcription regulatory region sequence-specific DNA binding);GO:0003682(chromatin binding);GO:0005634(nucleus);GO:0005667(transcription factor complex);GO:0030154(cell differentiation)			
GH_A03G0436	GO:0003677(DNA binding);GO:0003700(DNA binding transcription factor activity);GO:0005634(nucleus);GO:0006351(transcription, DNA-templated);GO:0006355(regulation of transcription, DNA-templated);GO:0008289(lipid binding);GO:0009855(determination of bilateral symmetry);GO:0009944(polarity specification of adaxial/abaxial axis);GO:0009956(radial pattern formation);GO:0010014(meristem initiation);GO:0010051(xylem and phloem pattern formation);GO:0010089(xylem development);GO:0030154(cell differentiation);GO:0043565(sequence-specific DNA binding);GO:0044212(transcription regulatory region DNA binding)	NA	*AT5G60690*	Homeobox-leucine zipper family protein / lipid-binding START domain-containing protein
GH_A03G0440	GO:0012505(endomembrane system);GO:0016192(vesicle-mediated transport)	NA	*AT2G28390*	SAND family protein
GH_A03G0441	GO:0003674(molecular_function);GO:0008150(biological_process)	NA	*AT5G60680*	transcription initiation factor TFIID subunit (Protein of unknown function%2C DUF584)
GH_A03G0442	GO:0000027(ribosomal large subunit assembly);GO:0003735(structural constituent of	03010(Ribosome)	*AT5G60670*	Ribosomal protein L11 family protein

	ribosome);GO:0006412(translation);GO:0019843(rRNA binding);GO:0022625(cytosolic large ribosomal subunit)			
GH_A03 G0444	GO:0005575(cellular_component)	NA	*AT5G42905*	Polynucleotidyl transferase%2C ribonuclease H-like superfamily protein
GH_A03 G0445	GO:0003674(molecular_function);GO:0008150(biological_process);GO:0016021(integral component of membrane);GO:0031225(anchored component of membrane)	NA	*AT2G28410*	transmembrane protein
GH_A03 G0447	GO:0003756(protein disulfide isomerase activity);GO:0005783(endoplasmic reticulum);GO:0006457(protein folding);GO:0034976(response to endoplasmic reticulum stress)	04141(Protein processing in endoplasmic reticulum)	*AT5G60640*	PDI-like 1-4
GH_A03 G0448	GO:0003674(molecular_function);GO:0008150(biological_process);GO:0009505(plant-type cell wall);GO:0016021(integral component of membrane)	NA	*AT3G45230*	hydroxyproline-rich glycoprotein family protein
GH_A03 G0450	GO:0003676(nucleic acid binding);GO:0003677(DNA binding);GO:0005634(nucleus);GO:0006396(RNA processing);GO:0008173(RNA methyltransferase activity);GO:0046872(metal ion binding)	NA	*AT2G28450*	zinc finger (CCCH-type) family protein
GH_A03 G0451	GO:0003723(RNA binding);GO:0003729(mRNA binding);GO:0005515(protein binding);GO:0005634(nucleus);GO:0005737(cytoplasm);GO:0005829(cytosol);GO:0006952(defense response);GO:0008219(cell death);GO:0009693(ethylene biosynthetic process);GO:0009738(abscisic acid-activated signaling pathway);GO:0010150(leaf senescence);GO:0017091(AU-rich	03040(Spliceosome)	*AT2G41060*	RNA-binding (RRM/RBD/RNP motifs) family protein

	element binding);GO:0048255(mRNA stabilization)			
GH_A03 G0460	GO:0004672(protein kinase activity);GO:0004707(MAP kinase activity);GO:0005515(protein binding);GO:0005524(ATP binding);GO:0005634(nucleus);GO:0005737(cytoplasm);GO:0006468(protein phosphorylation);GO:0006970(response to osmotic stress);GO:0006979(response to oxidative stress);GO:0007165(signal transduction);GO:0009409(response to cold);GO:0009555(pollen development);GO:0009611(response to wounding);GO:0009617(response to bacterium);GO:0009626(plant-type hypersensitive response);GO:0009738(abscisic acid-activated signaling pathway);GO:0010120(camalexin biosynthetic process);GO:0010183(pollen tube guidance);GO:0010200(response to chitin);GO:0010224(response to UV-B);GO:0010229(inflorescence development);GO:0010468(regulation of gene expression);GO:0016301(kinase activity);GO:0048481(plant ovule development);GO:0050826(response to freezing);GO:0080136(priming of cellular response to stress);GO:2000037(regulation of stomatal complex patterning);GO:2000038(regulation of stomatal complex development)	04016(MAPK signaling pathway - plant)	*AT3G45640*	mitogen-activated protein kinase 3
GH_A03 G0466	GO:0009507(chloroplast);GO:0016567(protein ubiquitination)	NA	*AT5G60050*	BTB/POZ domain-containing protein
GH_A03 G0468	GO:0005634(nucleus)	NA	*AT5G60030*	hypothetical protein

	GH_A03G0470	GO:0003677(DNA binding);GO:0003700(DNA binding transcription factor activity);GO:0005515(protein binding);GO:0005634(nucleus);GO:0006351(transcription, DNA-templated);GO:0006355(regulation of transcription, DNA-templated);GO:0008283(cell proliferation);GO:0009908(flower development);GO:0009943(adaxial/abaxial axis specification);GO:0009947(centrolateral axis specification);GO:0010865(stipule development);GO:0030154(cell differentiation);GO:0099402(plant organ development)	03013(RNA transport)	*AT2G28610*	Homeodomain-like superfamily protein
TM48233/TM75885	*GH_D01G0691*	GO:0001046(core promoter sequence-specific DNA binding);GO:0001228(transcriptional activator activity, RNA polymerase II transcription regulatory region sequence-specific DNA binding);GO:0005634(nucleus);GO:0006366(transcription by RNA polymerase II)	NA	*AT3G20640*	basic helix-loop-helix (bHLH) DNA-binding superfamily protein
	GH_D01G0692	GO:0009536(plastid)	00190(Oxidative phosphorylation);00195(Photosynthesis)	*ATCG00480*	ATP synthase subunit beta
	GH_D01G0693	GO:0005737(cytoplasm);GO:0009507(chloroplast);GO:0009733(response to auxin);GO:0009734(auxin-activated signaling pathway);GO:0009826(unidimensional cell growth);GO:0010252(auxin homeostasis);GO:0010279(indole-3-acetic acid amido synthetase activity)	04075(Plant hormone signal transduction)	*AT5G54510*	Auxin-responsive GH3 family protein
	GH_D01G0694	GO:0005737(cytoplasm);GO:0009507(chloroplast);GO:0009733(response to auxin);GO:0009734(auxin-activated signaling	04075(Plant hormone signal transduction)	*AT5G54510*	Auxin-responsive GH3 family protein

	pathway);GO:0009826(unidimensional cell growth);GO:0010252(auxin homeostasis);GO:0010279(indole-3-acetic acid amido synthetase activity)			
GH_D01 G0695	GO:0005515(protein binding);GO:0005524(ATP binding);GO:0005634(nucleus);GO:0005886(plasma membrane);GO:0006952(defense response);GO:0007165(signal transduction);GO:0043531(ADP binding);GO:0043621(protein self-association);GO:0050829(defense response to Gram-negative bacterium)	04626(Plant-pathogen interaction)	*AT3G50950*	HOPZ-ACTIVATED RESISTANCE 1
GH_D01 G0696	GO:0008150(biological_process);GO:0009507(chloroplast)	NA	*AT5G54530*	serine protease%2C putative (Protein of unknown function%2C DUF538)
GH_D01 G0697	GO:0004482(mRNA (guanine-N7-)-methyltransferase activity);GO:0005634(nucleus);GO:0005845(mRNA cap binding complex);GO:0006370(7-methylguanosine mRNA capping);GO:0036265(RNA (guanine-N7)-methylation)	03015(mRNA surveillance pathway)	*AT3G20650*	mRNA capping enzyme family protein
GH_D01 G0701	GO:0003700(DNA binding transcription factor activity);GO:0005634(nucleus);GO:0006355(regulation of transcription, DNA-templated);GO:0043565(sequence-specific DNA binding)	04075(Plant hormone signal transduction)	*AT5G48150*	GRAS family transcription factor
GH_D01 G0702	GO:0005515(protein binding)	NA	*AT5G54590*	Protein kinase superfamily protein
GH_D01 G0703	GO:0005739(mitochondrion);GO:0006412(translation)	03010(Ribosome)	*AT5G54600*	Translation protein SH3-like family protein
GH_D01	GO:0000035(acyl binding);GO:0000036(acyl carrier	00190(Oxidative	*AT1G65290*	mitochondrial acyl carrier protein

G0704	activity);GO:0005515(protein binding);GO:0005739(mitochondrion);GO:0005759(mitochondrial matrix);GO:0005829(cytosol);GO:0006633(fatty acid biosynthetic process);GO:0009245(lipid A biosynthetic process);GO:0031177(phosphopantetheine binding);GO:0046872(metal ion binding);GO:0050897(cobalt ion binding);GO:0055114(oxidation-reduction process);GO:0070469(respiratory chain)	phosphorylation)		2
GH_D01 G0705	GO:0000035(acyl binding);GO:0000036(acyl carrier activity);GO:0005515(protein binding);GO:0005739(mitochondrion);GO:0005759(mitochondrial matrix);GO:0005829(cytosol);GO:0006633(fatty acid biosynthetic process);GO:0009245(lipid A biosynthetic process);GO:0031177(phosphopantetheine binding);GO:0046872(metal ion binding);GO:0050897(cobalt ion binding);GO:0055114(oxidation-reduction process);GO:0070469(respiratory chain)	00190(Oxidative phosphorylation)	*AT1G65290*	mitochondrial acyl carrier protein 2
GH_D01 G0706	GO:0003824(catalytic activity);GO:0005634(nucleus);GO:0045551(cinnamyl-alcohol dehydrogenase activity);GO:0050662(coenzyme binding)	00941(Flavonoid biosynthesis)	*AT4G27250*	NAD(P)-binding Rossmann-fold superfamily protein
GH_D01 G0707	GO:0003676(nucleic acid binding);GO:0003700(DNA binding transcription factor activity);GO:0005634(nucleus);GO:0006355(regulation of transcription, DNA-templated)	NA	*AT5G54630*	zinc finger protein-like protein
GH_D01 G0711	GO:0003677(DNA binding);GO:0003700(DNA binding transcription factor activity);GO:0005515(protein	NA	*AT5G54680*	basic helix-loop-helix (bHLH) DNA-binding superfamily protein

	binding);GO:0005634(nucleus);GO:0006351(transcription, DNA-templated);GO:0006355(regulation of transcription, DNA-templated);GO:0019760(glucosinolate metabolic process);GO:0043565(sequence-specific DNA binding);GO:0046983(protein dimerization activity)			
GH_D01 G0713	GO:0000139(Golgi membrane);GO:0005794(Golgi apparatus);GO:0010413(glucuronoxylan metabolic process);GO:0010417(glucuronoxylan biosynthetic process);GO:0016021(integral component of membrane);GO:0016051(carbohydrate biosynthetic process);GO:0016757(transferase activity, transferring glycosyl groups);GO:0016758(transferase activity, transferring hexosyl groups);GO:0045489(pectin biosynthetic process);GO:0045492(xylan biosynthetic process);GO:0047262(polygalacturonate 4-alpha-galacturonosyltransferase activity);GO:0071555(cell wall organization)	NA	*AT5G54690*	galacturonosyltransferase 12
GH_D01 G0717	GO:0003674(molecular_function);GO:0005739(mitochondrion);GO:0008150(biological_process)	NA	*AT4G04790*	Tetratricopeptide repeat (TPR)-like superfamily protein
GH_D11 G1557	GO:0003858(3-hydroxybutyrate dehydrogenase activity);GO:0004616(phosphogluconate dehydrogenase (decarboxylating) activity);GO:0005739(mitochondrion);GO:0005829(cytosol);GO:0006979(response to oxidative stress);GO:0030267(glyoxylate reductase (NADP) activity);GO:0051287(NAD binding);GO:0055114(oxidation-reduction process)	00630(Glyoxylate and dicarboxylate metabolism);00650(Butanoate metabolism)	*AT3G25530*	glyoxylate reductase 1

GH_D11G1558	GO:0003674(molecular_function);GO:0005739(mitochondrion);GO:0009507(chloroplast);GO:0009579(thylakoid)	NA	*AT2G04039*	DUF2996 family protein
GH_D11G1560	GO:0009416(response to light stimulus);GO:0009535(chloroplast thylakoid membrane);GO:0009768(photosynthesis, light harvesting in photosystem I);GO:0009941(chloroplast envelope);GO:0010287(plastoglobule);GO:0016168(chlorophyll binding);GO:0031409(pigment binding)	00196(Photosynthesis - antenna proteins)	*AT1G61520*	PSI type III chlorophyll a/b-binding protein
GH_D11G1561	GO:0016021(integral component of membrane)	NA	*AT4G12650*	Endomembrane protein 70 protein family
GH_D11G1566	GO:0004497(monooxygenase activity);GO:0007275(multicellular organism development);GO:0010268(brassinosteroid homeostasis);GO:0016125(sterol metabolic process);GO:0016132(brassinosteroid biosynthetic process)	00905(Brassinosteroid biosynthesis)	*AT5G14400*	cytochrome P450%2C family 724%2C subfamily A%2C polypeptide 1
GH_D11G1569	GO:0005737(cytoplasm);GO:0030001(metal ion transport);GO:0046914(transition metal ion binding);GO:0046916(cellular transition metal ion homeostasis)	04626(Plant-pathogen interaction)	*AT5G48290*	Heavy metal transport/detoxification superfamily protein
GH_D11G1570	GO:0000139(Golgi membrane);GO:0005794(Golgi apparatus);GO:0006486(protein glycosylation);GO:0016021(integral component of membrane);GO:0016757(transferase activity, transferring glycosyl groups);GO:0071555(cell wall organization)	NA	*AT5G03795*	Exostosin family protein
GH_D11G1571	GO:0003674(molecular_function);GO:0005634(nucleus);GO:0008150(biological_process)	NA	*AT2G04235*	hypothetical protein
GH_D11G1573	GO:0000460(maturation of 5.8S rRNA);GO:0000470(maturation of LSU-rRNA);GO:0030687(preribosome, large subunit precursor)	NA	*AT5G48240*	Rrp15p protein
GH_D11	GO:0005515(protein binding);GO:0006970(response to osmotic	NA	*AT2G04240*	RING/U-box superfamily protein

G1574	stress);GO:0008270(zinc ion binding);GO:0009651(response to salt stress);GO:0009687(abscisic acid metabolic process);GO:0009688(abscisic acid biosynthetic process);GO:0009739(response to gibberellin);GO:0010200(response to chitin);GO:0016021(integral component of membrane);GO:0016567(protein ubiquitination);GO:0043161(proteasome-mediated ubiquitin-dependent protein catabolic process);GO:0046872(metal ion binding);GO:0061630(ubiquitin protein ligase activity)			
GH_D11G1585	GO:0003700(DNA binding transcription factor activity);GO:0005515(protein binding);GO:0005634(nucleus);GO:0006351(transcription, DNA-templated);GO:0006355(regulation of transcription, DNA-templated);GO:0043565(sequence-specific DNA binding);GO:0046982(protein heterodimerization activity)	NA	*AT4G14540*	nuclear factor Y%2C subunit B3
GH_D11G1588	GO:0000139(Golgi membrane);GO:0005794(Golgi apparatus);GO:0005795(Golgi stack);GO:0006486(protein glycosylation);GO:0008375(acetylglucosaminyltransferase activity);GO:0008455(alpha-1,6-mannosylglycoprotein 2-beta-N-acetylglucosaminyltransferase activity);GO:0009312(oligosaccharide biosynthetic process);GO:0016021(integral component of membrane)	00510(N-Glycan biosynthesis)	*AT2G05320*	beta-1%2C2-N-acetylglucosaminyltransferase II
GH_D11G1591	GO:0005770(late endosome);GO:0006886(intracellular protein transport);GO:0030904(retromer complex);GO:0042147(retrograde transport, endosome to Golgi)	04144(Endocytosis)	*AT3G52300*	ATP synthase D chain
GH_D11	GO:0004345(glucose-6-phosphate dehydrogenase	00030(Pentose phosphate	*AT5G35790*	glucose-6-phosphate

G1592	activity);GO:0005515(protein binding);GO:0006006(glucose metabolic process);GO:0009051(pentose-phosphate shunt, oxidative branch);GO:0009507(chloroplast);GO:0009570(chloroplast stroma);GO:0050661(NADP binding);GO:0055114(oxidation-reduction process)	pathway);00480(Glutathione metabolism)		dehydrogenase 1
GH_D11 G1595	GO:0005768(endosome);GO:0006904(vesicle docking involved in exocytosis);GO:0007032(endosome organization);GO:0007040(lysosome organization);GO:0030674(protein binding, bridging);GO:0030897(HOPS complex);GO:0035542(regulation of SNARE complex assembly)	NA	*AT2G05170*	vacuolar protein sorting 11
GH_D11 G1596	GO:0003674(molecular_function);GO:0005737(cytoplasm);GO:0008150(biological_process)	NA	*AT3G07760*	Sterile alpha motif (SAM) domain-containing protein
GH_D11 G1597	GO:0000155(phosphorelay sensor kinase activity);GO:0000160(phosphorelay signal transduction system);GO:0004673(protein histidine kinase activity);GO:0004721(phosphoprotein phosphatase activity);GO:0005034(osmosensor activity);GO:0005515(protein binding);GO:0005789(endoplasmic reticulum membrane);GO:0005886(plasma membrane);GO:0006970(response to osmotic stress);GO:0009414(response to water deprivation);GO:0009636(response to toxic substance);GO:0009651(response to salt stress);GO:0009736(cytokinin-activated signaling	04075(Plant hormone signal transduction)	*AT5G35750*	histidine kinase 2

	pathway);GO:0009737(response to abscisic acid);GO:0009884(cytokinin receptor activity);GO:0009909(regulation of flower development);GO:0010029(regulation of seed germination);GO:0010087(phloem or xylem histogenesis);GO:0010150(leaf senescence);GO:0010271(regulation of chlorophyll catabolic process);GO:0016021(integral component of membrane);GO:0019900(kinase binding);GO:0034757(negative regulation of iron ion transport);GO:0042802(identical protein binding);GO:0043424(protein histidine kinase binding);GO:0048509(regulation of meristem development);GO:0048831(regulation of shoot system development);GO:0070417(cellular response to cold);GO:0071215(cellular response to abscisic acid stimulus);GO:0080117(secondary growth)			
GH_D11G1598	GO:0003700(DNA binding transcription factor activity);GO:0005634(nucleus);GO:0006355(regulation of transcription, DNA-templated);GO:0043565(sequence-specific DNA binding)	04075(Plant hormone signal transduction)	*AT5G48150*	GRAS family transcription factor
GH_D11G1599	GO:0003700(DNA binding transcription factor activity);GO:0005634(nucleus);GO:0006351(transcription, DNA-templated);GO:0006355(regulation of transcription, DNA-templated);GO:0008270(zinc ion binding);GO:0009863(salicylic acid mediated signaling pathway);GO:0043565(sequence-specific DNA	04626(Plant-pathogen interaction)	*AT2G04880*	zinc-dependent activator protein-1

	binding);GO:0045893(positive regulation of transcription, DNA-templated)			
GH_D11G1600	GO:0003674(molecular_function);GO:0005634(nucleus);GO:0008150(biological_process)	NA	*AT3G07790*	DGCR14-like protein
GH_D11G1601	GO:0004674(protein serine/threonine kinase activity);GO:0005829(cytosol);GO:0006468(protein phosphorylation);GO:0035556(intracellular signal transduction)	NA	*AT5G55560*	Protein kinase superfamily protein
GH_D11G1602	GO:0004797(thymidine kinase activity);GO:0006259(DNA metabolic process);GO:0046104(thymidine metabolic process)	00240(Pyrimidine metabolism)	*AT3G07800*	Thymidine kinase
GH_D11G1604	GO:0005575(cellular_component);GO:0019863(IgE binding);GO:0045490(pectin catabolic process);GO:0047911(galacturan 1,4-alpha-galacturonidase activity)	00040(Pentose and glucuronate interconversions)	*AT3G07820*	Pectin lyase-like superfamily protein
GH_D11G1608	GO:0003674(molecular_function);GO:0005634(nucleus);GO:0008150(biological_process)	NA	*AT5G35732*	hypothetical protein
GH_D11G1609	GO:0004842(ubiquitin-protein transferase activity);GO:0019005(SCF ubiquitin ligase complex);GO:0031146(SCF-dependent proteasomal ubiquitin-dependent protein catabolic process)	NA	*AT3G07870*	F-box and associated interaction domains-containing protein
GH_D11G1610	GO:0003674(molecular_function);GO:0005634(nucleus);GO:0008284(positive regulation of cell proliferation);GO:0009506(plasmodesma)	NA	*AT3G07870*	F-box and associated interaction domains-containing protein
GH_D11G1614	NA	NA	-	-
GH_D11G1615	GO:0003700(DNA binding transcription factor activity);GO:0005634(nucleus);GO:0007165(signal	NA	*AT5G55390*	ENHANCED DOWNY MILDEW 2

	transduction);GO:0009911(positive regulation of flower development);GO:0010228(vegetative to reproductive phase transition of meristem);GO:0050832(defense response to fungus)			
GH_D11G1616	GO:0004046(aminoacylase activity);GO:0005737(cytoplasm);GO:0005773(vacuole);GO:0005783(endoplasmic reticulum);GO:0005794(Golgi apparatus);GO:0010043(response to zinc ion)	00220(Arginine biosynthesis)	*AT4G38220*	Peptidase M20/M25/M40 family protein
GH_D11G1618	GO:0003743(translation initiation factor activity);GO:0005634(nucleus);GO:0005829(cytosol);GO:0006413(translational initiation)	03013(RNA transport)	*AT2G04520*	Nucleic acid-binding%2C OB-fold-like protein
GH_D11G1620	GO:0001530(lipopolysaccharide binding);GO:0005576(extracellular region);GO:0005615(extracellular space);GO:0005773(vacuole);GO:0005774(vacuolar membrane);GO:0008289(lipid binding);GO:0010468(regulation of gene expression);GO:1903409(reactive oxygen species biosynthetic process)	NA	*AT1G04970*	lipid-binding serum glycoprotein family protein
GH_D11G1622	GO:0005634(nucleus);GO:0043407(negative regulation of MAP kinase activity)	NA	*AT2G04550*	indole-3-butyric acid response 5
GH_D11G1623	NA	NA	-	-
GH_D11G1625	GO:0003674(molecular_function);GO:0008150(biological_process)	NA	*AT1G62422*	hypothetical protein
GH_D11G1628	GO:0003674(molecular_function);GO:0006886(intracellular protein transport);GO:0016192(vesicle-mediated transport);GO:0030117(membrane coat);GO:0035690(cellular	NA	*AT5G11490*	adaptin family protein

	response to drug)			
GH_D11G1631	GO:0009693(ethylene biosynthetic process);GO:0051213(dioxygenase activity)	00270(Cysteine and methionine metabolism)	*AT1G05010*	ethylene-forming enzyme
GH_D11G1632	GO:0005737(cytoplasm);GO:0008150(biological_process)	NA	*AT4G03965*	RING/U-box superfamily protein
GH_D11G1634	GO:0005515(protein binding);GO:0005545(1-phosphatidylinositol binding);GO:0005634(nucleus);GO:0005794(Golgi apparatus);GO:0005905(clathrin-coated pit);GO:0006897(endocytosis);GO:0015031(protein transport);GO:0030136(clathrin-coated vesicle);GO:0030276(clathrin binding);GO:0048268(clathrin coat assembly)	NA	*AT1G05020*	ENTH/ANTH/VHS superfamily protein

Supplemental Table 6. Enriched GO terms and KEGG pathways (P < 0.05) of the candidate genes for two stable QTNs and five QEIs

Loci	GO_/pathway_ ID	GO_Term/pathway_name	GO_function	*P* value	Genes
QTNs	GO:0005774	vacuolar membrane	cellular_component	0.00	*GH_A12G0139;GH_A12G0164;GH_D06G1029;GH_A12G0163;GH_A12G0144*
	GO:0004675	transmembrane receptor protein serine/threonine kinase activity	molecular_function	0.02	*GH_A12G0125;GH_D06G1049*
	GO:0007166	cell surface receptor signaling pathway	biological_process	0.03	*GH_A12G0125;GH_D06G1049*
	GO:0055114	oxidation-reduction process	biological_process	0.04	*GH_D06G1029;GH_D06G1045;GH_A12G0134;GH_A12G0143*
	380	Tryptophan metabolism	/	0.01	*GH_D06G1045;GH_A12G0140*
QEIs	GO:0005929	cilium	cellular_component	0.00	*GH_D13G0607;GH_A01G0498;GH_D13G0606*
	GO:0000723	telomere maintenance	biological_process	0.00	*GH_A01G0477;GH_A05G0167*
	GO:0035242	protein-arginine omega-N asymmetric methyltransferase activity	molecular_function	0.00	*GH_D13G0610;GH_A05G0192*
	GO:0008469	histone-arginine N-methyltransferase activity	molecular_function	0.01	*GH_A05G0192;GH_D13G0610*
	GO:0005975	carbohydrate metabolic process	biological_process	0.01	*GH_D13G0601;GH_A05G0268;GH_A05G0176;GH_A06G1661;GH_A05G4049*
	GO:0005905	clathrin-coated pit	cellular_component	0.02	*GH_D13G0576;GH_A05G0277*
	GO:0006897	endocytosis	biological_process	0.02	*GH_A05G0215;GH_A05G0229;GH_D13G0576*
	GO:0006468	protein phosphorylation	biological_process	0.02	*GH_A05G4066;GH_A05G0264;GH_A06G1663;GH_A05G0211;GH_D13G0566;GH_A05G4069;GH_A05G4068;GH_D13G0578;GH_D13G0572;GH_A05G0261;GH_A05G4074*

GO:0003684	damaged DNA binding	molecular_function	0.02	*GH_A06G1658;GH_A01G0477*
GO:0005794	Golgi apparatus	cellular_component	0.04	*GH_A05G0263;GH_A05G4048;GH_A05G0244;GH_A01G0464;GH_D13G0611;GH_D13G0562;GH_A05G0204;GH_A05G0215;GH_A05G0277;GH_A05G4045;GH_D13G0590;GH_D13G0576;GH_A05G0166*
GO:0031225	anchored component of membrane	cellular_component	0.04	*GH_A05G0268;GH_A05G0230;GH_A06G1660*
GO:0005778	peroxisomal membrane	cellular_component	0.04	*GH_D13G0594;GH_A05G0241*
GO:0009620	response to fungus	biological_process	0.04	*GH_A05G4026;GH_D13G0601*
GO:0004674	protein serine/threonine kinase activity	molecular_function	0.04	*GH_A06G1663;GH_D13G0566;GH_A05G4074;GH_A05G4069;GH_A05G0229;GH_A05G0211;GH_A05G0183;GH_A05G0261;GH_A05G4068;GH_A05G4066;GH_A05G0264*
GO:0004675	transmembrane receptor protein serine/threonine kinase activity	molecular_function	0.04	*GH_D13G0572;GH_D13G0578;GH_A06G1663*
970	Aminoacyl-tRNA biosynthesis	/	0.01	*GH_A01G0466;GH_A01G0468;GH_A01G0465;GH_A05G0236*

Supplemental Table 7. Eight predicted protein–protein interactions involving three QQIs and eight gene pairs

QQI	Gene ID	Swissprot_id	Entry name in Swissprot (Arabidopsis thaliana)	Protein name in Swissprot	Gene ID	Swissprot_id	Entry name in Swissprot (Arabidopsis thaliana)	Protein name in Swissprot
TM10542-by-TM59085	*GH_A05G1385*	Q8RWN9	ODP22(MDC16.5)	Dihydrolipoyllysine-residue acetyltransferase component 2 of pyruvate dehydrogenase complex, mitochondrial	*GH_D06G0320*	Q8H103	G6PIP(PGI1)	Glucose-6-phosphate isomerase 1, chloroplastic
	GH_A05G1405	Q9C9E3	TI11B(TIFY11B)	Protein TIFY 11B	*GH_D06G0301*	Q8GXL7	GAT24(GATA24)	GATA transcription factor 24
	GH_A05G1402	Q94AU2	SEC22	25.3 kDa vesicle transport protein SEC22-1	*GH_D06G0329*	Q8VXX9	BETL1(dl3340w)	Bet1-like protein At4g14600
TM3769-by-TM69636	*GH_A02G0124*	Q8LPQ9	FPA	Flowering time control protein FPA	*GH_D08G2269*	Q9FGZ9	UBL5	Ubiquitin-like protein 5
	GH_A02G0128	Q1PE49	4ON1(F20B18.130)	Protein At-4/1	*GH_D08G2269*	Q9FGZ9	UBL5	Ubiquitin-like protein 5
	GH_A02G0076	Q9LTX9	HSP7G(HSP70-7)	Heat shock 70 kDa protein 7, chloroplastic	*GH_D08G2298*	Q8RX71	BAG4	BAG family molecular chaperone regulator 4
	GH_A02G0076	Q9LTX9	HSP7G(HSP70-7)	Heat shock 70 kDa protein 7, chloroplastic	*GH_D08G2303*	C0LGQ4	MRH1(MDIS2)	Protein MALE DISCOVERER 2
TM48233-by-TM75885	*GH_D11G1591*	Q9FT52	ATP5H(ATPQ)	ATP synthase subunit d, mitochondrial	*GH_D01G0704/ GH_D01G0705*	O80800	MTACP2	Acyl carrier protein 2, mitochondrial

Supplemental Table 8. QTNs for cottonseed protein content detected in the current study and previous studies

The current study			Previous studies			
QTN	Chr.	Position (bp)	Marker loci	Chr.	Position (bp)	Reference
TM11103	A05	23962836	BNL3992	A05	23704276-23704876	Yu et al. (2012)
TM63795	D07	12896010	BNL2734	D07	12488659-12488293	Yu et al. (2012)
TM71935	D09	32343135	BNL1672/BNL3031	D09	32850538-32850780	Song & Zhang (2007)

Supplemental Table 9. RNA-seq data for relative gene expression in 18 Upland cotton TM-1 samples

Loci	Bioinformatics methods	Gene ID	TM1_root	TM1_stem	TM1_leaf	TM1_anther	TM1_epicalyx	TM1_petal	TM1_pistil	TM1_sepal	TM1_torus	TM1_0dpa_ovlue	TM1_1dpa_ovlue	TM1_3dpa_ovlue	TM1_5dpa_ovlue	TM1_10_dpa_ovlue	TM1_20_dpa_ovlue	TM1_10_dpa_fiber	TM1_20_dpa_fiber	TM1_25_dpa_fiber
QTN	GO enrichment	*GH_A12G0125*	8.16	4.32	4.68	5.94	16.93	2.79	2.34	21.11	7.60	2.58	3.71	4.01	1.91	3.05	0.16	1.30	2.31	3.27
		GH_A12G0134	0.95	2.38	3.43	1.34	1.09	1.19	0.66	0.88	1.09	0.24	0.24	4.06	3.85	4.62	3.45	5.36	1.71	3.61
		GH_A12G0139	6.79	6.22	2.41	10.88	5.46	6.48	4.05	5.47	6.26	5.76	5.31	4.45	7.83	9.45	5.37	7.19	13.24	7.64
		GH_A12G0143	136.67	55.26	18.39	47.22	198.82	118.09	68.84	480.00	315.06	158.71	49.34	20.85	35.53	68.91	28.03	27.43	21.84	86.05
		GH_A12G0144	17.59	17.04	34.21	14.73	18.39	15.40	11.36	18.08	26.25	24.49	21.95	13.56	15.64	17.47	11.14	6.11	11.04	10.50
		GH_A12G0163	16.21	15.42	11.35	11.68	10.32	7.74	7.62	20.33	23.05	16.47	11.78	26.29	28.50	16.19	9.76	17.89	10.07	9.71
		GH_A12G0164	10.27	15.24	9.78	4.37	12.80	40.83	5.91	6.17	7.71	15.89	10.36	12.73	15.28	54.31	8.68	19.92	33.42	23.93
		GH_D06G1029	0.37	0.01	0.02	0.00	0.00	0.01	0.00	0.00	0.00	0.00	0.00	0.07	0.10	0.00	0.00	0.12	0.00	0.00
		GH_D06G1045	3.36	0.02	0.38	0.48	0.04	0.33	0.14	0.11	0.03	0.03	0.00	0.05	0.06	0.19	0.17	0.00	0.00	0.00
		GH_D06G1049	**2.02**	**0.64**	**0.59**	**0.03**	**0.04**	**0.00**	**0.00**	**0.02**	**1.54**	**13.85**	**9.05**	**4.18**	**8.01**	**4.16**	**5.53**	**0.59**	**0.00**	**2.71**
	KEGG enrichment	*GH_A12G0140*	5.67	4.85	1.95	3.48	4.80	14.93	1.36	13.19	4.44	8.35	5.88	2.66	1.99	2.73	0.46	1.43	3.24	2.55

		GH_D06G1045	3.36	0.02	0.38	0.48	0.04	0.33	0.14	0.11	0.03	0.03	0.00	0.05	0.06	0.19	0.17	0.00	0.00	0.00
Q EI	GO enrichment	*GH_A01G0464*	5.88	2.77	3.13	1.22	2.08	1.11	1.19	3.28	2.69	8.99	5.09	4.52	7.01	3.82	3.28	2.98	3.34	5.37
		GH_A01G0477	10.80	10.15	9.47	18.78	13.45	14.29	14.76	11.32	10.30	20.14	31.78	60.34	44.09	9.26	5.54	24.04	17.34	8.92
		GH_A01G0498	8.40	1.78	0.85	36.28	3.21	7.15	14.32	7.14	11.99	3.30	8.44	7.35	5.29	4.42	2.64	4.70	2.70	2.30
		GH_A05G0166	3.50	2.93	0.44	0.94	1.74	0.45	0.77	3.46	2.48	1.52	1.76	2.46	2.95	1.87	1.12	3.16	3.77	2.60
		GH_A05G0167	12.85	0.00	0.30	35.02	4.05	0.53	2.24	1.04	1.22	1.38	1.58	0.06	0.00	0.00	0.00	0.21	0.00	1.24
		GH_A05G0176	4.85	1.34	7.39	0.69	0.28	0.00	0.00	1.07	1.24	3.38	2.86	1.80	10.43	2.25	12.44	0.23	0.89	5.38
		GH_A05G0183	12.94	11.38	11.95	11.59	10.53	15.89	12.16	11.55	13.19	17.84	15.77	11.61	13.34	10.51	10.52	4.00	8.92	7.89
		GH_A05G0192	10.52	11.62	10.18	4.18	17.00	3.38	3.97	9.06	17.36	7.24	4.89	8.37	9.00	8.96	8.79	7.25	11.44	4.37
		GH_A05G0204	33.86	28.71	13.33	5.50	43.30	7.94	2.04	18.15	13.50	24.44	24.98	13.19	14.21	19.75	13.33	1.20	1.70	5.57
		GH_A05G0211	1.06	1.39	0.24	0.59	1.03	0.99	0.95	1.24	1.72	1.72	1.08	2.61	3.03	2.28	1.26	3.51	2.14	2.34
		GH_A05G0215	11.86	9.55	3.79	19.39	11.13	12.03	12.62	13.27	14.64	9.92	8.97	8.24	8.93	9.62	4.66	7.35	14.36	9.82
		GH_A05G0229	10.46	8.44	6.51	18.34	36.33	9.80	9.98	13.60	10.91	38.40	60.31	0.87	0.89	39.45	3.75	20.40	42.64	121.31

GH_A05G0230	0.10	0.79	0.19	0.25	0.00	0.60	0.21	0.04	0.26	0.65	0.78	0.87	0.71	0.87	0.04	0.34	2.26	0.90
GH_A05G0241	0.03	0.18	0.02	0.78	0.00	0.00	0.00	0.03	0.35	0.10	0.28	1.57	1.76	0.59	0.47	1.70	0.00	0.47
GH_A05G0244	0.42	0.03	0.04	0.01	0.26	0.02	0.00	0.24	0.52	0.00	0.13	0.62	0.02	0.01	0.00	0.04	0.00	0.05
GH_A05G0261	2.92	1.69	5.62	0.01	0.06	0.06	0.05	0.21	1.56	2.51	0.81	1.92	7.20	1.56	1.54	0.27	0.45	0.30
GH_A05G0263	6.05	5.18	1.83	9.71	4.19	10.66	13.06	7.78	6.64	6.84	5.56	5.46	6.50	6.96	3.58	5.18	9.44	6.79
GH_A05G0264	0.09	0.17	0.42	0.04	1.55	0.03	0.03	0.60	0.00	0.11	0.53	0.28	0.00	0.00	0.00	0.00	0.00	0.09
GH_A05G0268	5.66	8.10	16.23	1.02	9.04	3.59	3.14	6.22	11.13	1.59	2.57	21.77	17.40	22.20	15.40	6.98	7.26	9.08
GH_A05G0277	22.68	107.75	21.94	9.28	39.00	18.83	2.48	46.47	49.05	185.38	101.81	6.88	4.55	33.73	2.63	2.69	4.00	65.70
GH_A05G4026	0.03	0.00	0.00	0.15	0.00	0.00	0.00	0.00	0.00	0.13	0.00	0.31	0.28	0.24	0.00	0.00	4.80	0.60
GH_A05G4045	0.88	0.15	2.00	0.20	4.77	0.53	0.17	4.86	0.59	0.00	0.00	0.00	0.00	0.00	0.00	0.33	3.12	1.18
GH_A05G4048	4.66	1.92	0.41	1.79	0.14	0.67	2.89	0.28	1.77	1.47	1.37	0.45	1.19	6.27	4.57	1.16	7.76	5.05
GH_A05G4049	0.45	1.61	4.30	0.09	18.75	0.73	0.06	2.50	0.75	0.04	0.27	0.00	0.00	0.14	0.01	0.00	0.08	0.31
GH_A05G4066	0.37	4.50	2.46	0.07	3.17	0.05	0.06	2.56	0.97	1.20	1.02	4.63	3.81	1.29	0.20	11.77	2.52	1.22

GH_A05G4068	0.37	4.50	2.46	0.07	3.17	0.05	0.06	2.56	0.97	1.20	1.02	4.63	3.81	1.29	0.20	11.77	2.52	1.22
GH_A05G4069	1.56	4.25	12.17	0.12	4.65	0.20	0.28	1.74	0.86	0.38	1.85	2.91	1.05	0.40	0.38	1.26	0.54	0.96
GH_A05G4074	1.01	1.72	0.06	0.00	0.01	0.07	0.02	0.34	0.49	0.00	0.00	0.03	0.00	0.00	0.00	0.00	0.16	0.25
GH_A06G1658	13.71	15.29	7.39	4.88	7.96	7.44	6.99	8.03	8.68	12.67	14.61	15.72	14.61	8.38	7.88	8.65	11.01	9.82
GH_A06G1660	0.00	0.00	0.00	0.24	0.00	0.00	0.00	0.00	0.00	0.00	0.00	0.00	0.00	0.00	0.00	0.00	0.00	0.00
GH_A06G1661	0.00	0.00	0.00	0.10	0.00	0.00	0.00	0.00	0.00	0.00	0.00	0.00	0.00	0.00	0.00	0.00	0.00	0.41
GH_A06G1663	**4.79**	**2.99**	**2.19**	**2.65**	**0.61**	**0.32**	**0.29**	**0.91**	**2.91**	**5.38**	**3.41**	**6.28**	**6.18**	**6.68**	**7.93**	**2.87**	**3.05**	**5.99**
GH_D13G0562	25.16	21.35	11.30	17.99	20.07	20.10	16.04	35.62	22.63	2.56	3.71	7.28	10.96	7.20	9.12	6.14	8.40	8.01
GH_D13G0566	0.01	0.00	0.00	0.10	0.00	0.00	0.05	0.00	0.00	0.00	0.01	0.00	0.00	0.00	0.00	0.00	0.10	0.00
GH_D13G0572	2.95	6.95	3.09	2.17	21.52	6.48	2.55	11.05	2.03	2.79	1.59	3.70	3.26	1.85	3.26	1.10	0.30	2.74
GH_D13G0576	0.19	0.06	0.00	0.55	0.06	0.00	0.02	0.03	0.04	0.01	0.06	0.04	0.14	0.00	0.00	0.00	0.11	0.00
GH_D13G0578	10.06	9.86	9.81	7.72	38.99	19.81	7.25	20.82	9.14	1.99	2.10	3.74	3.06	4.10	2.90	2.35	5.39	4.26
GH_D13G0590	19.84	21.67	14.49	13.13	9.49	10.73	14.63	26.93	31.14	2.88	3.67	4.59	5.32	23.66	11.98	0.90	17.43	19.08

		GH_D13G0594	16.73	18.61	8.29	6.40	9.09	5.78	6.25	12.22	9.91	9.15	7.42	16.34	17.48	14.60	11.11	17.15	7.62	10.08
		GH_D13G0601	**6.71**	**6.22**	**1.47**	**1.34**	**1.28**	**1.07**	**0.88**	**4.33**	**2.62**	**0.81**	**0.46**	**0.41**	**2.49**	**10.55**	**20.86**	**4.49**	**7.13**	**9.77**
		GH_D13G0606	7.45	1.06	0.06	0.03	0.12	0.11	0.18	0.13	1.41	0.00	0.00	0.03	0.00	0.00	0.00	0.00	0.15	0.06
		GH_D13G0607	68.75	19.91	10.87	35.97	31.99	28.84	45.93	17.44	16.93	13.75	21.41	5.31	6.86	5.62	6.64	1.98	19.45	16.74
		GH_D13G0610	5.54	3.64	3.08	2.77	2.84	2.00	1.93	2.97	3.89	13.99	13.00	9.41	10.19	12.43	5.41	8.09	4.80	6.06
		GH_D13G0611	2.23	2.50	1.30	1.44	2.62	1.68	1.49	1.52	1.95	0.07	0.42	0.80	2.36	0.90	0.58	1.82	1.84	0.74
	KEGG enrichment	*GH_A01G0465*	94.03	0.97	0.00	0.04	0.00	0.09	0.05	0.00	0.00	0.00	0.00	0.00	0.00	0.09	0.00	0.00	0.00	0.00
		GH_A01G0466	25.96	1.01	1.23	0.00	0.22	0.00	0.15	0.05	0.00	0.00	0.00	0.41	2.90	0.00	0.05	7.08	0.00	0.00
		GH_A01G0468	0.04	0.00	0.00	0.00	0.46	0.00	0.00	0.00	0.00	0.00	0.00	0.00	0.00	0.00	0.00	0.00	0.00	0.00
		GH_A05G0236	**0.99**	**0.03**	**0.00**	**0.13**	**0.38**	**0.46**	**1.96**	**7.62**	**10.77**	**17.22**	**20.62**	**17.34**	**12.16**	**0.75**	**0.72**	**0.12**	**0.00**	**0.25**
QQI	Protein interaction	*GH_A02G0076*	35.44	48.56	79.79	7.84	39.61	6.61	5.85	34.85	43.49	41.07	39.42	43.49	57.77	41.77	20.41	34.20	19.97	24.76
		GH_A02G0124	6.14	5.65	1.40	6.25	6.53	3.67	3.56	6.97	7.78	1.34	2.66	3.27	4.06	6.81	3.66	3.89	6.30	5.21
		GH_A02G0128	0.04	0.04	0.65	0.31	0.14	0.48	0.12	0.38	0.18	0.47	0.28	0.04	0.33	0.24	0.22	0.00	0.00	1.08

GH_A05G1385	38.90	35.72	28.83	32.55	25.06	50.49	48.09	68.58	65.36	4.76	4.81	17.40	37.76	21.17	10.12	20.70	43.71	25.83
GH_A05G1402	17.15	13.68	17.84	8.64	11.37	8.69	6.29	14.02	16.03	15.05	12.62	11.17	20.12	12.42	6.78	11.89	17.13	10.13
GH_A05G1405	25.52	8.70	6.19	14.94	182.48	26.95	16.72	78.03	397.03	12.33	7.61	1.76	1.57	2.82	0.96	2.13	4.60	5.43
GH_D01G0704	70.52	61.11	273.66	75.80	90.86	67.67	82.65	65.36	73.47	69.45	77.73	91.60	101.63	100.49	53.58	30.04	39.62	29.04
GH_D01G0705	15.80	21.83	40.54	23.25	28.80	35.23	33.95	25.30	25.21	33.86	41.37	66.56	61.81	37.43	8.40	56.50	49.19	34.16
GH_D06G0301	16.00	13.28	9.44	0.02	8.96	0.00	0.11	0.23	1.06	5.46	6.43	3.86	3.83	8.65	7.09	0.00	0.35	2.23
GH_D06G0320	38.23	37.83	20.30	12.96	14.09	11.23	18.75	30.18	35.46	21.11	17.71	51.48	49.38	58.40	20.66	56.92	40.46	30.61
GH_D06G0329	24.57	20.71	29.95	27.25	35.65	32.13	44.47	22.29	20.87	43.85	36.01	27.34	22.50	11.15	6.95	8.42	11.67	9.36
GH_D08G2269	0.00	0.38	1.84	0.24	0.49	0.11	0.00	0.10	0.45	0.00	0.64	0.30	1.31	22.20	0.79	0.00	0.00	0.00
GH_D08G2298	0.87	1.52	0.97	0.00	2.53	0.11	0.00	0.93	0.52	0.55	2.30	0.89	0.44	0.27	0.00	0.15	0.49	0.88
GH_D08G2303	33.85	85.97	1.99	0.50	8.38	2.01	0.81	5.94	4.63	3.95	3.40	5.27	5.31	4.37	3.69	7.45	315.62	143.95
GH_D11G1591	5.93	3.54	2.35	3.13	3.85	3.18	3.76	5.61	3.29	9.41	4.33	3.32	2.74	3.90	2.34	1.14	9.41	8.56

The bold font is the expression information of four genes that are preferentially expressed in at least four of the six ovule tissues in all 18 samples
